# I-ATAC: interactive pipeline for the management and pre-processing of ATAC-seq samples

**DOI:** 10.7717/peerj.4040

**Published:** 2017-11-22

**Authors:** Zeeshan Ahmed, Duygu Ucar

**Affiliations:** 1 Department of Genetics and Genome Sciences, University of Connecticut Health Center, Farmington, CT, United States of America; 2 The Jackson Laboratory For Genomic Medicine, Farmington, CT, United States of America

**Keywords:** ATAC-seq, Genomics, Data, Management, Pipeline, Pre-processing

## Abstract

Assay for Transposase Accessible Chromatin (ATAC-seq) is an open chromatin profiling assay that is adapted to interrogate chromatin accessibility from small cell numbers. ATAC-seq surmounted a major technical barrier and enabled epigenome profiling of clinical samples. With this advancement in technology, we are now accumulating ATAC-seq samples from clinical samples at an unprecedented rate. These epigenomic profiles hold the key to uncovering how transcriptional programs are established in diverse human cells and are disrupted by genetic or environmental factors. Thus, the barrier to deriving important clinical insights from clinical epigenomic samples is no longer one of data generation but of data analysis. Specifically, we are still missing easy-to-use software tools that will enable non-computational scientists to analyze their own ATAC-seq samples. To facilitate systematic pre-processing and management of ATAC-seq samples, we developed an interactive, cross-platform, user-friendly and customized desktop application: interactive-ATAC (I-ATAC). I-ATAC integrates command-line data processing tools (FASTQC, Trimmomatic, BWA, Picard, ATAC_BAM_shiftrt_gappedAlign.pl, Bedtools and Macs2) into an easy-to-use platform with user interface to automatically pre-process ATAC-seq samples with parallelized and customizable pipelines. Its performance has been tested using public ATAC-seq datasets in GM12878 and CD4+T cells and a feature-based comparison is performed with some available interactive LIMS (Galaxy, SMITH, SeqBench, Wasp, NG6, openBIS). I-ATAC is designed to empower non-computational scientists to process their own datasets and to break to exclusivity of data analyses to computational scientists. Additionally, I-ATAC is capable of processing WGS and ChIP-seq samples, and can be customized by the user for one-independent or multiple-sequential operations.

## Introduction

Assay for Transposase-Accessible Chromatin with high throughput Sequencing (ATAC-seq) is developed to profile chromatin accessibility from small cell numbers, making it uniquely suited to study epigenomic profiles of human clinical samples with a systems biology approach (Buenrostro et al., 2013). ATAC-seq generates libraries via a simple two-step protocol using hyperactive Tn5 transposase, which inserts itself to open chromatin sites and generate double-strand breaks. ATAC-seq is attracting a growing interest in genomics applications due to its simple protocol, high sensitivity, and low expectations for starting material amounts (500–50,000 cells) (Tsompana & Buck, 2014). Therefore, data processing and management of samples generated by this new assay are becoming an important first step to studying the open chromatin sites in diverse human cells.

The traditional way of next generation sequencing (NGS) data pre-processing is based on running a series of command-line applications, which requires good programming skills and the ability to work in the UNIX environment. Several integrated platforms exist to help in managing and building pipelines for NGS data pre-processing e.g., *Galaxy* (Scholtalbers et al., 2013; Giardine et al., 2005), *SMITH* (Venco et al., 2014), *SeqBench* (Dander et al., 2014), *Wasp* (McLellan et al., 2012), *NG6* (Mariette et al., 2012), *openBIS* (Bauch et al., 2011), etc.

*Galaxy* (Scholtalbers et al., 2013; Giardine et al., 2005) is an open source (https://galaxyproject.org/), platform independent web application, developed in the Python programming language. It is an interactive large-scale platform for NGS data analysis, which takes Illumina-generated FASTQ files as input and produces BED files as output. It not only provides ATAC-seq data processing but also some other kinds of data processing (e.g., WGS, ChIP-seq, etc.). However, it doesn’t provide visualization feature, direct download and use access, as well as requires good bioinformatics, database management and web development skills for the deployment and configuration of *Galaxy*. It is one of the widely used platforms of today with maximum data processing features, which are even used by some other platforms as well, but again it is not easily possible to deploy *Galaxy* without a good bioinformatics and informatics skill set. *SMITH* (Venco et al., 2014) is another open source (https://bitbucket.org/yuriy_vaskin/smith), platform independent web application, developed in the JAVA programming language. It is a laboratory information management system (LIMS), which recommends *Galaxy* (Scholtalbers et al., 2013; Giardine et al., 2005) for the processing of NGS data. *SeqBench* (Dander et al., 2014) is also an open source, platform independent web application (http://www.icbi.at/software/seqbench/seqbench.shtml), developed in the JAVA programming language. It claims to be an integrated solution for the management and analysis of exome sequencing data, which recommends *SIMPLEX platform for the* processing of NGS data. *Wasp* (McLellan et al., 2012) is a data management system for managing and analyzing genomic data. It is an open source (http://www.icbi.at/software/seqbench/seqbench.shtml), platform independent web application, developed mainly in the PHP and Perl programming languages. *NG6* (Mariette et al., 2012) is an integrated next generation sequencing storage and processing environment, which recommends *Ergatis* (Orvis et al., 2010) for the processing of NGS data. It is an open source, platform independent web application, developed mainly in the PHP programming language (https://mulcyber.toulouse.inra.fr/plugins/mediawiki/wiki/ng6/index.php/Main_Page). *OpenBIS* (Bauch et al., 2011) is a flexible framework for managing and analyzing complex data in biology research. It is an open source, platform independent web application, developed mainly in the AJAX programming language. Moreover, like *Galaxy* (Scholtalbers et al., 2013; Giardine et al., 2005), all these applications (*SMITH, SeqBench, Wasp, NG6, openBIS*) do not provide visualization of processed NGS data, direct download and use features, as well as requires good bioinformatics, database management and web development skills for the deployment and configurations. Moreover, these platforms offer solutions for the user with large scale datasets but do not address those which are small scale and for which the user would like to have a quick and direct solution for ATAC-seq samples pre-processing. These interactive, published NGS data management and processing solutions are web based and open source but require web development and informatics skills to run the applications by first creating virtual/physical web and database servers, then by creating a relational database, setting up user accounts and access, then hosting the website as well as many related small and complex tasks. Once the applications are in running condition, they require sufficient bioinformatics skills to download and set up compilers/interpreters (e.g., Python, Java, Perl, etc.) and genomics tools with compatible versions, paths, reference genomes, adapters, etc. Additionally, if users would like to use the pre-existing setups, they will lose the privacy and confidentiality of NGS data and associated metadata.

**Figure 1:**
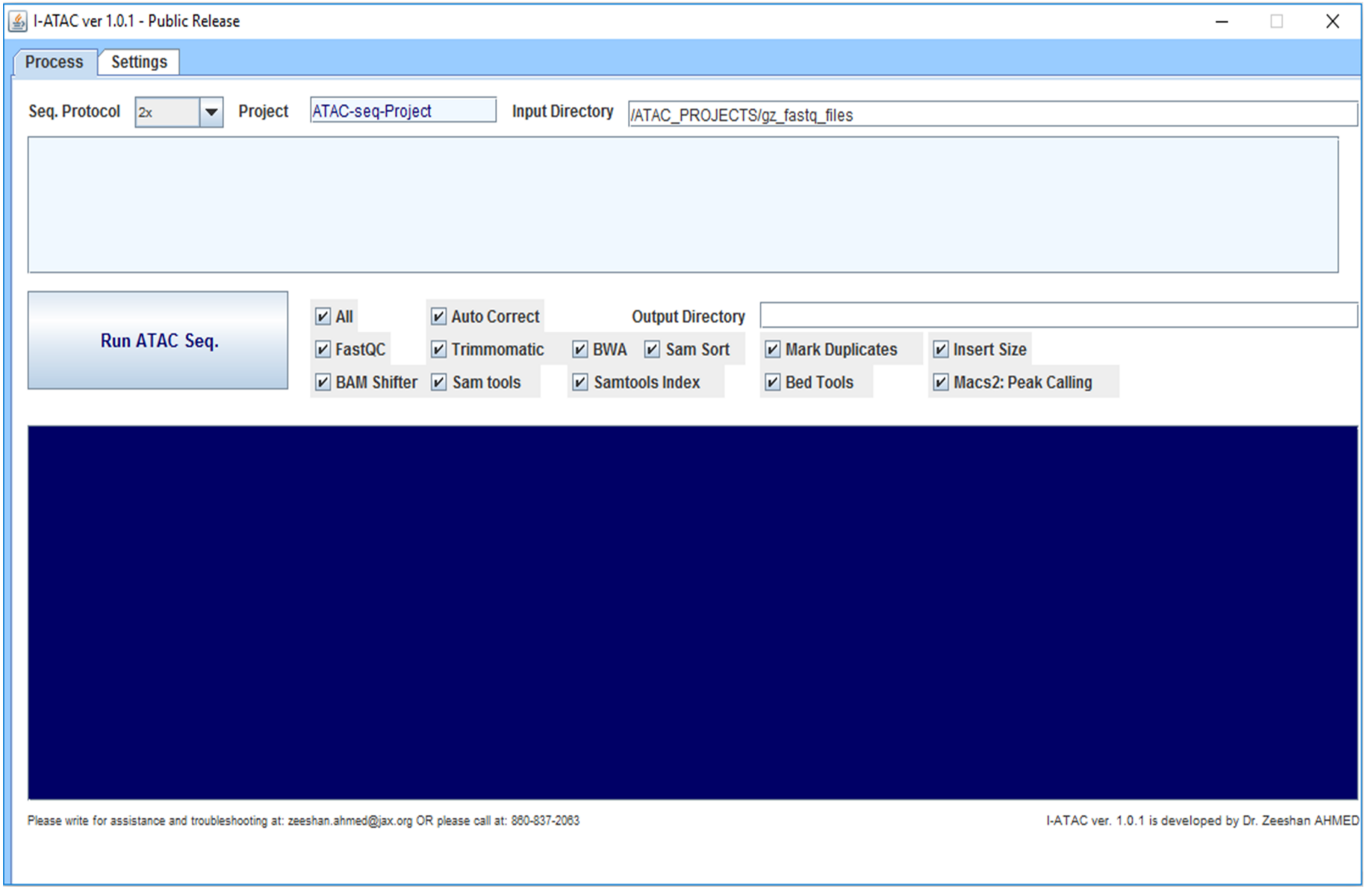
Graphical User Interface of *Processing* module of I-ATAC: Create and run data processing jobs. The figure presents the module *Process* (screen shot taken using Windows 10) of I-ATAC, which enables the user to input sequence protocol (1× for single and 2× for paired), project and main directory name, samples data input directory and run ATAC-seq pipeline. It also enables the user to customize the pipeline by choosing one or multiple integrated applications and setting the order of pre- and post-requisites.

**Figure 2:**
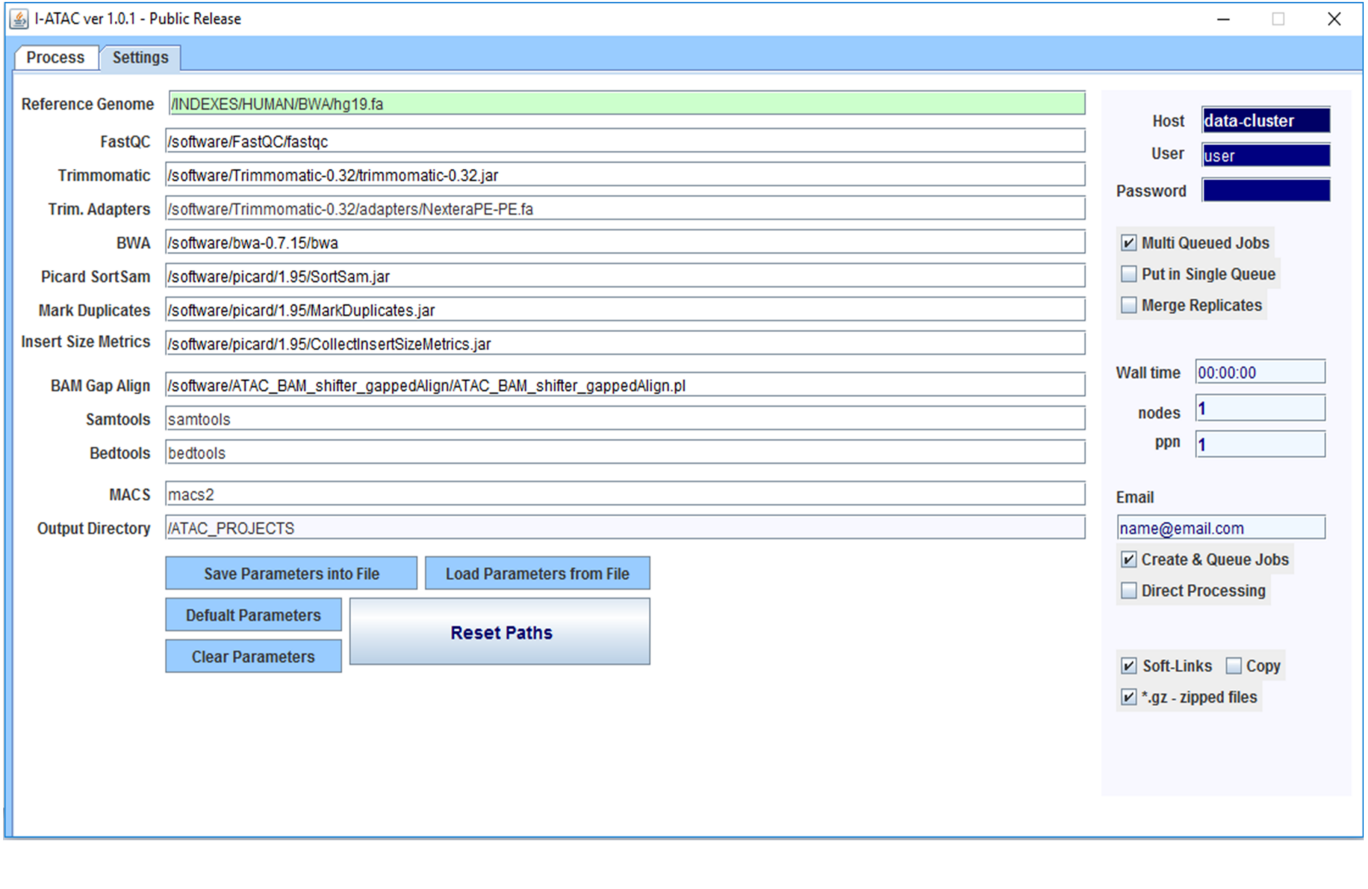
Graphical User Interface of *Settings* module of I-ATAC: Set parameters and user credentials. The figure presents the module *Settings* (screen shot taken using Windows 10) of the I-ATAC, which enables user to following tasks: (1) setting parameters for integrated applications and reference genome; (2) saving parameters/settings details on to file; (3) loading pre-existing settings from already saved file; (4) setting default parameters; (5) clearing parameters (fields); (6) reset paths; (7) giving user login credentials (based on local computer or data cluster); (8) deciding to process multiple samples in parallel jobs (without merging replicates); (9) setting script features which includes wall time, nodes, PPN and email, decide to create only or que job as well; (10) deciding to create soft links of FASTQ files or Copy in to project directory before starting pre-processing; and (11) informing the system about the use of FASTQ compressed and uncompressed files.

However, there is no open source software that is standalone, desktop based interactive and solely designed for ATAC-seq samples processing and easy-to-use, which enables biologists with no programming experience to analyze their ATAC-seq samples (Table 1). To facilitate data analysis for the scientists who generate data, we have developed interactive and cross-platform software for the processing of ATAC-seq samples, namely Interactive-ATAC (I-ATAC) (Ahmed & Ucar, 2017). We have applied I-ATAC to several in-house and publicly available ATAC-seq datasets for GM12878 and CD4+T cells to process these samples with the help of an easy-to-use software platform. Along with the recommended settings for tools that we use for pre-processing, the only input to the I-ATAC is the path to the directory where ATAC-seq samples (single or paired end) can be found (FASTQ files). With just a one click operation, it automatically connects and interacts with the data cluster to locate sample data files, write command-line instructions, manipulate (copy, paste, unzip) compressed input files, load compilers & interpreters, call applications, creates shell scripts, generate multiple, parallel, sequential and customized data analysis pipelines, submit and queue jobs, create output directory structure, process data files, place output data files in relevant directories, and set notifications and disconnects to the connected data cluster. The graphical user interface (GUI) of I-ATAC (Figs. 1 and 2) is designed for simplicity and ease by following human computer interaction (HCI) guidelines (Ahmed, Zeeshan & Dandekar, 2014). I-ATAC is divided in to two modules: (1) *Process* and (2) *Settings*. The *Process* module is for generating and running the pipeline, whereas *Settings* is for setting the parameters of the applications and directory paths (GUI details are given in Supplemental Information 1).

**Table 1:** Comparison between Galaxy, SMITH, SeqBench, Wasp, NG6, openBIS and I-ATAC. The table provides features based comparative analysis of different applications with I-ATAC.

Applications/Features	Galaxy	SMITH	SeqBench	Wasp	NG6	openBIS	I-ATAC
Platform independent	X	X	X	X	X	X	X
Web application	X	X	X	X	X	X	–
Desktop application	–	–	–	–	–	–	X
Command-line application	–	–	–	–	–	–	–
Requires good knowledge of bioinformatics to use	X	–	X	X	X	X	–
Requires good knowledge of computer science to use	X	X	X	X	X	X	X
ATAC-seq data pre-processing	X	Use Galaxy	SIMPLEX pipeline EXOME	–	Ergatis	–	X
Open source	X	X	X	X	X	X	X
Programming language	Python programming language	JAVA	JAVA	Perl, PHP	PHP	AJAX	JAVA
Input files FASTQ	X	X	X	X	–	–	X
User friendly	X	X	X	–	–	–	X
Illumina instrument	X	X	X	X	–	–	X
Data management	X	X	X	X	X	X	X
Create soft links	–	–	–	–	–	–	X
Copy data files	–	–	–	–	–	–	X
Differentiate between compressed and uncompressed FASTQ files	X	–	–	–	–	–	X
Provide data and directory structure	–	–	–	–	–	–	X
Provide options to save, reload, clear and exchange integrated applications parameters	–	–	–	–	–	–	X
Provide embedded run time terminal response	–	–	–	–	–	–	X
Types of output files	BED	–	SNP	–			Trimmed FASTQ, FASTQC reports, SAM, BAM, BED
Create merged replicate, BAM files	–	–	–	–	–	–	X
Customizable ATAC-seq pipeline	–	–	–	–	–	–	X
Provide automatic multiple parallel and sequential data processing and jobs submissions	–	–	–	–	–	–	X
Provide data cluster job, script customization	X	–	–	–	–	–	X
Published, citations	Scholtalbers et al. (2013), Giardine et al. (2005)	Venco et al. (2014)	Dander et al. (2014)	McLellan et al. (2012)	Mariette et al. (2012)	Bauch et al. (2011)	Ahmed & Ucar (2017)
Published, software web links	https://galaxyproject.org/	https://bitbucket.org/yuriy_vaskin/smith	http://www.icbi.at/software/seqbench/seqbench.shtml	http://www.sciencedirect.com/science/article/pii/S0888754312001747	https://mulcyber.toulouse.inra.fr/plugins/mediawiki/wiki/ng6/index.php/Main_Page	http://www.sybit.net/software/1344295-openbis	https://github.com/UcarLab/I-ATAC
Provide visualization	–	–	–	–	–	–	–
Download and use	–	–	–	–	–	–	X
Direct access to data clusters and local machines via SSH	X	–	–	–	–	–	X
Requires extensive setup configurations	X	X	X	X	X	X	–
Main focus ATAC-seq	–	–	–	–	–	–	X

**Notes.**

X shows availability – shows unavailability of that feature

## Methods and Implementation

**Figure 3:**
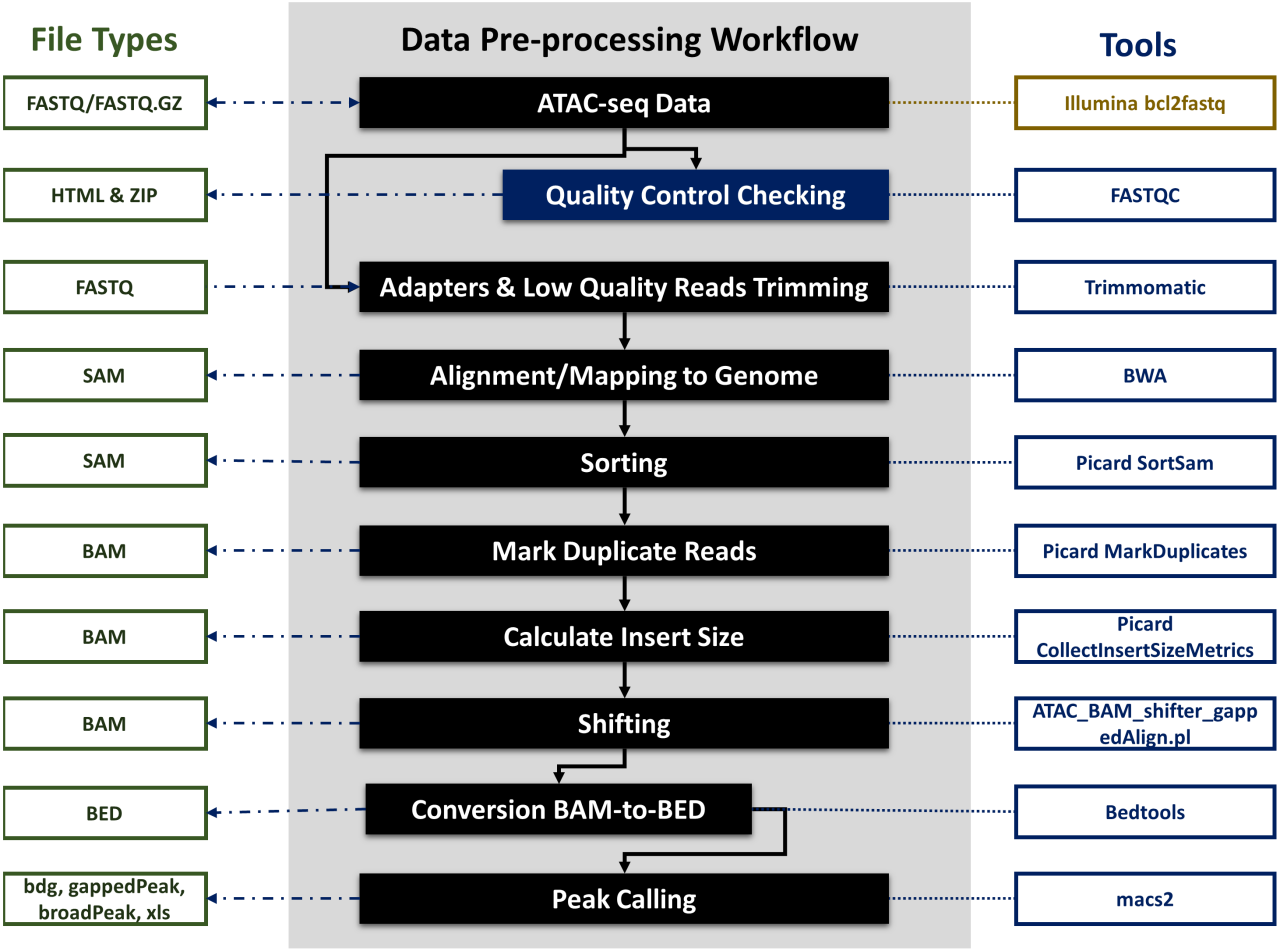
ATAC-seq data pre-processing workflow, file types and integrated tools. The figure explains all the data pre-processing steps, which include input, quality control checking, adapters and low quality reads trimming, mapping to the genome, sorting, marking duplicates, calculating insert size, shifting of reads, conversion of BAM to BED files and peak calling. The figure also explains the input and output of different kinds of data files (FASTQ, FASTQ.GZ, HTML, ZIP, SAM, BAM, BED , BDGm grappedPeak, broadPeak and xls), processed using different bioinformatics third-party tools (FASTQC, Trimmomatic, BWA, Picard, ATAC_BAM_shiftrt_gappedAlign.pl, bedtools and macs2) involved in each pre-processing step.

I-ATAC is based on an I/O redirection framework (*FASTQ, FASTQ.gz, txt, sam, bam, bed, bdg, broadPeak, gappedPeak, xls, pdf and html*) that integrates several publicly available command-line tools within this framework for data quality control, adapter filtering, trimming, alignment, shifting, duplicate read filtering and peak calling (Fig. 3).

Within I-ATAC, we have utilized *FASTQC* for computing the quality statistics. *FASTQC* command-line-based, non-interactive tool which takes FASTQ/SAM/BAM file (compressed/uncompressed) as input, performs modular set of analysis and produces quality statistics (*Basic Statistics, Per base sequence quality, Per tile sequence quality, Per sequence quality scores, Per base sequence content, Per sequence GC content, Per base N content, Sequence Length Distribution, Sequence Duplication Levels, Overrepresented sequences, Adapter Content and Kmer Content*), which are very helpful in analyzing the quality of the sequence data. The output of *FASTQC* is not the input to any other integrated application in I-ATAC but helps in getting quick impression of whether the sequence data has any problem. The output of FASTQC is HTML file and compressed files.

During the first major step of data pre-processing, we integrated *Trimmomatic* (Bolger, Lohse & Usadel, 2014) for the identification and trimming (or clipping) of the adapter and low quality nucleotide sequences. *Trimmomatic* is a command-line-based, non-interactive tool for the trimming of reads using paired-end and single ended data produced by the Illumina next generation sequencing technology. It takes compressed or uncompressed FASTQ (phred-33 and phred-64 quality scores) file as input and mainly performs adapter filtering, sliding window trimming, base cutting (start and end of reads, as well, at specific number) and removes below quality reads. The outcome of *Trimmomatic* are uncompressed filtered FASTQ files. Next, we used Burrows-Wheeler Alignment tool (BWA) (Li & Durbin, 2009) for aligning ATAC-seq reads to a reference genome. BWA takes FASTQ files as input (in our case, the output file “*.fastq_filtered” of *Trimmomatic*) and produces SAM file as output. It implements BWA-backtrack for reading sequence up to 100 bp, and BWA-SW and BWA-MEM algorithms for reading longer sequences between 70 bp to 1 Mbp. It helps in identifying the regions of similarity that may be a significance of functional, structural, evolutionary relationships between the nucleotide sequences (Mount, 2004).

Produced SAM file by the *BWA* is used by the Sequence Alignment/Map (SAM) tools (Li et al., 2009) and *Picard* (https://broadinstitute.github.io/picard/) for generating, processing and viewing “sam” and “bam” files. *SAMtools* perform complex operations at sequence data files, including variant calling, alignment, sorting, indexing, viewing, data extraction and format conversion, whereas *Picard* manipulates high-throughput sequencing (HTS) data and formats such as SAM/BAM/CRAM and VCF. In I-ATAC, we have used *SAMtools* for sorting (based on coordinates) and *Picard* for marking duplicate nucleotide sequences (locates and tags duplicate reads) and calculating insert size (metrics for validating library construction including the insert size distribution and read orientation of paired-end libraries). As the next step, I-ATAC adjusts sequences using *ATAC_BAM_shiftrt_gappedAlign.pl*. It is an open-source Perl script, which can be used to perform read shifting based on the read quality. It takes aligned “bam” file as an input and offsets by 4 bp for the positive strand (sequence containing instructions for building a protein) and –5 bp for the negative strand (merely contains the complementary sequence and according to the base-pairing rules it is not normally transcribed into RNA nor translated into protein). If the read is on the positive strand (as determined by the SAM flag) it will add 4 to the start, and subtract 5 from the partner start. If the read is on the negative strand, 5 will subtracted from its start and 4 will be added to its mate start. The length and read type will be adjusted in both cases and the read and quality string trimmed appropriately. This is done because Tn5 has a 9 bp binding site, and it binds in the middle. Functionally that means that the DNA had to be accessible at least 4.5 bp on either site of the insertion. Outcome *ATAC_BAM_shiftrt_gappedAlign.pl* is converted into BED format using Browser Extensible Data (BED) tools (Quinlan & Hall, 2010) to identify regions of the genome enriched in ATAC-seq reads (i.e., peaks) that are the putative open chromatin sites (peaks) by using Model-based Analysis of ChIP-Seq (MACS) (Zhang et al., 2008). These peaks can be visualized using frequently used genome browsers (e.g., *USCS*, *IGV*) and can be further processed for annotation and for differential open chromatin detection. Further details of all integrated applications are given in Supplemental Information 1.

To manage pre-processed data, I-ATAC creates a proposed directory structure, which is:

**Table:** 

*ATACseq_Projects*
→*Project_Name*
→→*Sample_A_R1_Sample_A_R2*
→→→*fastQC*
→→→*trimmomatic*
→→→→*bwa*
→→→→→*macs2*
→*mergedreplicated*
→→*macs2*

The directory structure is automatically created in the data cluster, before data pre-processing starts. All the quality reports (“zip” and “html” files) generated by the FASTQC software, are placed in the “fastQC” sub-directory. All trimmed and filtered “FASTQ” files, generated by the *Trimmomatic*, are placed in the “trimmomatic” sub-directory. All the sorted, shifted “sam”, indexed “bam” and “bed” files are placed in the “bwa” sub-directory. All the observed peak files are placed in the “macs2” sub-directory. The nested directory structure provides an organized and modular storage for the multi-level ATAC-seq data analysis pipeline.

**Figure 4:**
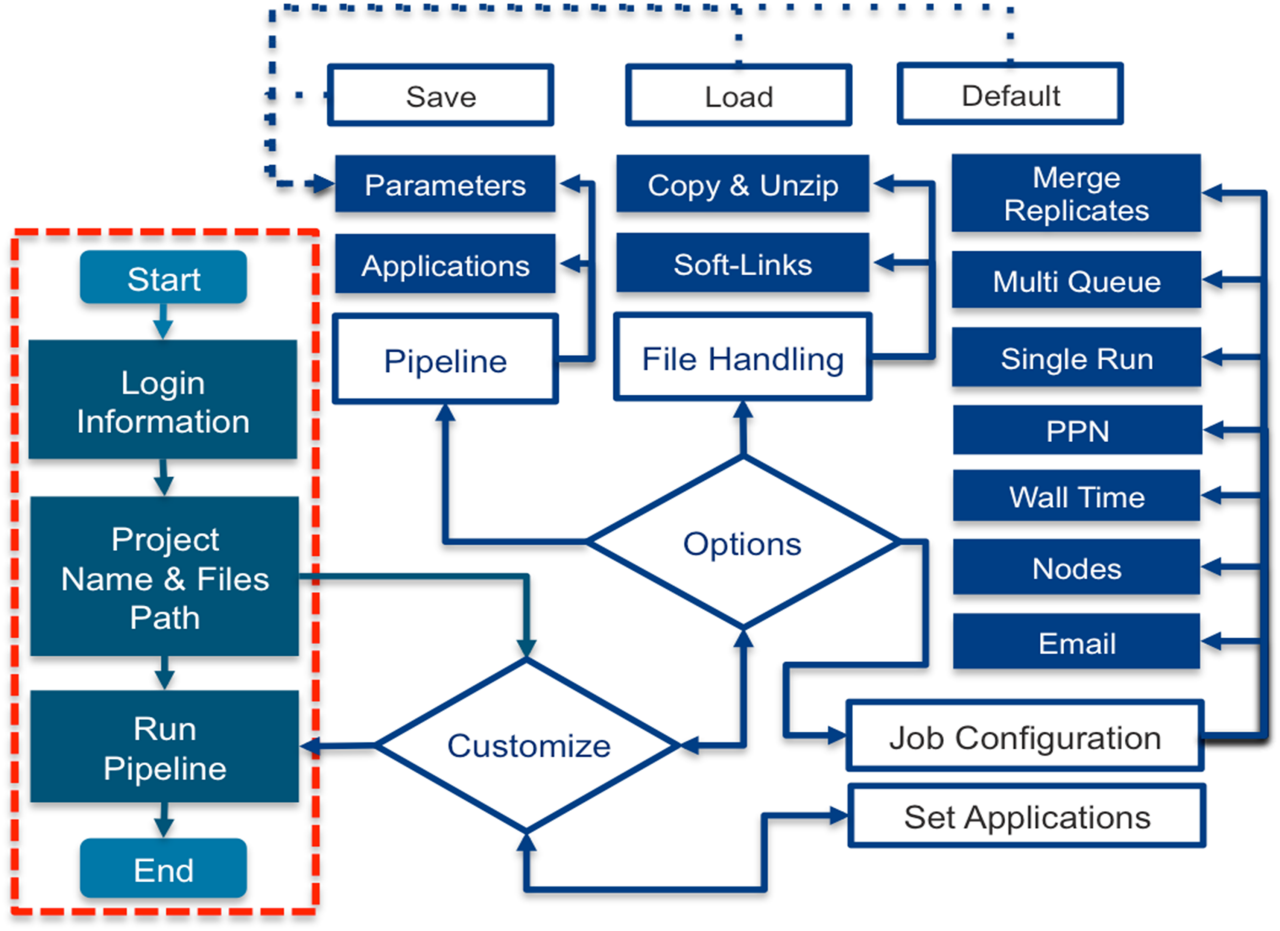
Direct and customized components workflow of I-ATAC. The figure explains the user system interaction. Following default workflow, the user needs to provide network/data cluster credentials, add information about data location and set paths to the integrated and called applications. Following the customized workflow, the user can customize pipeline with different settings which can be saved and reloaded, chose between copying and creating soft links of data files (FASTQ/FASTQ/GZ), and configure jobs with the options: merging replicates, running multiple scripts as one job or multiple parallel scripts, set PPN, wall time, nodes, email, and decide about merging replicates.

The default work flow of I-ATAC is very simple (Fig. 4), as it requires login information, project name and paths to the sample files as input; however, pipeline operations can be customized by choosing the applications between *FASTQC, Trimmomatic, BWA, Sam Sort, Mark Duplicates, Insert Size, BAM Shifter, SAM tools, SAM tools index* and *BED tools*. These could be used, for example, if the user is only interested in having FASTQC reports or the trimming of low quality reads and adapters, or if the user has already trimmed filtered FASTQ files but would like to map to the reference genome only or may be only interested in generating BED files from BAM and peak calling, etc. To avoid exceptions, the system will not let the user select any application without selecting its pre-requisites. The user can remotely handle sample data files for processing by either keeping them in the same parent directory and putting only pre-processed results in the main project and sub-project directories or by first copying compressed files into the project directory, unzipping them and then processing them. I-ATAC supported customization can be very helpful, especially in troubleshooting situations where due to any reason either pipeline could not fully execute or if there is already data existing in a form which does not require all steps of ATAC-seq pipeline. This customization can save time and computational resources.

I-ATAC is capable of connecting UNIX, Linux, Windows and MAC based servers, and intranet connected and local systems via secure shell (SSH) cryptographic network protocol*.* It supports UNIX/Linux based portable batch systems (PBS) for the scheduling and submission of jobs by allocating computing resources, which includes time, nodes, CPUs and memory. Currently the available version does not support the cloud (e.g., Amazon or Google, etc).

**Figure 5:**
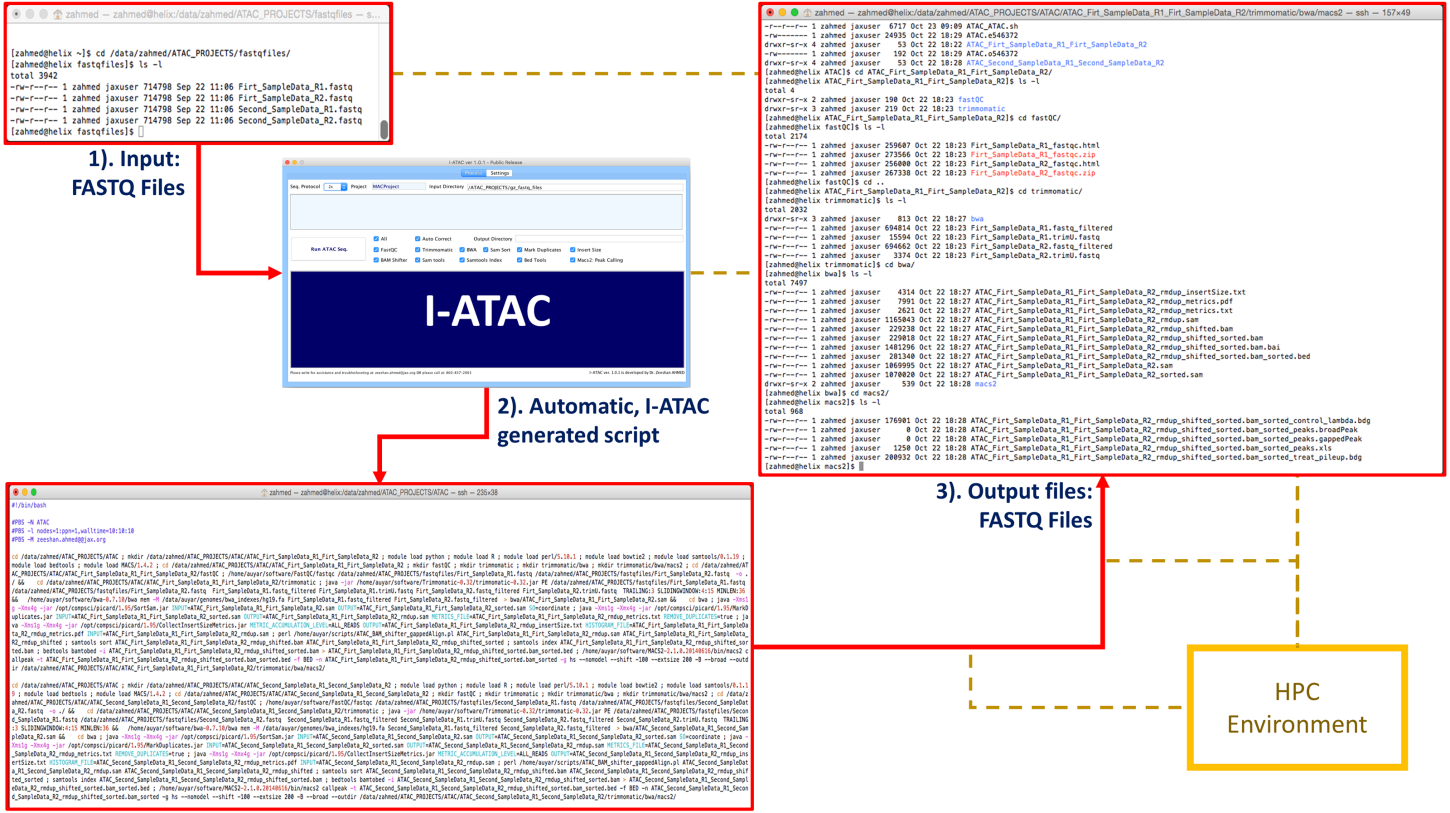
I-ATAC sample data input, script generation and outcome. The figure presents the examples ATAC-seq data input to the I-ATAC as FASTQ files, automatically generated script by the I-ATAC and produced output files, uising connected high performance computing (HPC) environment.

I-ATAC allows the user to automatically create and submit one sequential job (UNIX based secure shell scripts) for multiple samples, as well as creating and submitting multiples parallel jobs for multiple samples (one for each). Having information about input samples with set parameters when the user runs the pipeline, at first I-ATAC confirms the user entered information, which includes the user-entered project name, set wall time, number of nodes, number of PPN, input and output files locations (Fig. 5). Having confirmation from the user, I-ATAC first connects to the data cluster, locates sample data files and asks for a second validation from the user about picked information, which includes names and numbers of samples located. Receiving second confirmation from the user, I-ATAC writes Linux commands, creates directory structure, load modules, compilers and interpreters, calls integrated applications, creates shell scripts, customizes pipelines, sets processing parameters, sets wall time, sets status notifications, submits and queues jobs, process data files, performs file management, and disconnects to the data cluster (Fig. 5). Step-by-step operational details are given in Supplemental Information 1.

I-ATAC not only supports data pre-processing of ATAC-seq samples, but it can also be applied for the pre-processing of Whole Genome Sequencing (WGS) and ChIP-seq (Horak & Snyder, 2002) data as well. I-ATAC is designed following software engineering principles for the sustainable bioinformatics software implementation (Ahmed, Zeeshan & Dandekar, 2014). It is a Java-based desktop application, which requires Java Runtime Environment and all integrated applications to be installed in the data cluster as well as the reference genomes (e.g., human hg19/hg38, etc.) that will be used for the alignment.

## Data Processing and Results

In order to validate the performance of I-ATAC, we present a case study. We have downloaded SRR891275, SRR891276, SRR891277 and SRR891278, publically available data from NCBI Sequence Read Architecture (SRA) in the form of FASTQ files and their respective BAM and BED files (Table 2). These files are of CD4+T cell from GSE47753 dataset (Buenrostro et al., 2013). The published BAM and BED files are based upon the regions of interest. We have processed FASTQ files and generated individual and merged BAM and BED files using I-ATAC and then compared our results with published results. The size of the FASTQ files were variable, SRR891275 (398 MB, 387 MB), SRR891276 (553 MB, 541 MB), SRR891277 (538 MB, 525 MB) and SRR891278 (455 MB, 446 MB). While data processing, using I-ATAC we created and submitted 4 jobs at the UNIX based data cluster with 1 nodes and 8 ppn. Average completion time taken by each job was between 2–3 h, which includes trimming, alignment, sorting, removing duplicates, bam file generation, peak calling and merging replicates.

**Figure 6:**
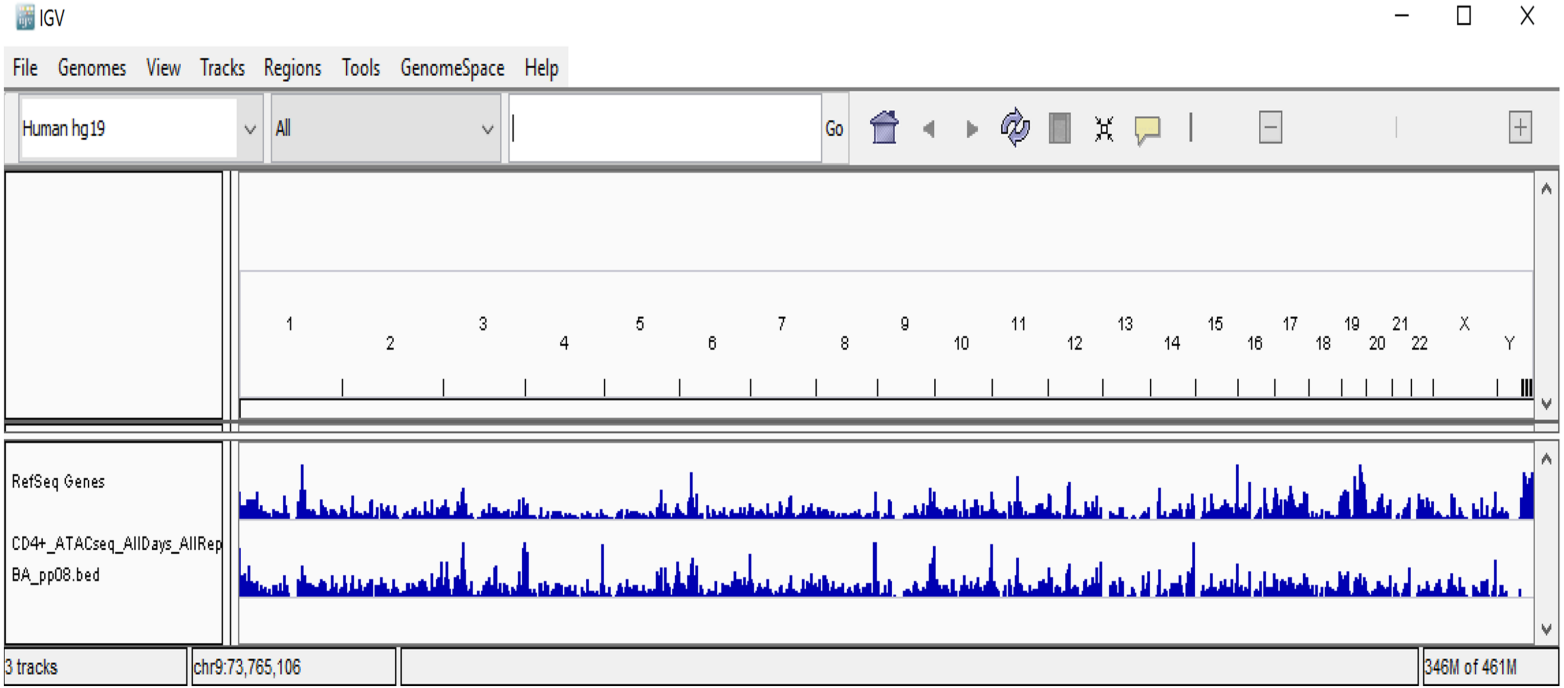
Visualization of CD4+T ATAC-seq samples. The figure presents the visualization of publically available CD4 +T ATAC-seq data. File “*CD4* +*_ATACseq_AllDays_AllReps_ZINBA_pp08.bed*” is using IGV.

**Table 2:** CD4+T ATAC-seq samples data download information. The table provides the information about publically available sources from where CD4+T ATAC-seq samples data (FASTQ, BAM and BED) files are downloaded.

No.	Name	File names and types	Web link
1	SRR891275	SRR891275_1.fastq.gz SRR891275_2.fastq.gz	http://www.ebi.ac.uk/ena/data/view/SRR891275
2	SRR891276	SRR891276_1.fastq.gz SRR891276_2.fastq.gz	http://www.ebi.ac.uk/ena/data/view/SRR891276
3	SRR891277	SRR891277_1.fastq.gz SRR891277_2.fastq.gz	http://www.ebi.ac.uk/ena/data/view/SRR891277
4	SRR891278	SRR891278_1.fastq.gz SRR891278_2.fastq.gz	http://www.ebi.ac.uk/ena/data/view/SRR891278
5	*CD4*+* ATACseq AllDays AllReps*	CD4+_ATACseq_All Days_AllReps_ZINBA_pp08.bed	https://www.ncbi.nlm.nih.gov/geo/query/acc.cgi?acc=GSE47753
6	*CD4*+* ATACseq AllDays AllReps*	CD4+_ATACseq.bam	https://www.ncbi.nlm.nih.gov/geo/query/acc.cgi?acc=GSE47753
7	*CD4*+* ATACseq AllDays AllReps*	CD4+_ATACseq.bam.bai	https://www.ncbi.nlm.nih.gov/geo/query/acc.cgi?acc=GSE47753

**Figure 7:**
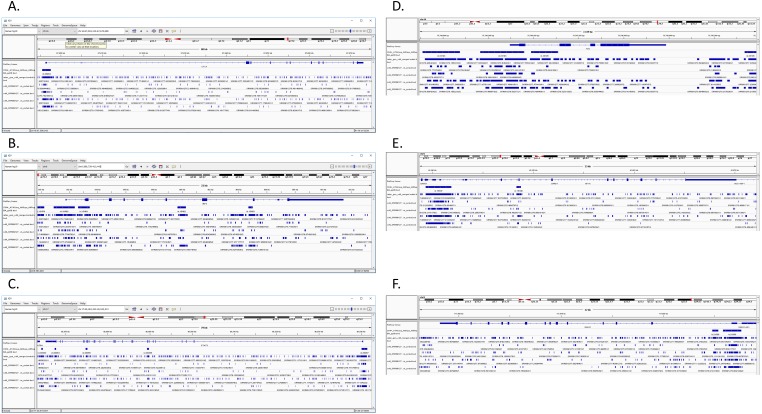
Comparison of merged BED of CD4+T samples and I-ATAC generated BED files. The figure presents comparative analysis based on genes CTCF (Fig. 7A), IRF4 (Fig. 7B), STAT3 (Fig. 7C), FOS (Fig. 7D), NFYA (Fig. 7E) and RAD21 (Fig. 7F), among downloaded merged BED file of CD4+T samples and I-ATAC generated individual sample’s generated BED file as well as the one with merged replicated.

**Figure 8:**
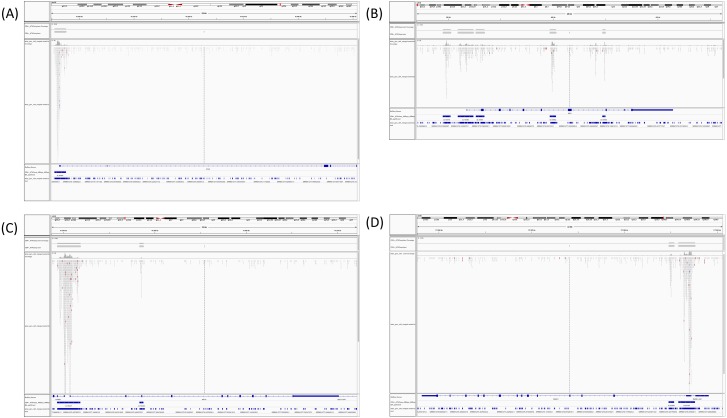
Coverage of merged BED of CD4+T samples and I-ATAC generated BED files. The figure presents, CTCF gene ID 36984 (Fig. 8A), IRF4 gene IDs 15479-82 (Fig. 8B), NFYA gene ID 16447 and 16448 (Fig. 8C), and RAD21 gene ID 23389 and 23390 (Fig. 8D) have coverage between publically available and our processed datasets.

We have used IGV for visual analysis of genes (CTCF, IRF4, STAT3, FOS, NFYA and RAD21) discussed in the published work (Buenrostro et al., 2013). Downloaded FASTQ files are Sanger / Illumina 1.9 encoded with sequence length of 50. We have presented selected statistical results (*Per base sequence quality, Per sequence quality scores, Sequence Duplication Levels, Per base sequence content, Per sequence GC content, Kmer Content*) produced by FASTQC (Fig. S1). All FASTQ file are variable in terms of their sequencing quality and length.

**Figure 9:**
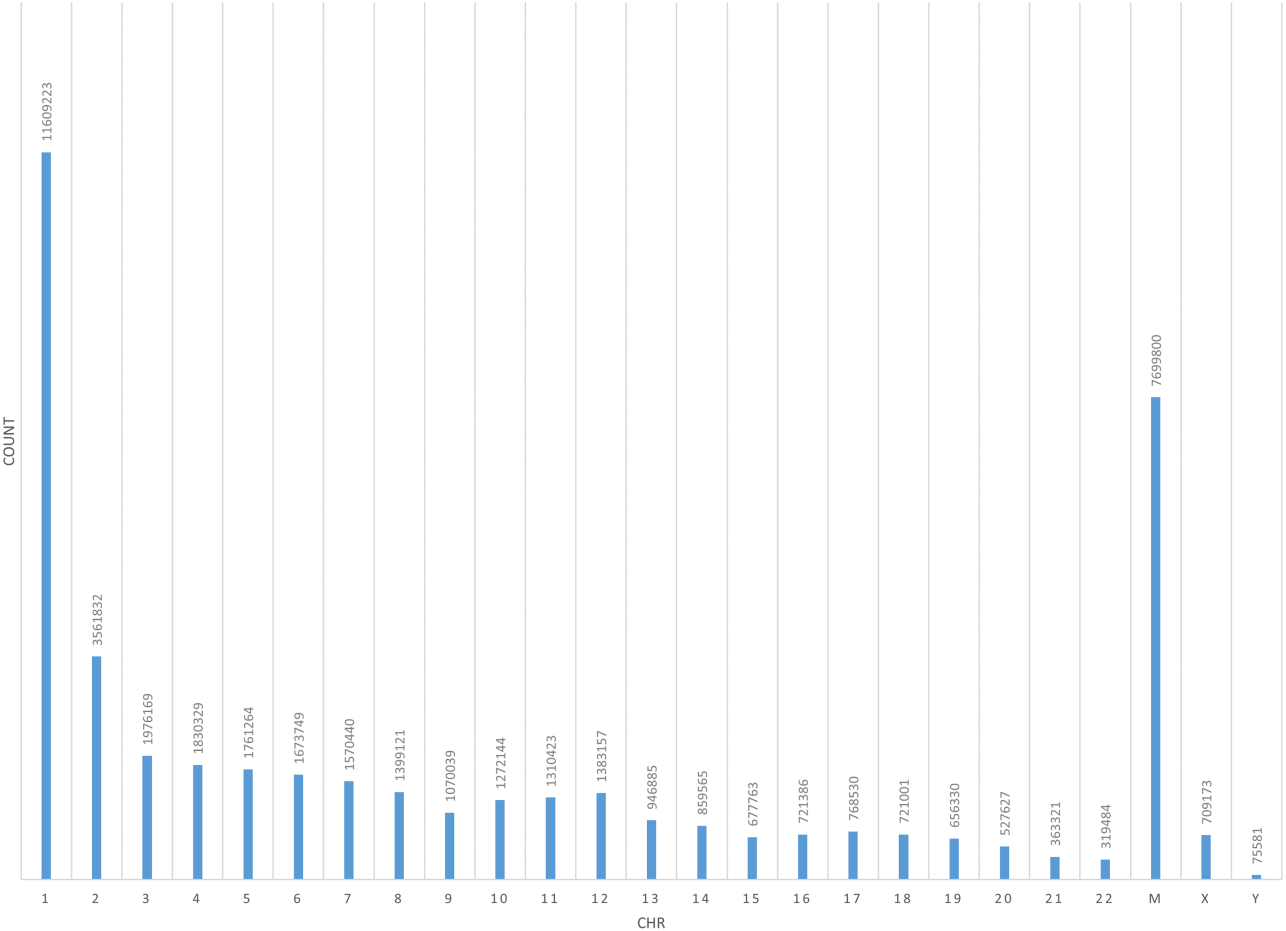
Coverage of all chromosomes of merged BED of CD4+T samples and I-ATAC generated BED files. The figure presents, total coverage count of chromosomes (1-22, M, X and Y) of merged BED of CD4+T samples and I-ATAC generated BED files.

We have also visualized and presented downloaded merged replicates of CD4+T samples, BED file (*CD4*+*_ATACseq_AllDays_AllReps_ZINBA_pp08.bed*) using IGV (Fig. 6) with reference human genome (hg19).

We have performed a brief comparative analysis among downloaded merged BED file of CD4+T samples (CD4+_ATACseq_AllDays_AllReps_ZINBA_pp08.bed) and I-ATAC individual sample’s generated BED file as well as the one with merged replicated. Figures 7A–7F presents comparison based on following genes: CTCF, IRF4, STAT3, FOS, NFYA and RAD21. Individual high resolution figures (Figs. 7A–7F) are in Supplemental Information 2.

Buenrostro et al. (2013) investigated ATAC-seq profile at the IL2 locus in context to the effect of distinct drugs on IL2 enhancers’ regulatory elements, found differential patterns for many genes e.g., CTCF, IRF4, STAT3, C-FOS, NFYA, and RAD21, etc. With a rational to identify a transcription factor pathway to block IL2 signaling, Buenrostro et al. found NFAT differentially regulated in T cell signaling. Further analysis revealed the total number of reads in “iatac_gen_cd4_merged.sorted.bam” are 34936720, whereas total number of reads in “CD4 +_ATACseq.bam” are 42749 (based upon only regions of interest). When computing the coverage of our merged bed file on the public bed file provided by Buenrostro et al. (2013), coverage depth for total number of bases covered by our merged file are 34936720. For determining the overlap between the two BED files, we took an intersect with the amount of overlap in base pairs per read. The total number of the overlapping reads were fund to be 5456252. Obtained results have overlapping reads from iatac_gen_cd4_merged.sorted.bam.bed followed by reads from CD4+_ATACseq_AllDays_AllReps_ZINBA_pp08.bed. The number of base pairs in the overlapped region are mentioned in the last column. In published data, CTCF gene ID 36984 (Fig. 8A), IRF4 gene IDs 15479-82 (Fig. 8B), NFYA gene ID 16447 and 16448 (Fig. 8C), and RAD21 gene ID 23389 and 23390 (Fig. 8D) have coverage (Fig. 9) between publically available and our processed datasets.

**Figure 10:**
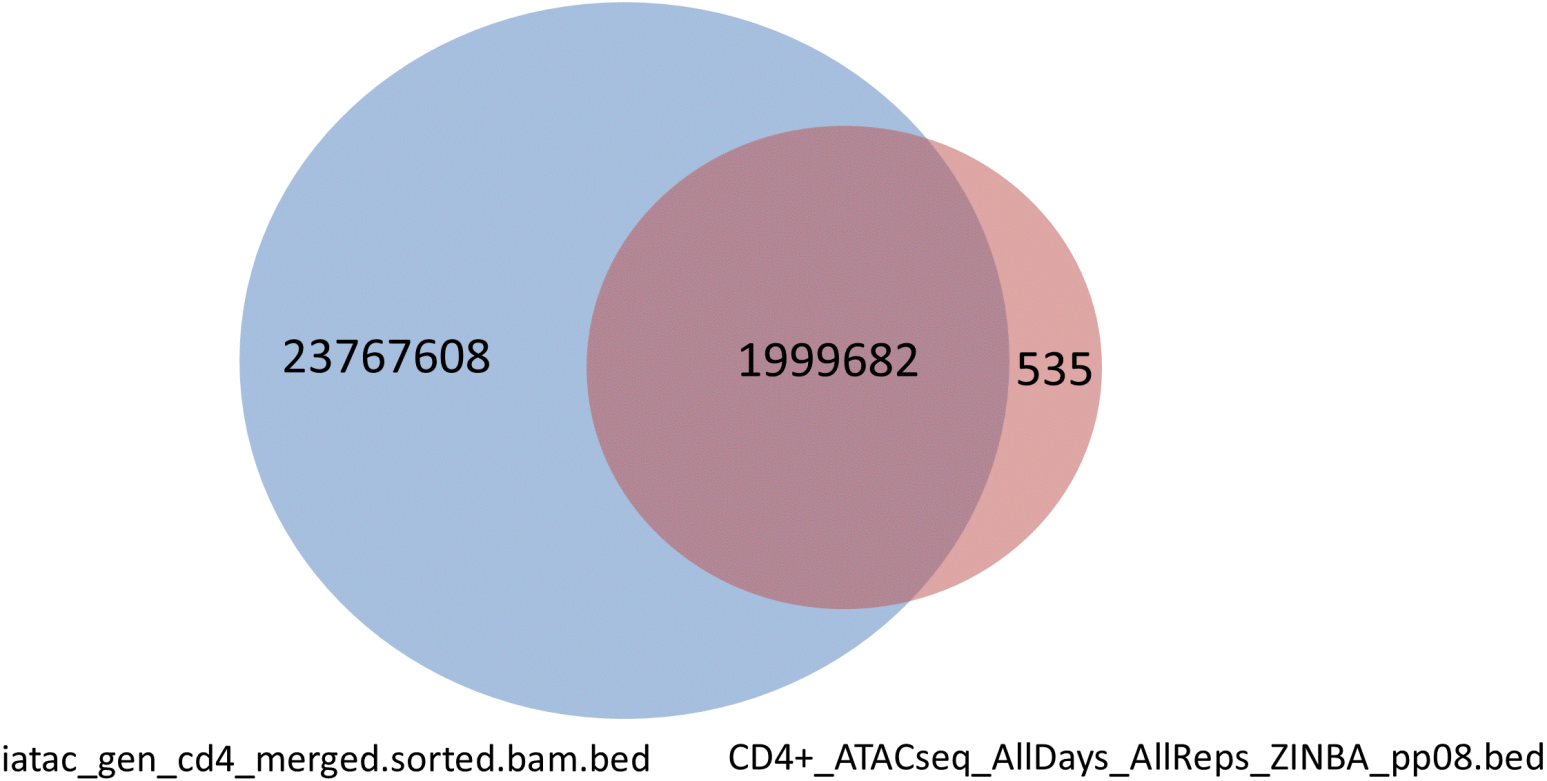
Intersected, 1999682 peaks with multiple overlap. The figure presents Venn diagram of overlapping peaks (1999682) from I-ATAC output (iatac_gen_cd4_merged.sorted.bam.bed) and the ZINBA generated output (CD4+_ATACseq_AllDays_AllReps_ZINBA_pp08.bed) by Buenrostro et al. (2013).

We also calculated and intersected to determine overlap peaks from the iatac_gen_cd4_merged.sorted.bam.bed (23767608 peaks) and CD4+_ATACseq_AllDays_AllReps_ZINBA_pp08.bed (42749 peaks). The total number of peaks was 25767290 and the total peaks with multiple overlap was 1999682 (Fig. 10).

This case study was performed using I-ATAC, which was run on Windows 10 platform and connected to the data cluster at the UConn Health Center. Further, features of I-ATAC platform are explained in detail with two example case studies in the Supplemental Information 1.

## Conclusion

One of the major requirements for the downstream analysis of any kind of genomics data (e.g., RNA-seq, ChIP-seq, ATAC-seq, etc.) is to first demultiplex and then pre-process FASTQ files using respective data pre-processing pipelines. The focus of this study is to develop an interactive, cross platform software for ATAC-seq data pre-processing. Many bioinformatics tools are open source and publicly available, which are helpful in compiling pipelines for ATAC-seq data pre-processing e.g., *ENCODE’s pipelines, Galaxy Biostar*, etc. However, these pipelines assume that the data analyst can operate with command-line tools, which is not always the case. Moreover, none of the available pipelines have a cross platform graphical user interface, which can be helpful in supporting non-computational scientists in tracking FASTQ files, loading default/customized settings, creating automatic directory structure, automatically generating shell scripts and submitting jobs to the attached data clusters, regardless of the number and kind (single or paired end) of input FASTQ files. To overcome these limitations in current command-line pipelines, we have developed a novel platform i.e., I-ATAC, that facilitates processing of ATAC-seq samples by non-computational scientists. We have successfully tested I-ATAC on ATAC-seq single and paired end data (in-house and publicly available) at The Jackson Laboratory for Genomics Medicine and UConn Health Center, USA. I-ATAC enables easy generation and tracking of output files including FASTQ files with high-quality reads (trimmed out sequence adapter and low quality reads) and sorted SAM, BAM and BED files. While I-ATAC has been implemented and well tested with ATAC-seq data, it can also be applied to perform quality checking and pre-preprocessing of WGS and ChIP-seq data.

##  Supplemental Information

10.7717/peerj.4040/supp-1 Supplemental Information 1 A step-by-step guide to I-ATAC, validating pipeline with two case studies

10.7717/peerj.4040/supp-2 Supplemental Information 2 Supplementary figures

## Additional Information and Declarations

### Data Availability

The following information was supplied regarding data availability: Github: https://github.com/UcarLab/I-ATAC.
