# Supplementary material for: I-ATAC: interactive pipeline for the management and pre-processing of ATAC-seq samples
Source: PeerJ. 2017 Nov 22;5:e4040. doi: 10.7717/peerj.4040 (PMC5702251; doi:10.7717/peerj.4040)
Supplement: Supplemental Information 1 [file peerj-05-4040-s001.pdf]

Supplementary Material: A step-by-step guide to I-ATAC, validating pipeline with two case studies

I-ATAC: Interactive pipeline for the management and pre-processing of ATAC-seq samples

Zeeshan Ahmed[1] and Duygu Ucar[2]

[1]University of Connecticut Health Center, 195 Farmington Ave, Farmington, CT, USA

[2]The Jackson Laboratory for Genomic Medicine, 10 Discovery Dr., Farmington, CT, USA

Corresponding authors: **Zeeshan Ahmed[1] and Duygu Ucar[2]**

Email address: **zahmed@uchc.edu** and **duygu.ucar@jax.org**

Software Availability: I-ATAC is programmed in Java and built at both Mac-OS-X and Windows platform.

- Its source code and executable are freely available at: https://github.com/UcarLab/I-ATAC
- Example dataset is available at: https://zenodo.org/record/46079#.WAe3l5MrK7Y
- Supporting software and dependencies are available at: https://zenodo.org/record/162023#.WAe3dJMrK7Y
- For additional information, please refer to the project webpage: https://www.jax.org/research-and-faculty/tools/i-atac

Table of Contents

1 Motivation 3

2 I-ATAC 4

3 Design Description 6

- **3.1 Operational Workflow of I-ATAC 6**
- **3.2 Applications integration, data processing pipeline and project's directory structure 7**
- **3.3 Comments workflow, operating systems and physical data storage in data cluster 9**

4 GUI Description 10

5 Integrated Applications Details 13

- **5.1 FASTQC: 13**
- **5.2 Trimmomatic 13**
- **5.3 BWA 13**
- **5.4 SAMtools 13**
- **5.5 Picard 14**
- **5.6 BEDtools 14**
- **5.7 ATAC_BAM_shifter_gappedAlign.pl 14**
- **5.8 MACS2 ..... 14**

6 Installation and Configuration 15

7 Case Studies 17

- **7.1 Example Dataset 17**
  - 7.1.1 Dataset Details 17
  - 7.1.2 Input 17
  - 7.1.3 Output 20
- **7.2 Case Study 2: Using GM12878 – CD4 T- Cells 23**
  - 7.2.1 Dataset Details 23
  - 7.2.2 Input 23
  - 7.2.3 Output 25

8 Conclusions 29

9 Acknowledgments 30

10 Funding: 30

11 Conflict of Interests: 30

12 Additional Requirements 30

13 References 30

1 Motivation

The use of high-throughput sequencing technologies has brought an enormous increase in the amount of heterogeneous genomic data production in the last decades. The importance of genomic dataset processing in the genomic community is well known; as it plays important role in analysing the dynamics and complexities of gene regulation with modelling and implementation of different statistical methods utilizing data processing pipelines.

Traditional way of next generation sequencing (NGS) data pre-processing is complex and based on running a series of command-line applications in Unix, Linux, MAC and DOS environments, which requires good knowledge of bioinformatics tools and good programming skills. There are over 200 tools available for the genome and exome sequencing data pre-processing and analysis (Pabinger *et al*., 2013) but most of them are non-interactive and command line based. Writing complex command line scripts and pipelines, and running non-interactive mode applications might be convenient for the scientists with good bioinformatics background but it is very hard for the biologist with no programming skills to conduct complex data analyses. The focus of our research is toward the application of a novel epigenomic profiling assay for transposase-accessible chromatin with high throughput sequencing (ATAC-seq) for integrative epigenomic analysis (Buenrostro *et al.*, 2013). ATAC-seq is a protocol to capture open chromatin sites (Buenrostro *et al.*, 2013; Buenrostro *et al.*, 2015a) by performing adaptor ligation and fragmentation of open chromatin regions (Tsompana and Buck, 2014).

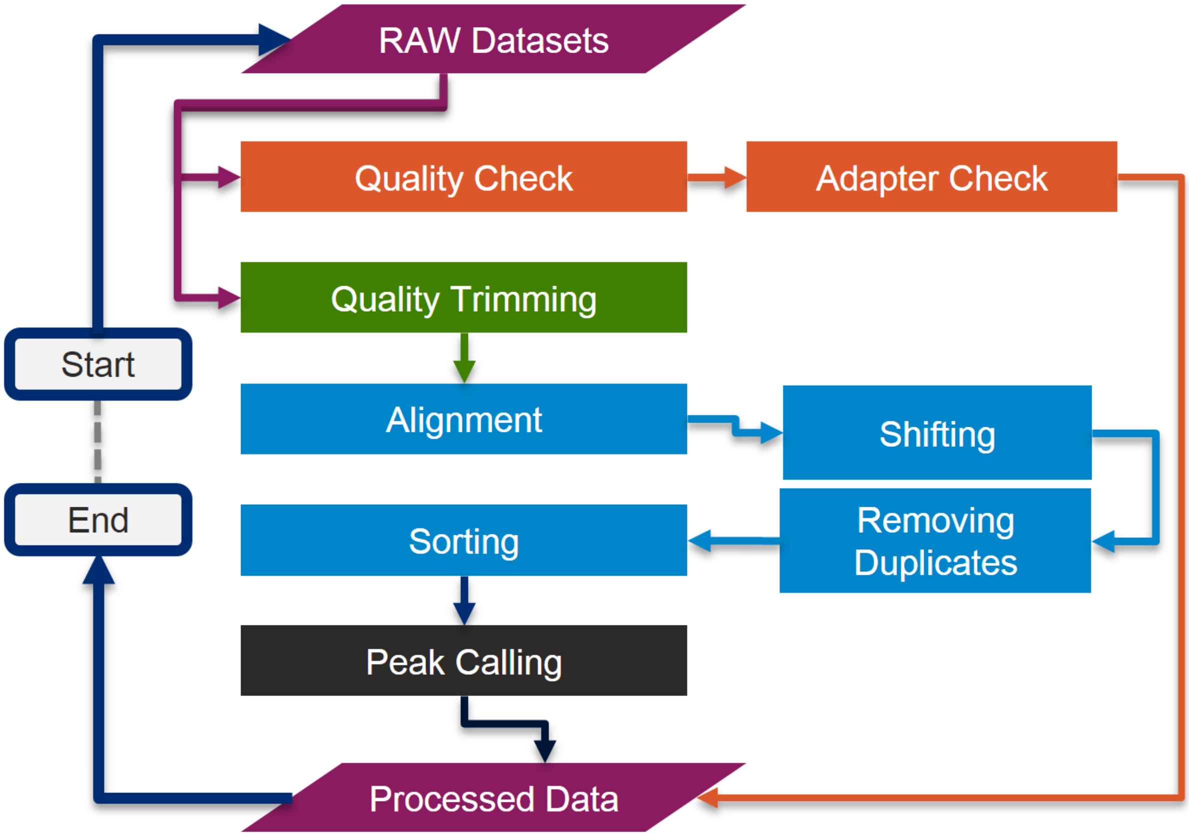

S-Fig. 1. ATAC-seq data pre-processing pipeline’s workflow.

ATAC-seq has been a popular chromatin profiling technology for clinical samples and has been used for the assessment of chromatin accessibility in various cells and tissues in human and model organisms e.g. (Moskowitz *et al*. 2017; Miskimen *et al.*, 2017; Bao *et al.* 2015; Buenrostro *et al.*, 2015b) etc. Due to its efficiency in requirement of biological sample and in library preparation time, many scientists are generating ATAC-seq libraries to decipher the chromatin landscape in a given cell type and condition of interest. To generate ATAC-seq libraries, a hyperactive molecule, Tn5 is used to cut the open chromatin and then short reads are sequenced typically from both ends (i.e., paired end). The next step is the processing of ATAC-seq samples. A typical ATAC-seq data processing pipeline's workflow is shown in S-Fig. 1, which starts with the quality check and adapter trimming, then alignment, shifting, removing duplicates, sorting and peak calling to find potential open chromatin sites, indicating active regulatory elements in each cell.

Processing and analysis of large number of ATAC-seq samples is a challenge for non-computational scientists since usually multiple tests are required to find the optimal algorithms and parameter settings. Interactive-ATAC (I-ATAC) (Ahmed and Ucar, 2017) is the first interactive, cross platform, user-friendly desktop application, which supports reproducible and automatic pre-processing of ATAC-seq (Buenrostro *et al*., 2013; Buenrostro *et al*., 2015) samples.

2 I-ATAC

The targeted end users of I-ATAC are mainly the biologists, who are familiar and comfortable with interactive operating systems (e.g. Windows, Mac-OS-X) and applications (e.g. web based browsers or client based viewers), yet have limited experience with programming, shell scripting, and with the Unix environment. Moreover, I-ATAC could be a helpful tool for bioinformaticians, who are new to the field of epigenomic data analysis and are not familiar with ATAC-seq data processing steps.

The GUI of the I-ATAC (S-Fig. 2 A and B) is designed for simplicity and ease by following human computer interaction (HCI) guidelines (Ahmed *et al.*, 2014). The concept behind designing I-ATAC GUI was to implement "One Click Operations" concept, similar to a Google search that requires users to enter one natural language based query and click a search button. Similarly, along with the default or customized settings (S-Fig. 2 B), I-ATAC requires only path to the sample data files (zipped or unzipped "FASTQ" files), project name and pressing button "Run ATAC-Seq" (S-Fig. 2 A) to perform following tasks:

- Get user login credentials
- Connect to the data cluster or local computer
- Create output directory structure
- Locate input data
- Copy & paste or create soft links of data to process
- Load modules, compilers & interpreters
- Write command line instructions to integrate applications
- Compose shell scripts (pipeline)
- Create & queue jobs (Unix based Secure Shell Scripts) at cluster or execute instructions on local computer
- Place output files in created directory structure
- Start data processing
- Disconnect to the connected data cluster

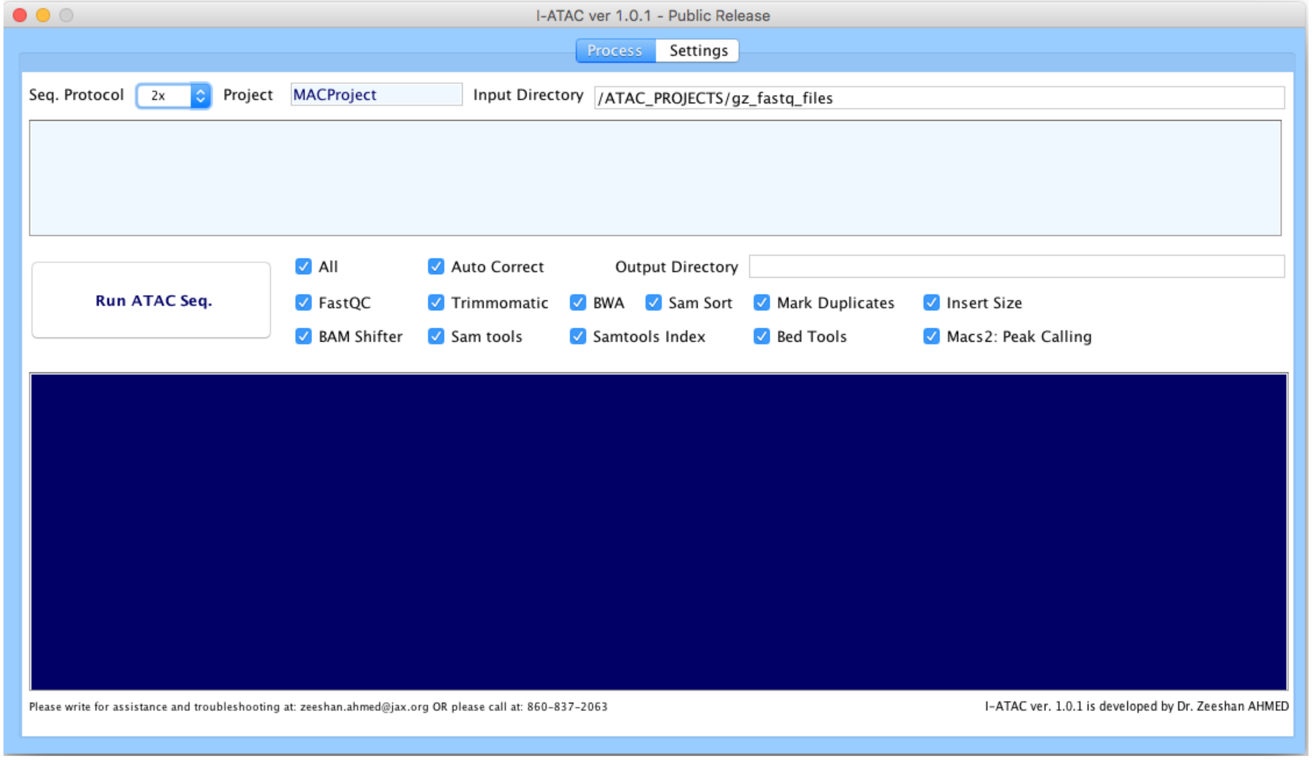

S-Fig. 2 (A): Graphical User Interface of I-ATAC: Create and run data processing jobs.

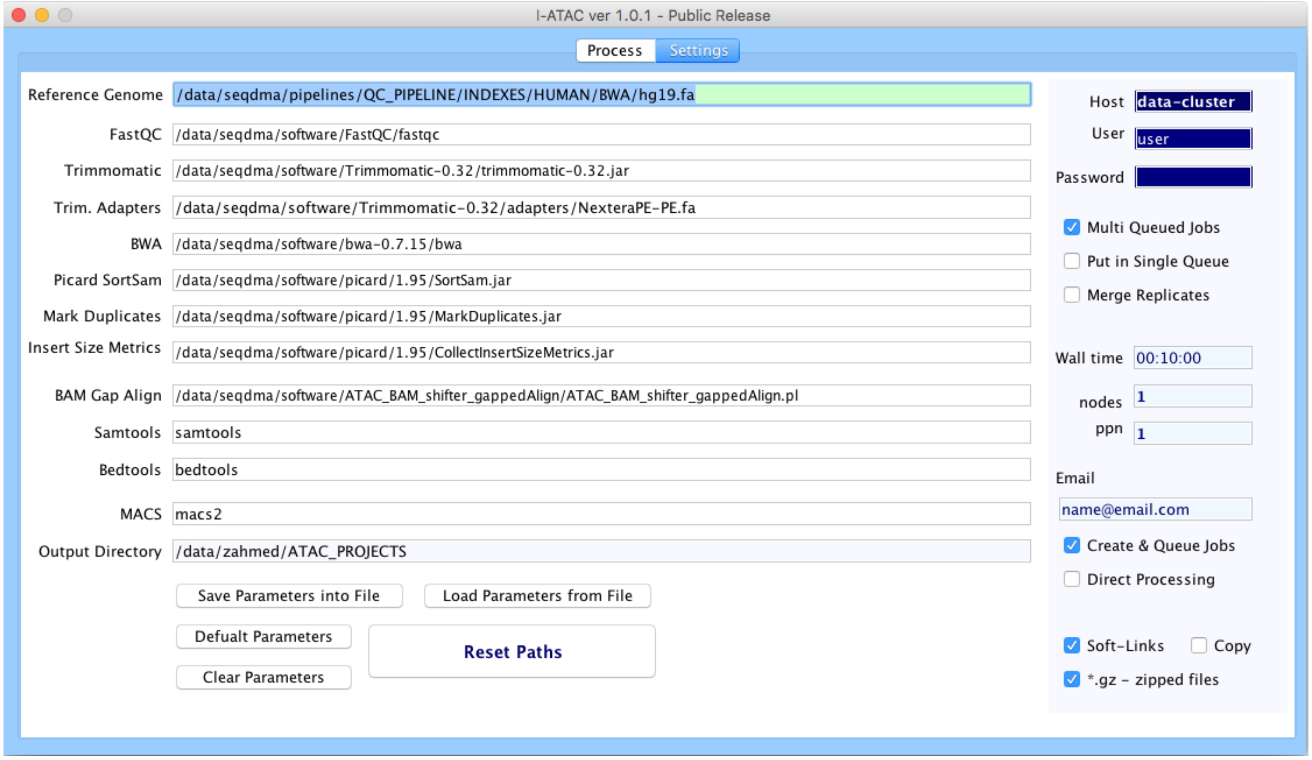

S-Fig. 2 (B): Graphical User Interface of I-ATAC: Set parameters and user credentials.

3 Design Description

I-ATAC is a platform designed by following software engineering principles for the sustainable bioinformatics software implementation (Ahmed *et al.*, 2014). Here, we present its operational workflow, data structure and components' orientation.

3.1 Operational Workflow of I-ATAC

Following default workflow (S-Fig. 3), user can process ATAC-seq samples with the application of complete pipeline, which involves the execution of all integrated applications *(FASTQC, Trimmomatic, BWA, Picard, ATAC_BAM_shiftrt_gappedAlign.pl, bedtools and macs2*) but user is not limited in the use of I-ATAC (S-Fig. 3). User can chose to run a single application as well as customize applications' workflow, following pre and post-requisites e.g. in case user is only interested in having FASTQC reports or trimming of low quality reads and adapters or user has already trimmed filtered FASTQ files but would like to map to reference genome only or may be only interested in generating BED files from BAM and peak calling etc. I-ATAC supported such customization and it can be very helpful, especially in trouble shooting situations, where due to any reason either pipeline could not fully execute or if there is already data exists in a form which does not require all steps of ATAC-seq pipeline. This customization can save time and computational resources.

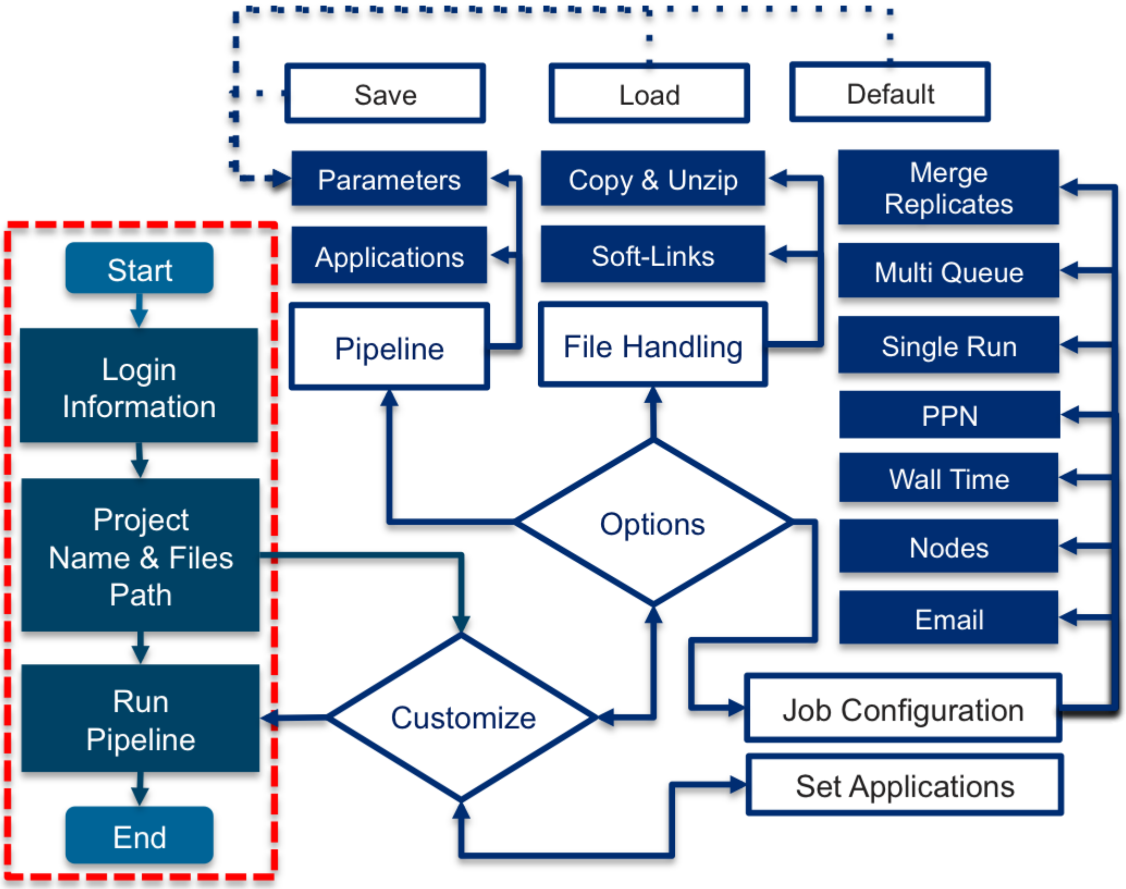

S-Fig. 3: I-ATAC: Operational workflow of I-ATAC

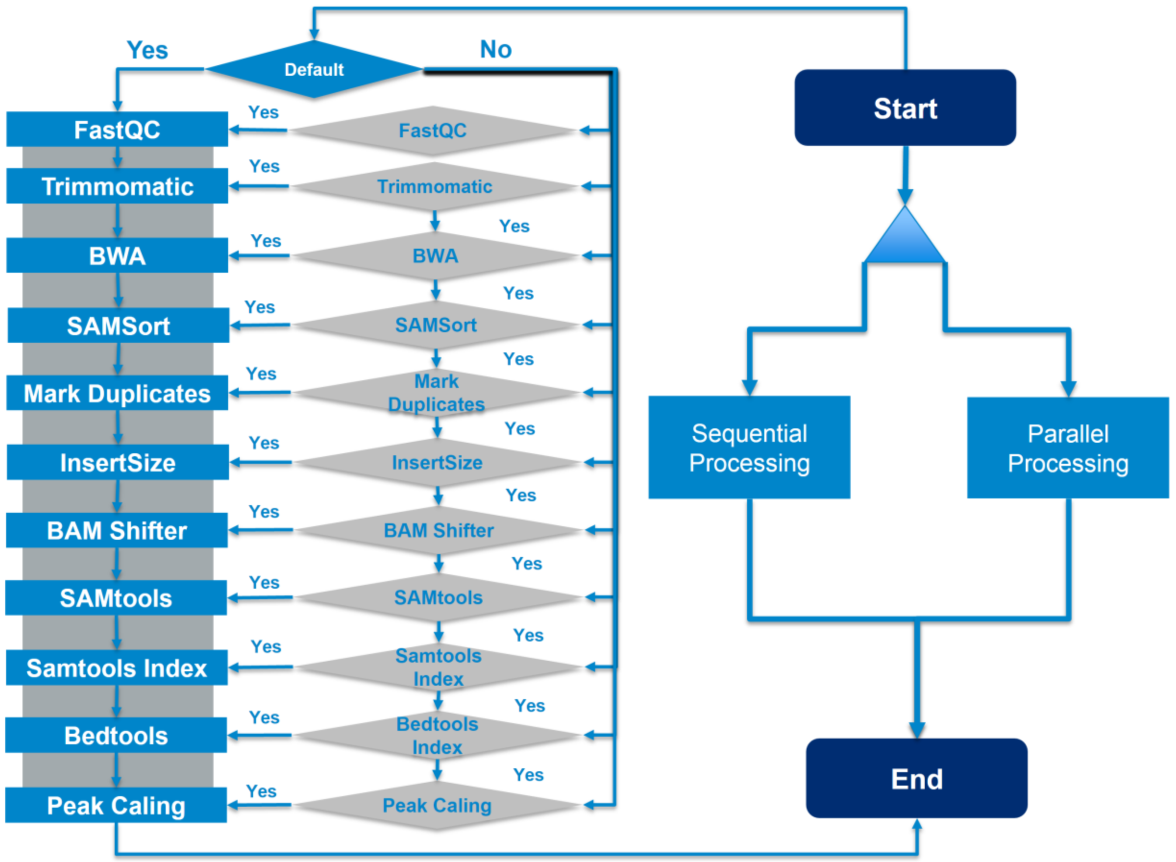

S-Fig. 4: I-ATAC: Customization of ATAC-seq data pre-processing pipeline with sequential (multiple jobs in one script) and parallel (multiple jobs in multiple scripts, one of each) processing.

User can remotely handle sample data files for processing by either keeping them in the same parent directory and putting only pre-processed results in the main project and sub-project directories or by first copying compressed files into the project directory, unzips them and then process them. User can configure job (UNIX based Secure Shell Scripts) settings by processing one or multiple samples at a time as one job or multiple jobs (one for each sample).

I-ATAC also enables users to customize parameters used for data pre-processing steps by letting the user to choose between applications as well as by setting different parameters (S-Fig. 4), which enables customizing this pipeline for the analyses of other data types, such as ChIP-seq data. As the output, I-ATAC produces data quality reports that can be visualized within the platform. It also outputs ATAC-seq reads that are filtered, trimmed and aligned as well as peak calls from these reads.

3.2 Applications integration, data processing pipeline and project's directory structure

ATAC-seq data processing pipeline starts with the quality check, then paired end reads are trimmed, aligned, filtered, and sorted in a "sam" file. The "sam" file is compressed and indexed to a bam file, which is then used as input for peak calling. To manage pre-processed data, proposed directory structure is followed and automatically created in data cluster before data processing (S-Fig. 5).

ATACseq_Projects
→ *Project_Name*
→ → *Sample_A_R1_Sample_A_R2*
→ → → *fastQC*
→ →→ *trimmomatic*
→ → →→ *bwa*
→ → → → →*macs2*
→ *mergedreplicated*
→ → *macs2*

All the quality reports (“zip” and “html” files) are placed in “fastQC” sub-directory. Compressed files contain different output files including text (“txt”) and web page (“html”). Text file contains information about basic statistics, file name, file type, encoding, total sequences, sequence flagged quality, sequence length, base number, mean, median, lower, quartile, upper, quartile, 10th percentile, 90th percentile, quality, Count, per base sequence content, per sequence GC content, per base N content, sequence length distribution, sequence duplication levels, overrepresented sequences, adapter content and Kmer content. Whereas html file visualize quantitative results.

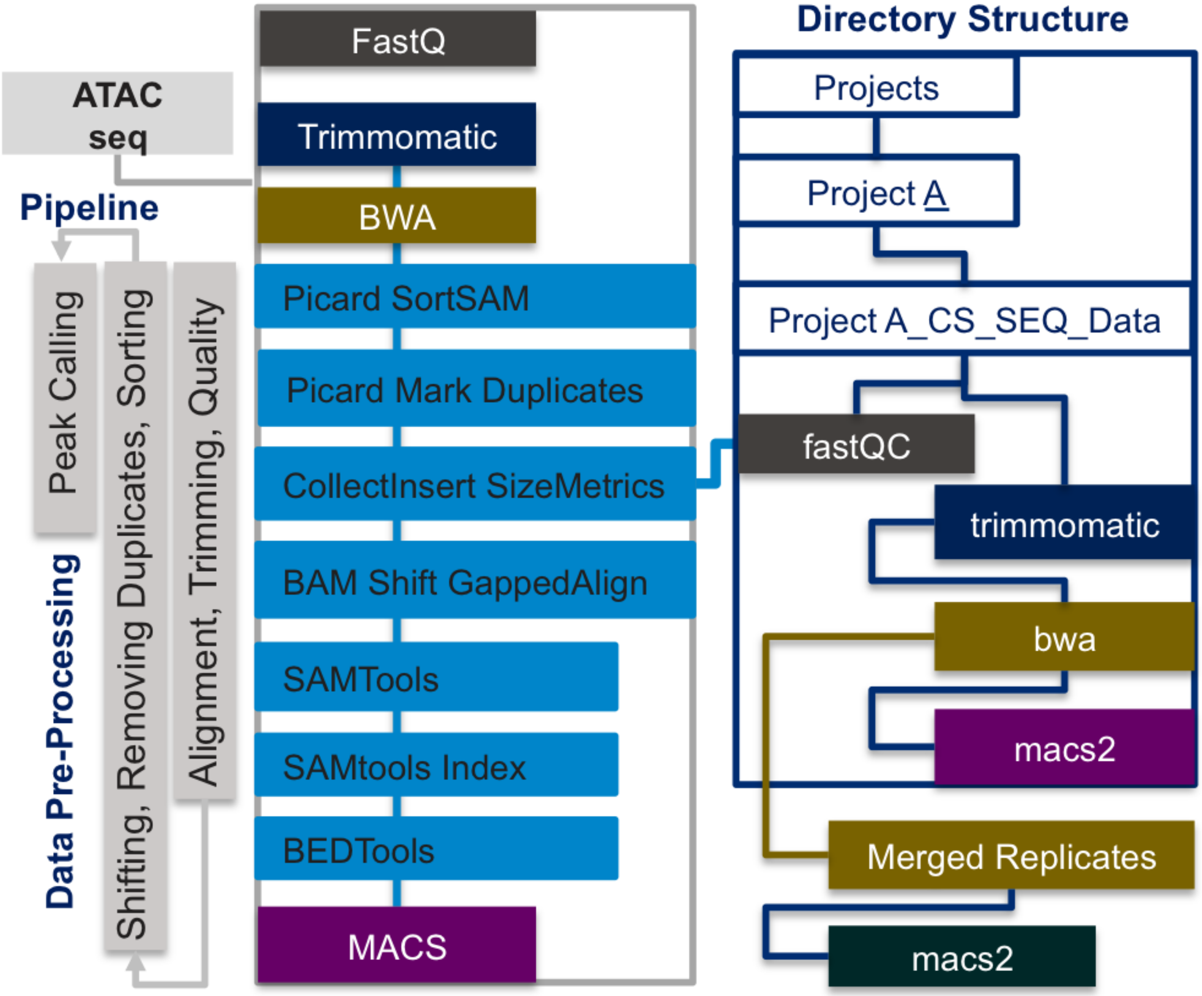

S-Fig. 5: I-ATAC: Applications and project directory structure.

All trimmed and filtered “FASTQ” files are placed in “trimmomatic" sub-directory, all the sorted, shifted “sam”, indexed “bam” and “bed” files are placed in “bwa” sub-directory. All the observed peak files are placed in the “macs2” sub-directory. The nested directory structure provides an organized and modular storage for multi-level ATAC-seq data analysis pipeline. Produced results in the form of sorted “sam” and “bam” files, as well as peaks can be visualized using available genome data browsers (e.g. USCS, Chipster etc.) and viewers (e.g. IGV etc.).

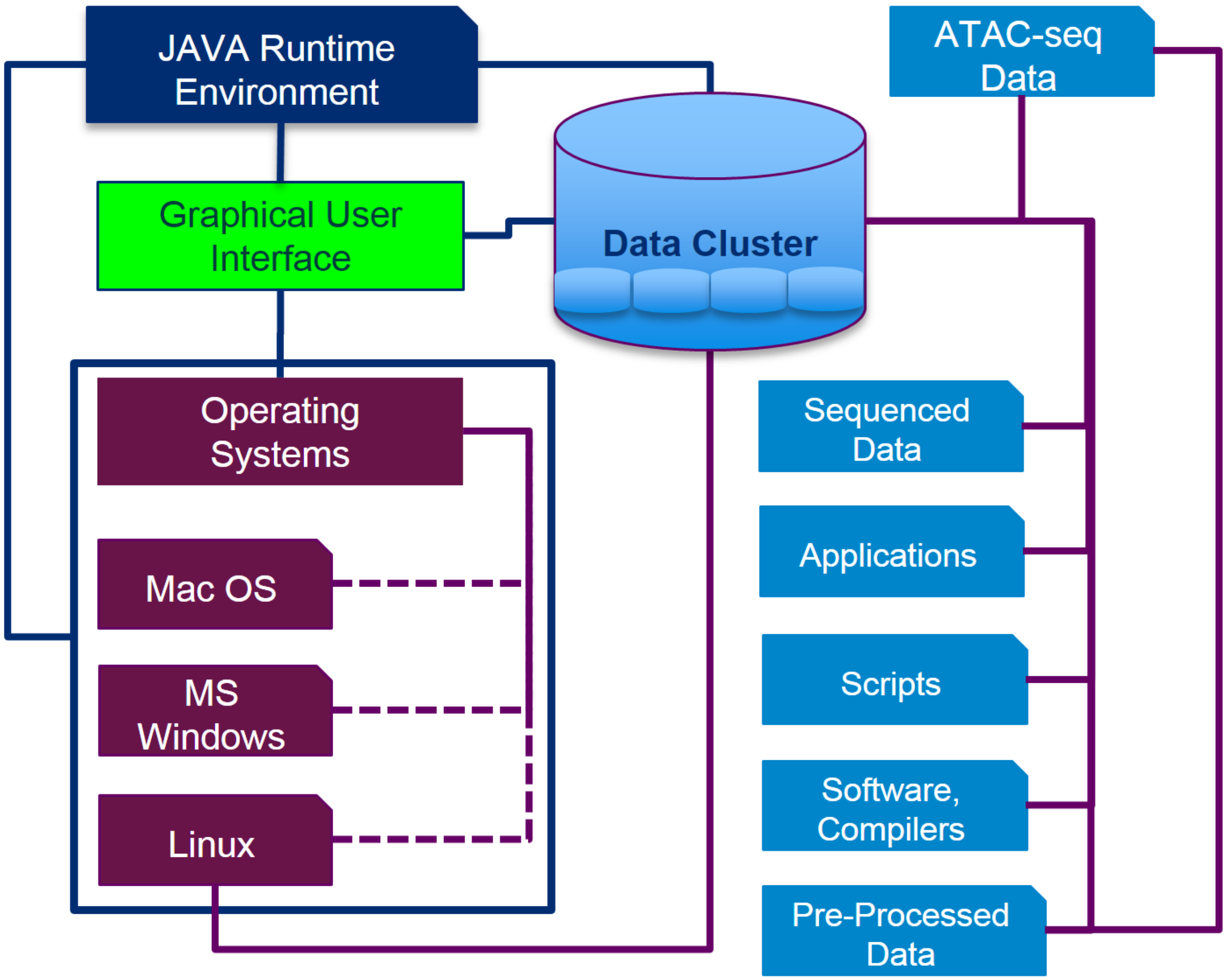

S-Fig. 6: I-ATAC: Components workflow, operating systems and physical data storage in data cluster.

3.3 Comments workflow, operating systems and physical data storage in data cluster

The components workflow (S-Fig. 6) of I-ATAC depends on the Java Run Time Environment (http://www.oracle.com/technetwork/java/javase/downloads/jre8-downloads-2133155.html), to be installed at in-use operation system, which can be Mac-OS-X, Microsoft Windows and Linux etc. The sample, sequenced data files, applications (S-Table. 3), compilers and interpreters (S-Table. 4), pre-processed data and scripts are need to be placed in data cluster.

4 GUI Description

As shown in (S-Fig. 2 A and B), the overall GUI of the I-ATAC is divided in to two modules: *Process* and *Settings*.

The *Process* module is to generate and run pipeline. *Process* provides six major features: *A1: Sequence protocol and Project Name, A2: Output directory, A3: Path to FASTQ file, A4: Start locating and processing data, A5: Customize pipeline, A6: File Status, and A7: Terminal status* (S-Fig. 7 and S-Table 1).

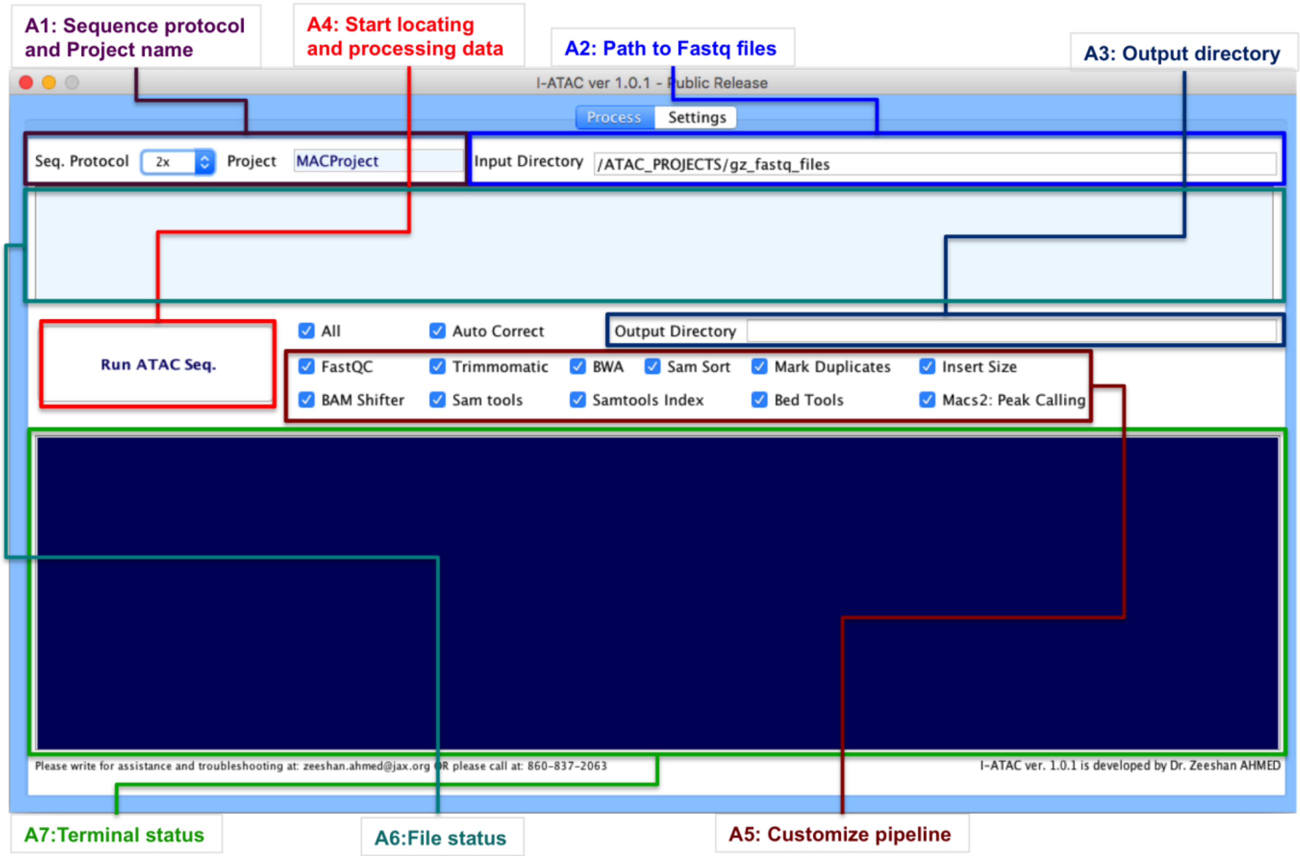

S-Fig. 7: GUI I-ATAC: Run ATAC-seq Pipeline (module: A).

Number	Feature	Description
A1	Sequence protocol and Project name	• Requires user to select either "1x" or "2x" for single and paired end data.<br>• Requires user to enter a Project Name, without any spaces and special characters. Reason to avoid spaces and special characters is, that, I-ATAC will automatically create a new directory, where data will be referenced (soft links) or copied for processing and results will be placed.
A2	Path to the FASTQ files	• Requires path to the directory, where data (FASTQ files) are placed.
A3	Output directory	• Requires path to the directory, where processed data (outcome/output files) will be placed.
A4	Start locating and processing data	• Starts locating sample data files, copy from source to the main destination (project) director, unzip compressed sample data files,

		automatically generate script, queue script(s) as job(s), perform data processing and place output in proposed directory structure.
A5	Customize Pipeline	• Default parameters include I/O redirected, sequential combination of integrated applications and parameters. Twelve different options are integrated:<br>**1. FASTQC**<br>**2. Trimmomatic**<br>**3. BWA**<br>**4. Sam Sort**<br>**5. Mark Duplicates**<br>**6. Insert Size**<br>**7. BAM Shifter**<br>**8. SAM tools**<br>**9. SAM tools index**<br>**10. BED tools**<br>**11. MACS2**<br>**12. All**<br>**13. Auto Correct**<br>• Option 12 is to select all options and perform data processing with default settings and option 13 is to correct the sequence I/O.
A6	File Status	• Provides information about located data samples in the data cluster, using provided input path.
A7	Terminal Status	• Provides information about execution of job in data cluster.

S-Table. 1: Features description of GUI-A: Run ATAC-seq Pipeline

The GUI-B module is mainly used to set the parameters of the applications and directory paths. As shown in the figure (S-Fig. 8), it provides only four features: *Applications Parameters*, *Directory Paths, Save and load Parameters*, and *Reset Paths* (S-Fig. 8 and S-Table 2).

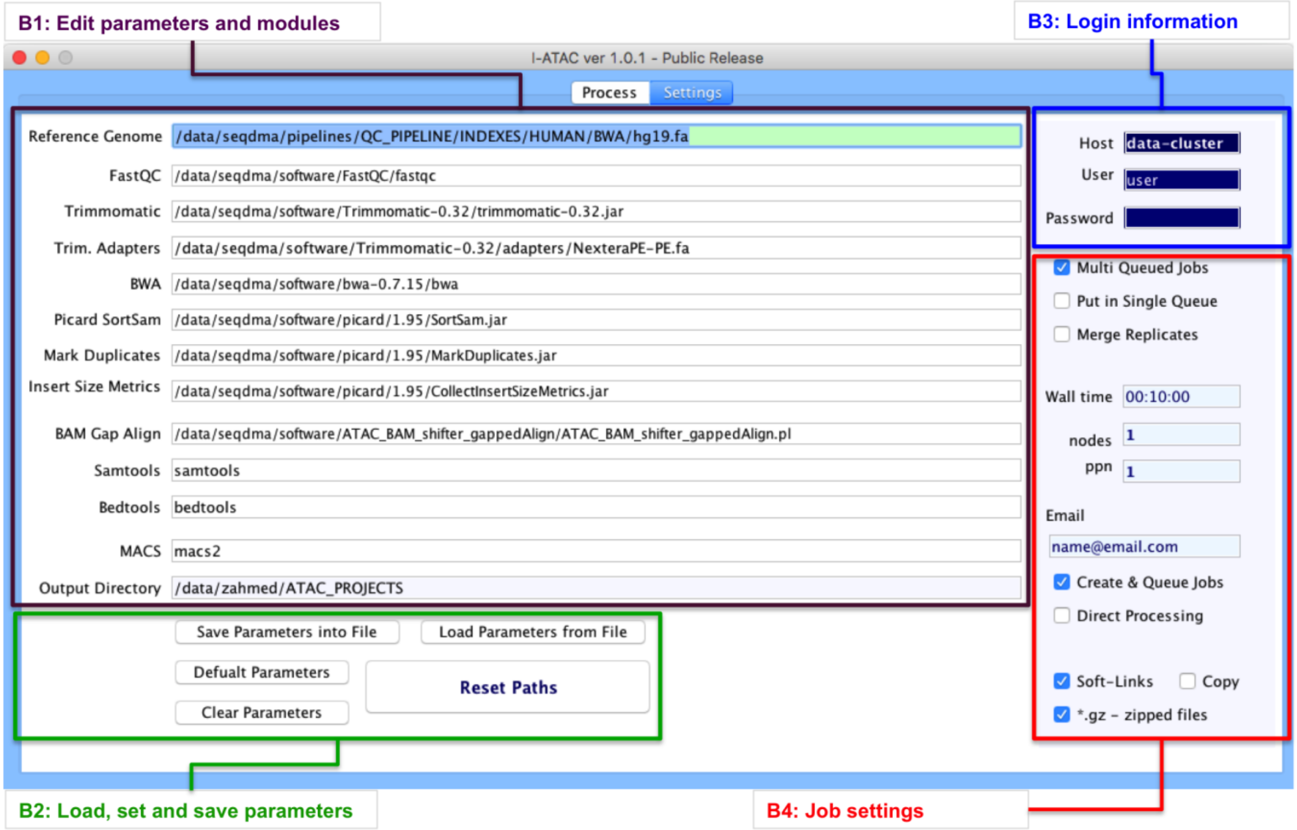

S-Fig. 8: GUI I-ATAC: Set Script Parameters (module: B).

Number	Feature	Description
B1	Applications Parameters	• Paths and calling instructions to the following integrated applications and referenced genome and adapters:<br>**1. Reference genome**<br>**2. FASTQC**<br>**3. Trimmomatic**<br>**4. Adapters**<br>**5. BWA**<br>**6. SamSort**<br>**7. Mark Duplicates**<br>**8. Insert Size Metrics**<br>**9. BAM Gap Align**<br>**10. SAM tools**<br>**11. BED tools**<br>**12. Samtools Index**<br>**13. MACS2**<br>**14. Output Directory**
B2	Load, set and save parameters	Five options (buttons) are provided:<br>**1. Save Parameters:** To save the set parameters in the form of a text file, to reuse and share settings.<br>**2. Load Parameters:** To load saved settings in the form of a text file.<br>**3. Clear Parameters:** To clear all parameter fields.<br>**4. Default Parameters:** To load default parameters.<br>**5. Reset Paths:** To apply modifications.
B3	User Login	• User requires entering name of the host (attached data cluster or name of the personal computer), user login name and password to let the I-ATAC successfully login into to host and access sample data files (“FASTQ”) and applications to perform data processing.
B4	Job Settings	Default job (set of instructions, written in the form of a script and executed like a program (executable software) to perform certain set of operations) related parameters. Furthermore I-ATAC provides eight different options to customize script generation and job submission:<br>**1. Multi Queued Job**: Processes multiple samples at a time by generating and submitting parallel-multiple data processing jobs (one for each).<br>**2. Put in Single Queue**: Processes one or multiple samples at a time b generating and submitting one data processing job (one for all.<br>**3. Merge Replicates**: Applicable only in case of processing multiple samples at a time by submitting one data processing job for all. It enables selection of all generated “bam” files from all the pre-processed samples directories (bwa) and performs peak calling.<br>**4. Wall Time**: Sets time to be allocated for the processing of the queued job. In case of multiple-parallel jobs, it will set provided time for all jobs.<br>**5. Nodes**: Sets the number of nodes (connection points) requested for job. Default set node is 1.<br>**6. Processor per node (ppn)**: Sets the number of cores (virtual processors) per node per. Default set ppn is 1.<br>**7. Email**: Sets to get notification (cancelled, completed) about the status of submitted job.<br>**8. Create & Queue Jobs**: In case host is data cluster, then I-ATAC will prepare and submit jobs.<br>**9. Direct Processing**: In case host is personal computer, then I-ATAC will prepare and submit instructions.<br>**10. Creates soft links**: Having checked this option, I-ATAC will create soft links of FASTQ files in to output directory.<br>**11. Copy**: Having checked this option, I-ATAC will create copy FASTQ files in to output directory.<br>**12. *.gz ziiped files**: Having checked this option, I-ATAC will expect input FASTQ files are zipped otherwise not.

S-Table. 2: Features description of GUI-A: Set Script Parameters.

The sole objective of developing I-ATAC is to help with the provision of interactive ATAC-seq data processing pipeline that is why; we have not developed features for file handling between data cluster and operating systems. There are already some interactive tools available for such purposes e.g. File Zilla (https://filezilla-project.org), WinSCP (http://winscp.net/eng/download.php), Cyberduck (https://cyberduck.io/?l=en) etc.

5 Integrated Applications Details

ATAC-seq data processing pipeline consists of different third party applications (S-Table. 4); I/O (input/output) redirected (one's output is treated as another's input, in terms of both data analysis and processing) and integrated method (S-Fig. 6). Additionally, it requires all needed compilers and interpreters to be downloaded and installed as well (S-Table. 4).

5.1 FASTQC:

It is a command line based, non-interactive tool for the high throughput sequence data. It is programmed in Java and requires Java Runtime Environment and Picard BAM/SAM libraries to be installed in the data cluster. Its output is based on Basic Statistics, Per base sequence quality, Per tile sequence quality, Per sequence quality scores, Per base sequence content, Per sequence GC content, Per base N content, Sequence Length Distribution, Sequence Duplication Levels, Overrepresented sequences, Adapter Content and Kmer Content. FASTQC used version details, including input, output and download details are given in S-Table. 3, Row No.: 1.

5.2 Trimmomatic

It is a command line based, non-interactive tool for the trimming of reads (Bolger *et al*., 2014) using paired-end and single ended data produced by the Illumina next generation sequencing technology (http://www.illumina.com/). It takes compressed or uncompressed FASTQ (phred-33 and phred-64 quality scores) file as input andmainly performs adapter filtering, sliding window trimming, base cutting (start and end of reads, as well, at specific number) and removes below quality reads. Trimmomatic's used version details, including input, output and download details are given in S-Table. 3, Row No.: 2.

5.3 BWA

Burrows-Wheeler Alignment tool (BWA) is a software application for aligning short nucleotide sequences to a reference genome (Li and Durbin, 2009). It implements BWA-backtrack for reading sequence up to 100bp, and BWA-SW and BWA-MEM algorithms are for reading longer sequences between 70bp to 1Mbp. BWA's used version details, including input, output and download details are given in S-Table. 3, Row No.: 3.

5.4 SAMtools

Sequence Alignment/Map (SAM) tools is a software package with various utilities, mainly used for sequence data formatting (Li, 2011; Li, *et al*., 2009). It helps in performing complex operations at sequence data files, including variant calling, alignment, sorting, indexing, viewing, data extraction and format conversion. SAMtools applied package's version details, including input, output and download details are given in S-Table. 3, Row No.: 4.

5.5 Picard

It is Java based non-interactive tool, which requires Java Runtime Environment to execute. It is mainly used for the sequence data manipulation in sam and bam files. Both sam and bam files contain same data structure and format, sam is human readable, whereas, bam is machine-readable format (binary). It's used version's details, including input, output and download details are given in S-Table. 3. It performs sorting in order and can read information about library, platform, sample, sequence, predicted insert size etc. Picard's used version details, including input, output and download details are given in S-Table. 3, Row No.: 5.

5.6 BEDtools

Browser Extensible Data (BED) tools (Quinlan and Hall, 2010) is a software application for converting "bam" to "bed" files and compare large sets of genomic features. Moreover, it can be used for converting BEDPE intervals to BAM and BAM to FASTQ, finding closest and potentially non-overlapping interval, creating HTML pages to link UCSC locations, finding pairs that overlap other pairs and intervals in various ways, randomly redistributed and adjust size of intervals and tag bam alignment etc. BEDtools used version details, including input, output and download details are given in S-Table. 3, Row No.: 6.

5.7 ATAC_BAM_shifter_gappedAlign.pl

ATAC_BAM_shifter_gappedAlign.pl is an open source Perl script, which can be used to perform read shifting based on the read quality. It takes aligned "bam" file as an input and offsets by 4bp for the positive strand (sequence containing instructions for building a protein) and –5bp for the negative strand (merely contains the complementary sequence and according to the base-pairing rules it is not normally transcribed into RNA nor translated into protein). Users can use any other tools for shifting the reads. ATAC_BAM_shifter_gappedAlign version details, including input, output and download details are given in S-Table. 3, Row No.: 7.

5.8 MACS2

Model-based Analysis of ChIP-Seq (MACS) (Zhang, *et al*., 2008) is a tool for analyzing short reads for the spatial resolution of the predicted sites, capturing local biases in the genome and generation of peaks with detailed information about length, genome coordinates, summit, p-value, q-values, false-discovery rate (FDR) and fold enrichment. MACS2's used version details, including input, output and download details are given in S-Table. 3, Row No.: 8.

No.	Applications	Versions	Download Web links	Input File Formats	Outputs File Formats
1	FASTQC	0.11.2	http://www.bioinformatics.babraham.ac.uk/projects/fastqc/	FASTQ	html, zip, txt.
2	Trimmomatic (Bolger *et al.*, 2014)	0.32	http://www.usadellab.org/cms/?page=trimmomatic	FASTQ	FASTQ, trimU.fastq,
3	Burrows-Wheeler Alignment tool (BWA) (Li and Durbin, 2009)	0.7.10	http://bio-bwa.sourceforge.net	fastq_filtered	sam
4	Sequence Alignment/Map (SAM) tools (Li, 2011; Li, *et al.*, 2009)	0.1.19	http://samtools.sourceforge.net	sam	Sam. txt, pdf
5	Picard	1.95	http://broadinstitute.github.i	sam, bam	sam, bam

			o/picard/		
6	Browser Extensible Data (BED) tools (Quinlan and Hall, 2010)	2.22.0	http://bedtools.readthedocs.org/en/latest/content/overview.html	bam	bed
7	ATAC_BAM_shifter_gappedAlign	1	https://github.com/acdaugherty/scripts/blob/master/MostUsed/ATAC_BAM_add1.pl	bam	bam
8	Model-based Analysis of ChIP-Seq (MACS) (Zhang, *et al.*, 2008)	2.1.0.20151222	http://liulab.dfci.harvard.edu/MACS/	bed	bed, bdg, broadPeak, gappedPeak

S-Table. 3: Integrated applications in I-ATAC data processing pipeline

No.	Compiler / Interpreter	Versions	Download Web links
1	JAVA	8	https://www.java.com/en/download/
2	R	3.2.3	https://www.r-project.org
3	Perl	5.10.1	https://www.perl.org
4	Python	2.7.3	https://www.python.org

S-Table. 4: Needed compilers and interpreters

6 Installation and Configuration

The software executable (JAR file) is open source and freely available and to execute I-ATAC, major requirement is the installation of Java Runtime Environment (http://www.oracle.com/technetwork/java/javase/downloads/jre8-downloads-2133155.html).

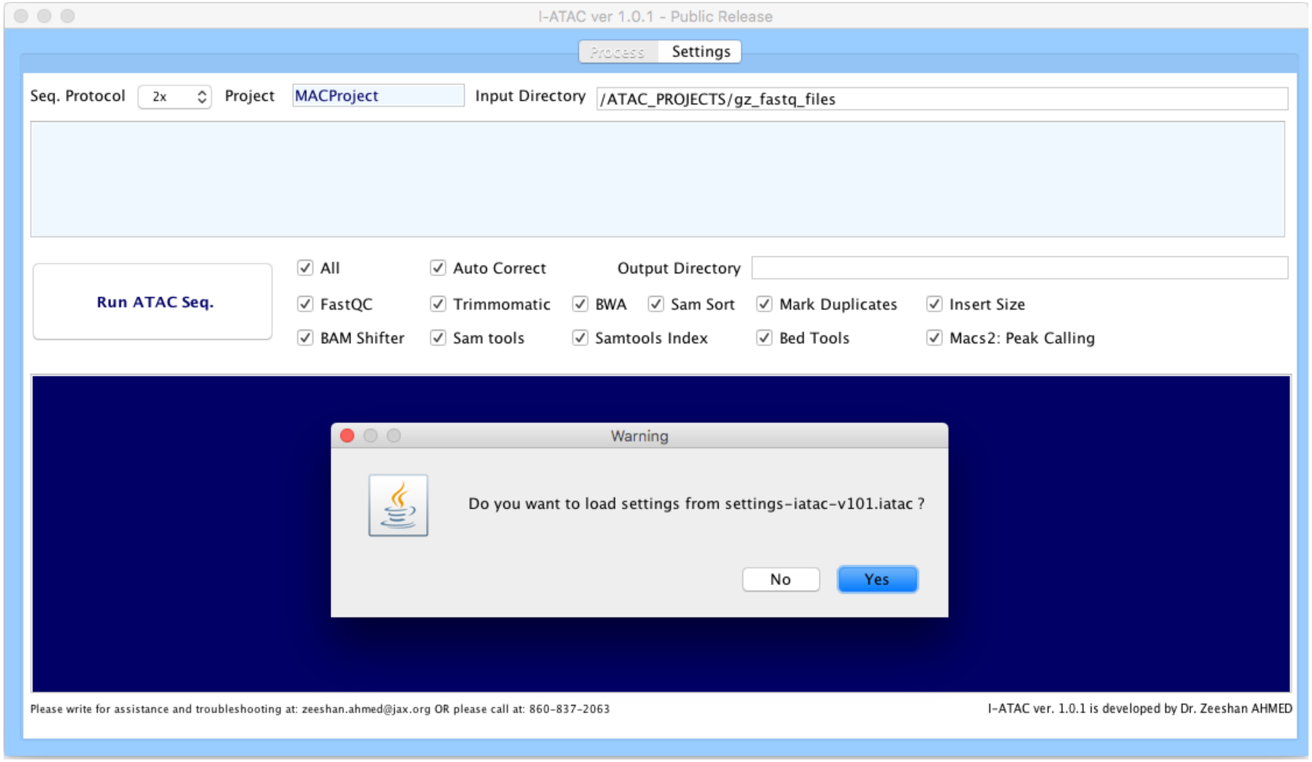

S-Fig. 9A: I-ATAC, parameters and protocol settings.

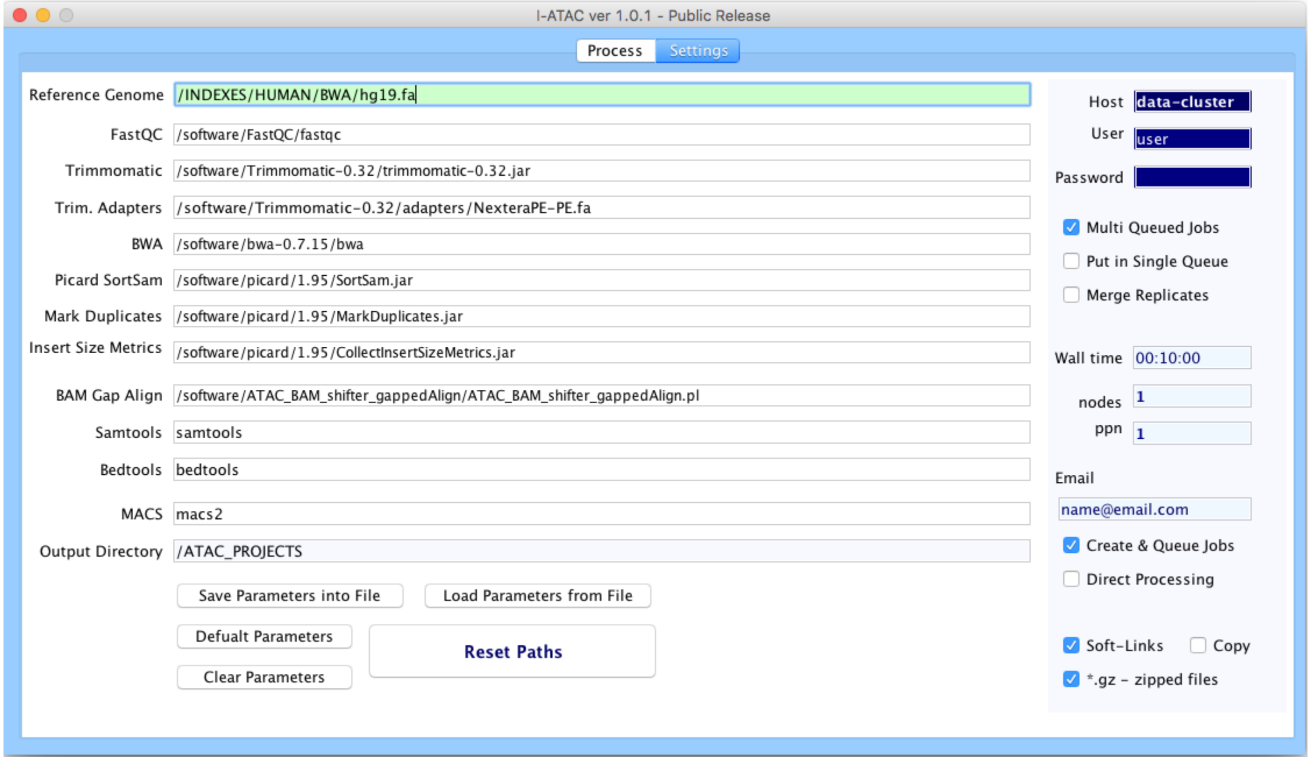

S-Fig. 9B: I-ATAC, default setting.

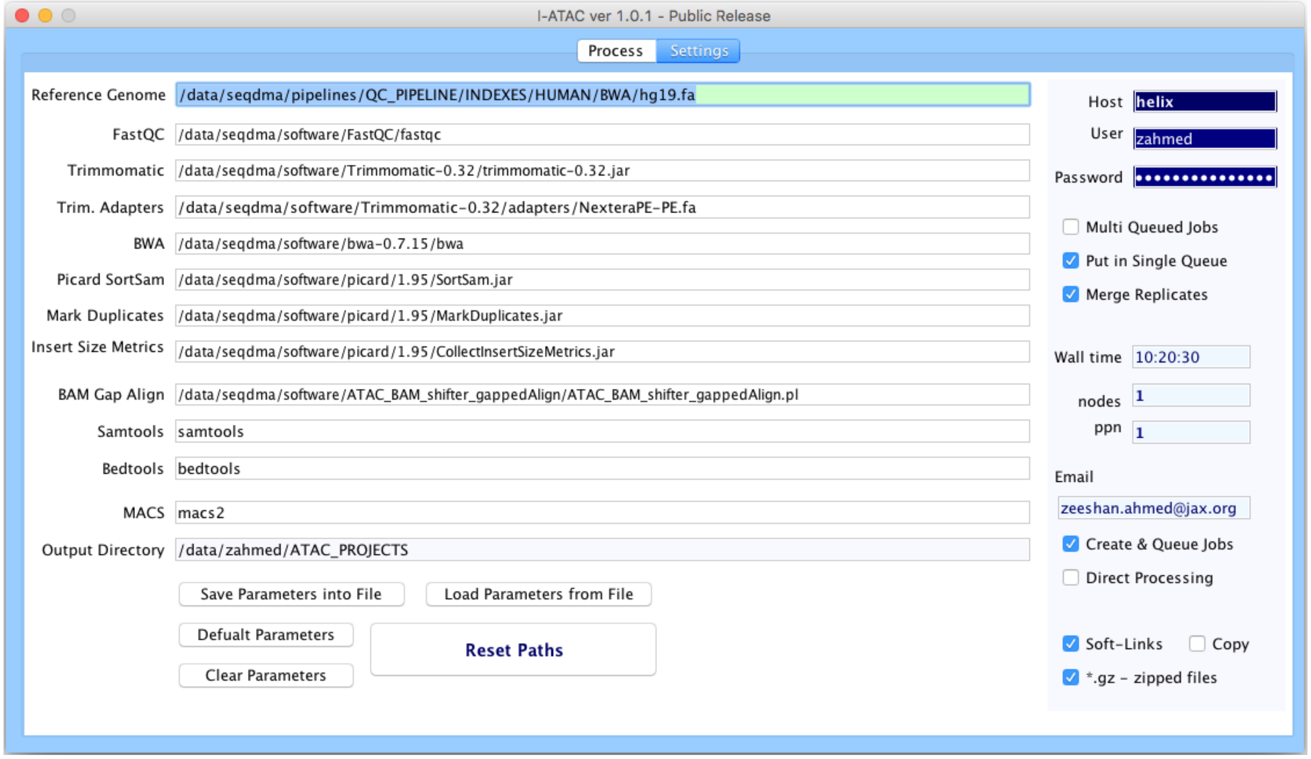

S-Fig. 9C: I-ATAC, user setting, loaded from “settings-iatac-v101.iatac”.

After executing I-ATAC and before starting data processing, it is important to set valid applications paths and calling protocols (section: Graphical User Interface of I-ATAC). Our default parameters (S-Fig. 9A, 9B and 9C) are set according to our data cluster and installed versions of application (S-Table. 3), and Compilers/Interpreters (S-Table. 4).

Using default configuration settings; I-ATAC will consider logged-in user with a default directory of same name as of user in the data cluster (e.g. Zeehan → "d:/data/Zeeshan/ATAC_PROJECTS/"). However, user can alter, reset and save default project directory settings.

7 Case Studies

In order to validate the performance of I-ATAC and to guide the users, we present two case studies. First involves using the example data; where we have created small size example dataset (provided in supplementary material and can be downloaded from the following web link: https://zenodo.org/record/46079#.VsJMg7S5LHM) with artificial names (to explain the process, execution steps in simpler way.). The reason for giving example study is to let the user, use the application and observe results in possible shortest time. Moreover, it will also help in figuring out and resolving trouble shooting conditions (e.g. could be due to inappropriate installation of downloaded application and compilers/interpreter or any other exceptional reason etc.). Second study is using publically available data (GM12878, CD4); where we have processed publically available data, which a trained user can download and process using I-ATAC. In both case studies, I-ATAC is run at the Mac-OS-X-Yosemite 10.10.5 platform.

7.1 Example Dataset

7.1.1 Dataset Details

Raw dataset and produced results mentioned in this example case study, which can be downloaded from the provided project web link. Sequenced, paired sample data ("FASTQ" or "FASTQ.gz") files are need to be collected and placed in the attached data cluster.

7.1.2 Input

The input to I-ATAC is the path to ATAC-seq sample data, which in our case is:

"/data/zahmed/ATAC_PROJECTS/gz_fastq_files"

As shown in S-Fig. 10, there are two samples available (paired data, four "FASTQ" zipped files) in the above-mentioned directory i.e. "gz_fastq_files", which are:

Firt_SampleData_R1.fastq.gz
Firt_SampleData_R2.fastq.gz
Second_SampleData_R1.fastq.gz
Second_SampleData_R2.fastq.gz

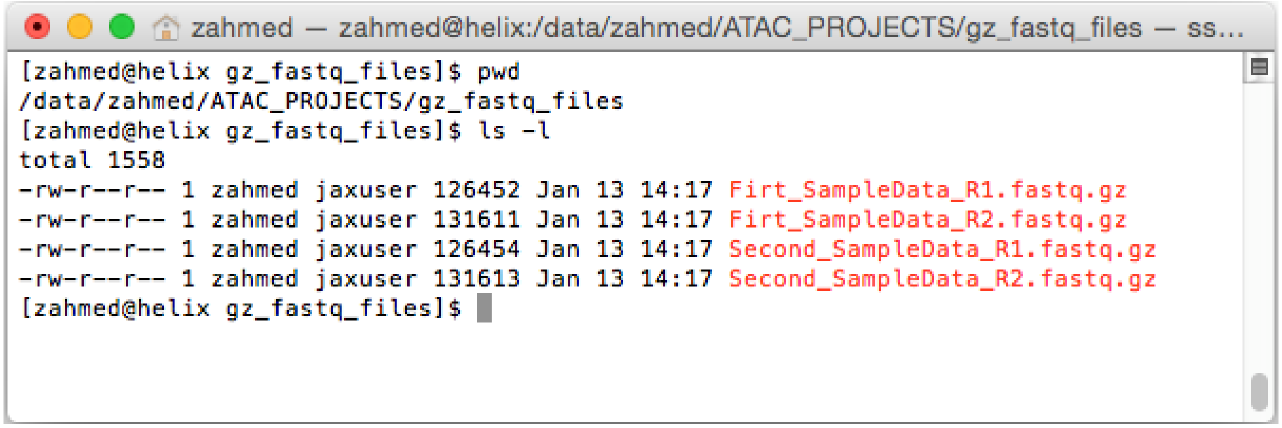

S-Fig. 10: Screen shot (Linux Terminal, using Mac-OS-X) of compressed sample data files

After setting parameters and input path to the I-ATAQ-seq, pressed button "Run ATAC-seq", an information message will appear (S-Fig. 11) to verify the input sample data source location, output directory location and set job parameters.

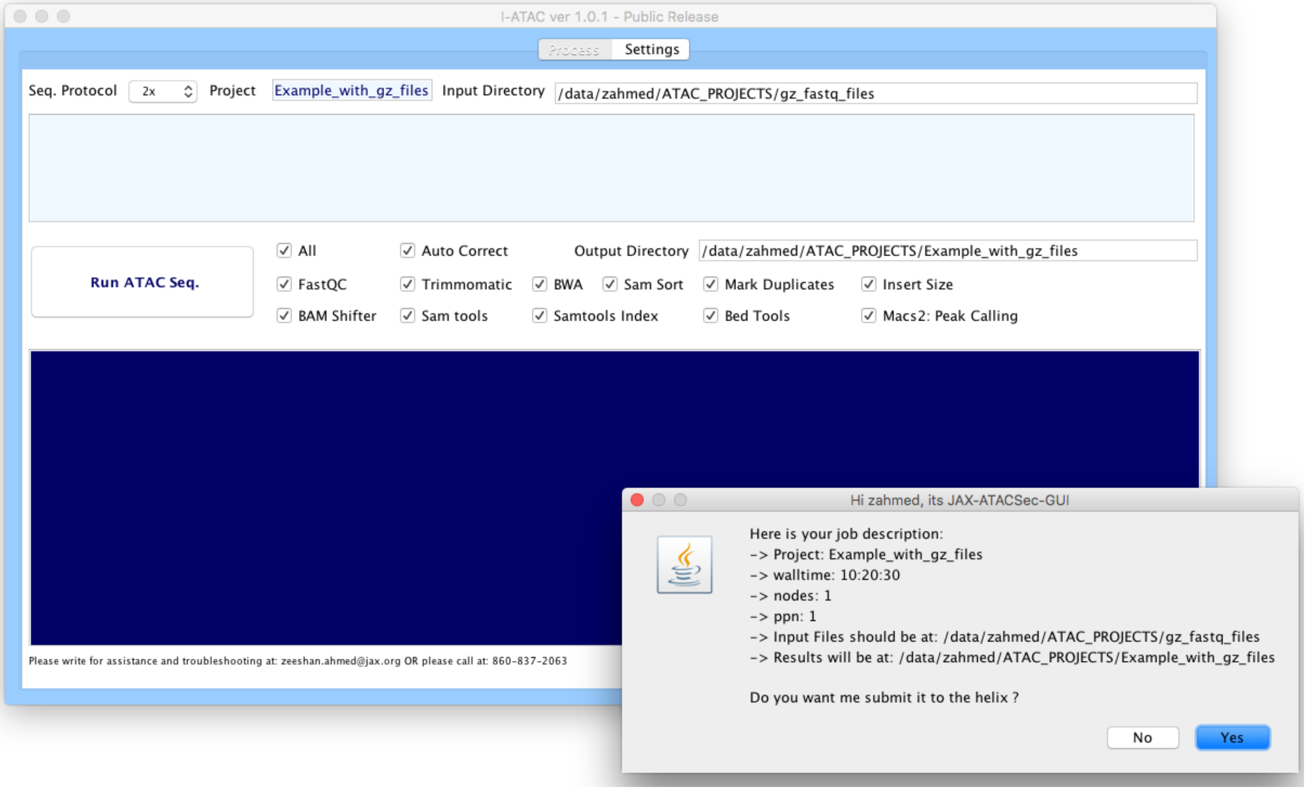

S-Fig. 11: I-ATAC, input sample data and set parameters' verification

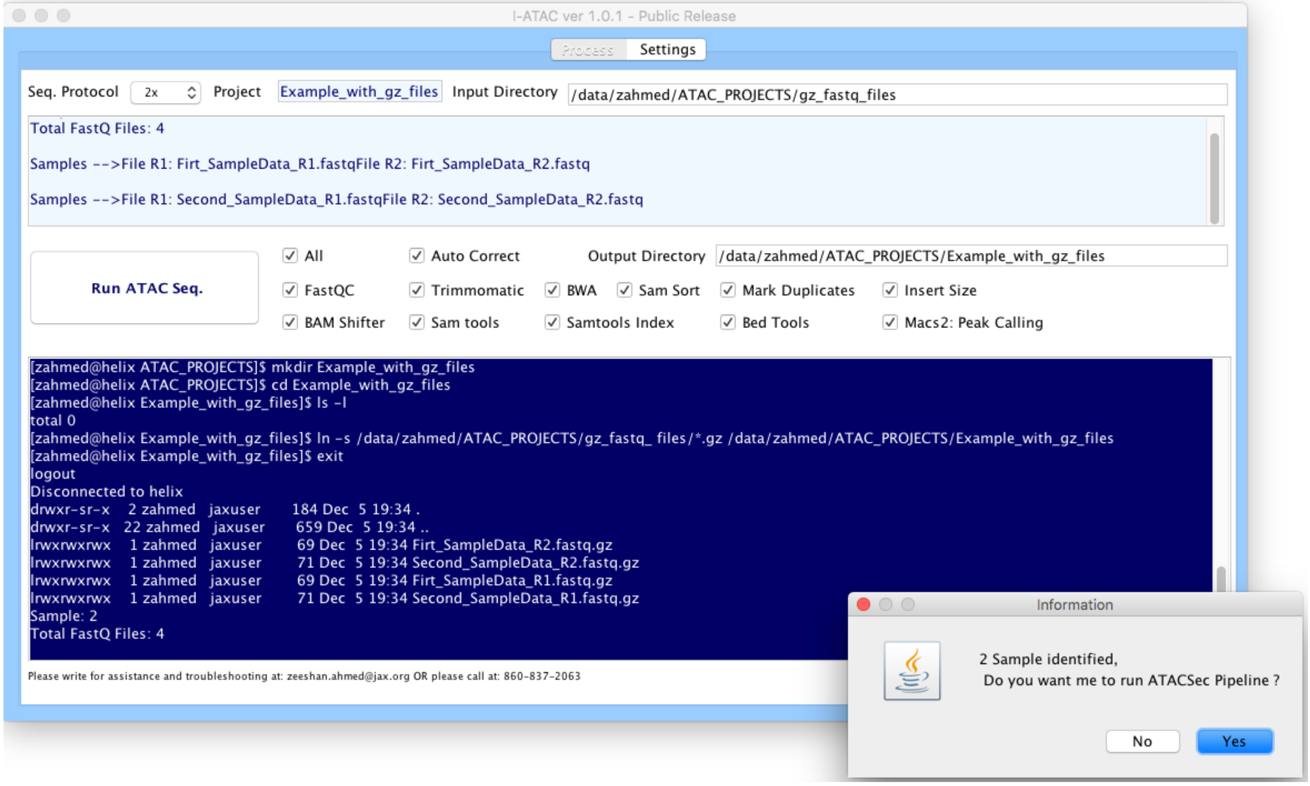

S-Fig. 12: I-ATAC, identified sample data

At successful verification, file status window (S-Fig. 12) provides the information about located sample data files, which were copied, pasted and unzipped in the project directory (Example_with_gz_files). At second successful verification, the ATAC-seq data processing pipeline was automatically scripted (S-Fig. 13) and created job was queued to the data cluster (S-Fig. 14).

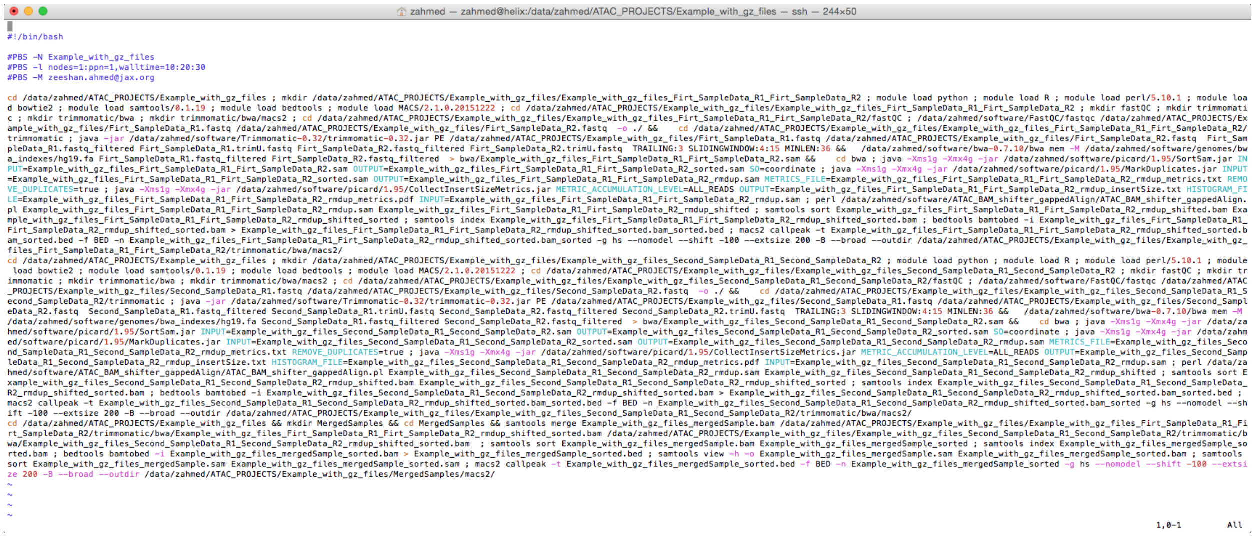

S-S-Fig. 13: I-ATAC automatically generated script

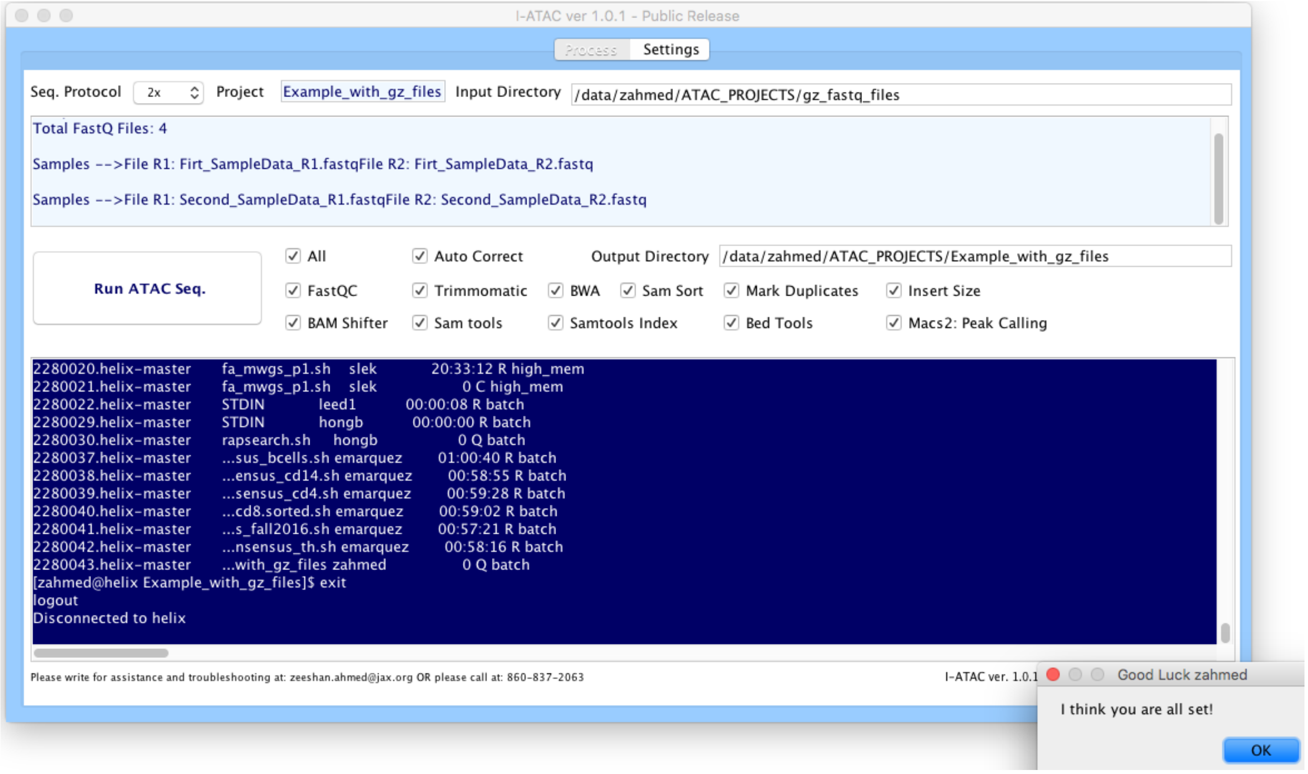

S-Fig. 14: I-ATAC job queued.

7.1.3 Output

After the successful execution of the ATAC-seq data processing pipeline, the system's generated output can be located in the mentioned output directory (S-Fig. 15). The project directory contains automatically generated and run script:

Example_with_gz_files_Example_with_gz_files.sh

copied, pasted and unzipped "FASTQ" files:

Firt_SampleData_R1.fastq
Firt_SampleData_R2.fastq
Second_SampleData_R1.fastq
Second_SampleData_R2.fastq

and sub-directories:

Example_with_gz_files_Firt_SampleData_R1_Firt_SampleData_R2,
Example_with_gz_files_Second_SampleData_R1_Second_SampleData_R2,
MergedSamples

The output files were placed in proposed system's automatically created sub-directory structure (Section: Applications integration, data processing pipeline and project's directory structure), as shown in S-Fig. 14. We also input two samples and asked system to produce merged replicates as well. So, we observed results for both samples as well as merged replicates.

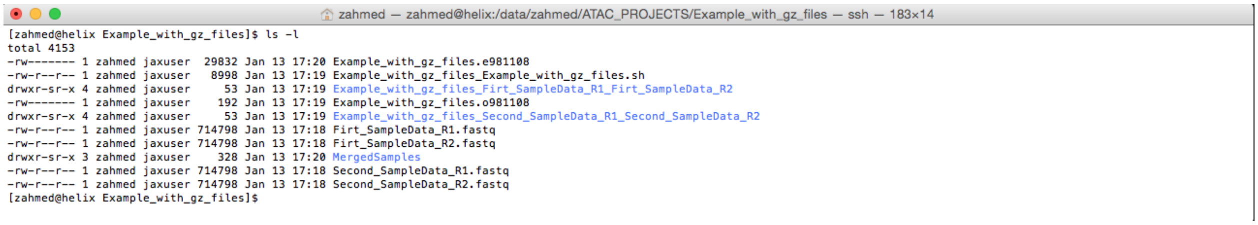

S-Fig. 15: Screen shot (Linux Terminal, using Mac-OS-X) of produced I-ATAC output project directory and files

The produced results from First_SampleData are shown in S-Fig. 16, including quality reports in FASTQC directory, which are:

Firt_SampleData_R1_fastqc.html
Firt_SampleData_R1_fastqc.zip
Firt_SampleData_R2_fastqc.html
Firt_SampleData_R2_fastqc.zip

trimmed “FASTQ” files in trimmomatic directory, which are:

Firt_SampleData_R1.fastq_filtered
Firt_SampleData_R1.trimU.fastq
Firt_SampleData_R2.fastq_filtered
Firt_SampleData_R2.trimU.fastq

all sorted, shifted and indexed “sam” and “bam”, “bed” and related files are placed in “bwa” directory, which are:

Example_with_gz_files_Firt_SampleData_R1_Firt_SampleData_R2.sam
Example_with_gz_files_Firt_SampleData_R1_Firt_SampleData_R2_sorted.sam
Example_with_gz_files_Firt_SampleData_R1_Firt_SampleData_R2_rmdup.sam
Example_with_gz_files_Firt_SampleData_R1_Firt_SampleData_R2_rmdup_shifted.bam
Example_with_gz_files_Firt_SampleData_R1_Firt_SampleData_R2_rmdup_shifted_sorted.bam
Example_with_gz_files_Firt_SampleData_R1_Firt_SampleData_R2_rmdup_shifted_sorted.bam.bai
Example_with_gz_files_Firt_SampleData_R1_Firt_SampleData_R2_rmdup_shifted_sorted.bam_sorted.bed
Example_with_gz_files_Firt_SampleData_R1_Firt_SampleData_R2_rmdup_metrics.txt
Example_with_gz_files_Firt_SampleData_R1_Firt_SampleData_R2_rmdup_metrics.pdf
Example_with_gz_files_Firt_SampleData_R1_Firt_SampleData_R2_rmdup_insertSize.txt

and all produced results at peak calling were placed in “macs2” directory, which are:

Example_with_gz_files_Firt_SampleData_R1_Firt_SampleData_R2_rmdup_shifted_sorted.bam_sorted_control_lambda.bdg
Example_with_gz_files_Firt_SampleData_R1_Firt_SampleData_R2_rmdup_shifted_sorted.bam_sorted_peaks.broadPeak
Example_with_gz_files_Firt_SampleData_R1_Firt_SampleData_R2_rmdup_shifted_sorted.bam_sorted_peaks.gappedPeak
Example_with_gz_files_Firt_SampleData_R1_Firt_SampleData_R2_rmdup_shifted_sorted.bam_sorted_peaks.xls
Example_with_gz_files_Firt_SampleData_R1_Firt_SampleData_R2_rmdup_shifted_sorted.bam_sorted_treat_pileup.bdg

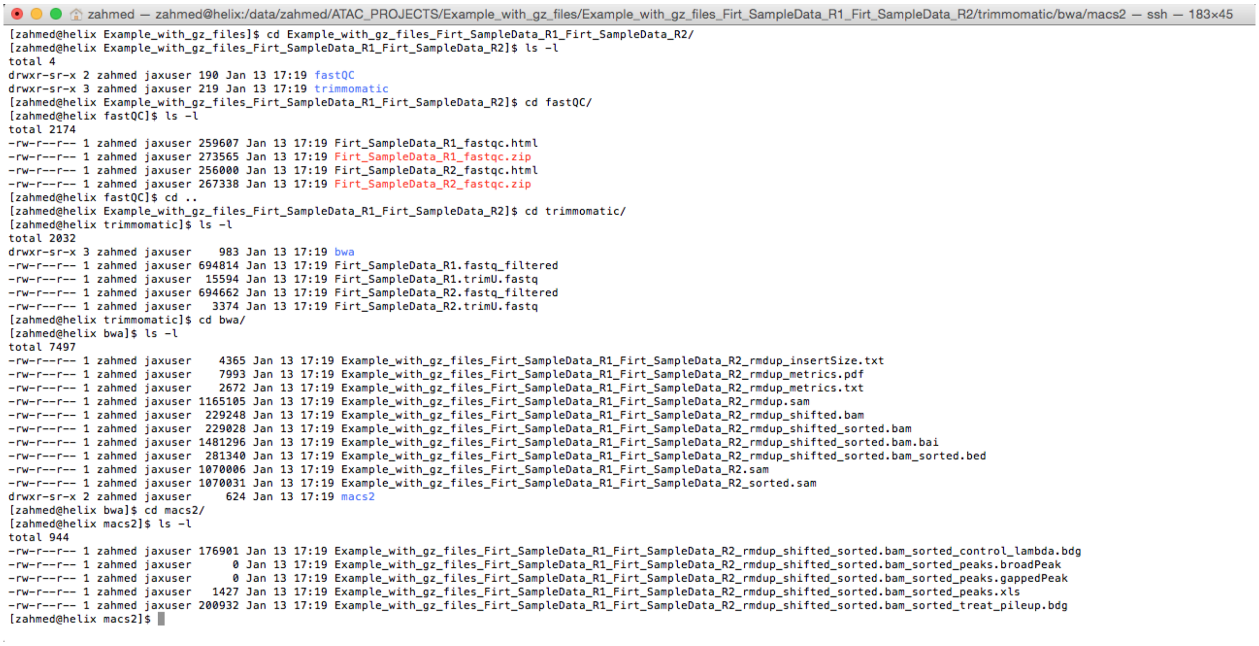

S-Fig. 16: Screen shot (Linux Terminal, using Mac-OS-X) of produced I-ATAC output files for Firt_SampleData

Likewise, First_SampleData, the produced results from Second_SampleData are shown in S-Fig. 17.

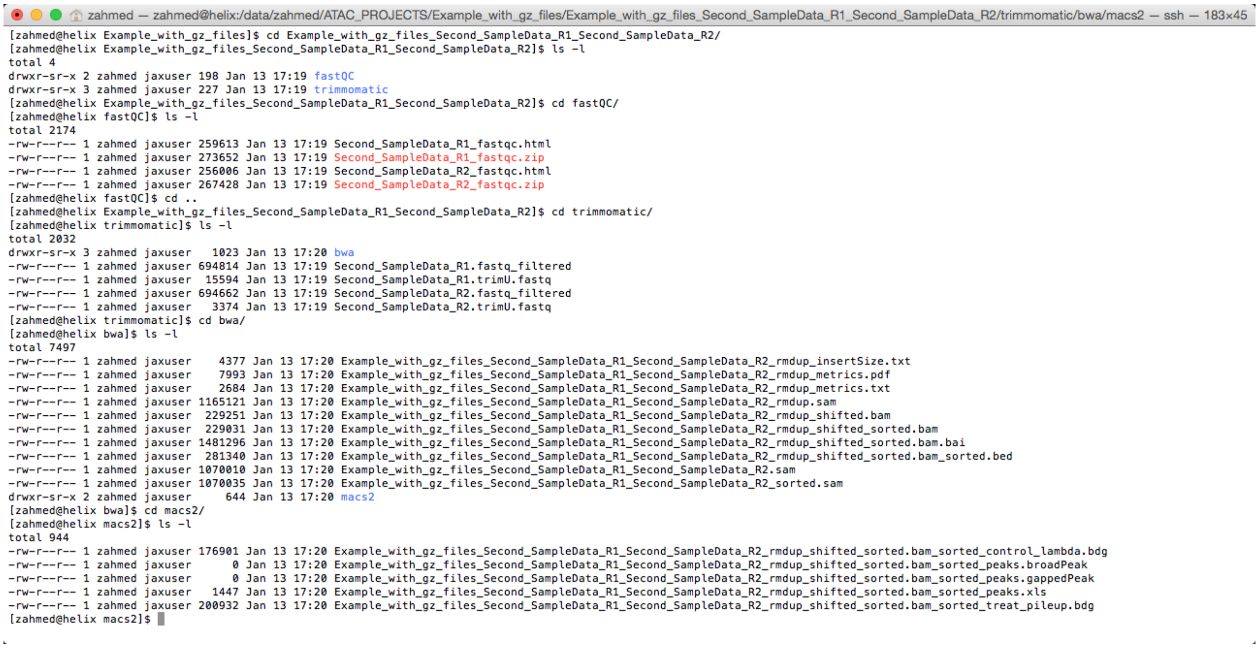

S-Fig. 17: Screen shot (Linux Terminal, using Mac-OS-X) of produced I-ATAC output files for Second_SampleData

The produced results from merged samples are shown in S-Fig. 18.

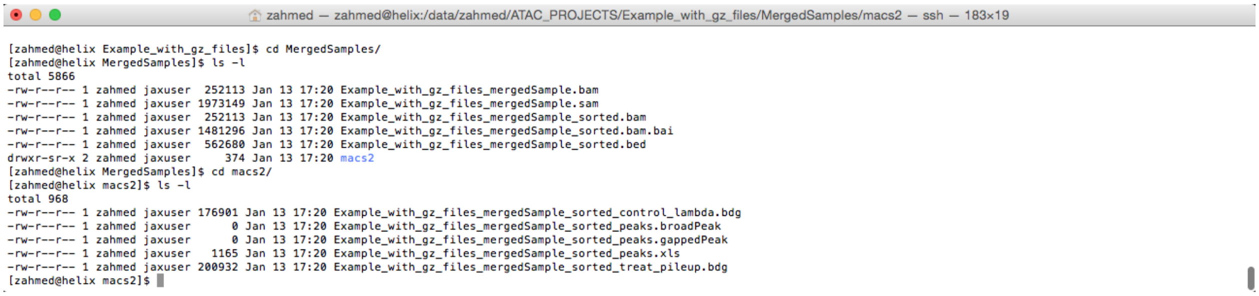

S-Fig. 18: Screen shot (Linux Terminal, using Mac-OS-X) of produced I-ATAC output files for Merged Samples

7.2 Case Study 2: Using GM12878 – CD4 T- Cells

7.2.1 Dataset Details

Information about Raw dataset (GM12878 – CD4 T- Cell, Day 1, Rep1 SRR891275 and Rep2 SRR891276) is available at web link (https://catalog.coriell.org/0/Sections/Search/Sample_Detail.aspx?Ref=GM12878&product=CC and produced results, which are mentioned in this case study can be downloaded from the following project web link.

7.2.2 Input

The input to I-ATAC is the path to ATAC-seq sample data (S-Fig. 19), which in our case is:

"/data/zahmed/ATAC_seq_data/CD4"

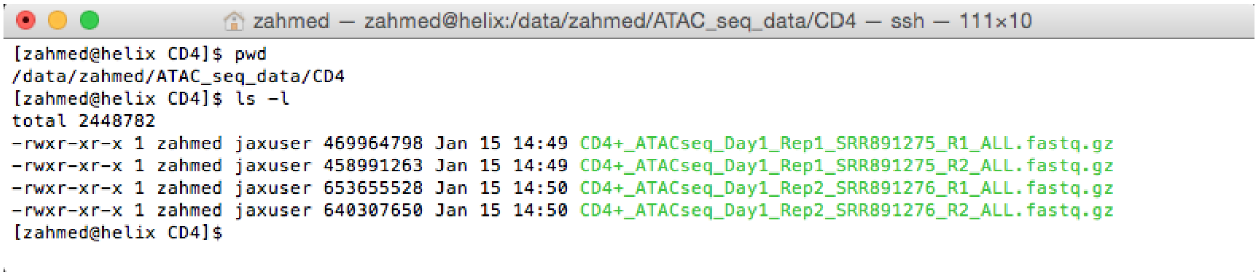

S-Fig. 19: Screen shot (Linux Terminal, using Mac-OS-X) of compressed sample data files of GM12878 – CD4 T- Cell, Day 1, Rep1 SRR891275 and Rep2 SRR891276.

Likewise, earlier discussed case study, at successful identification and verification of sample data files (S-Fig. 20), data processing job was created (S-Fig. 21) and successfully queued (S-Fig. 22).

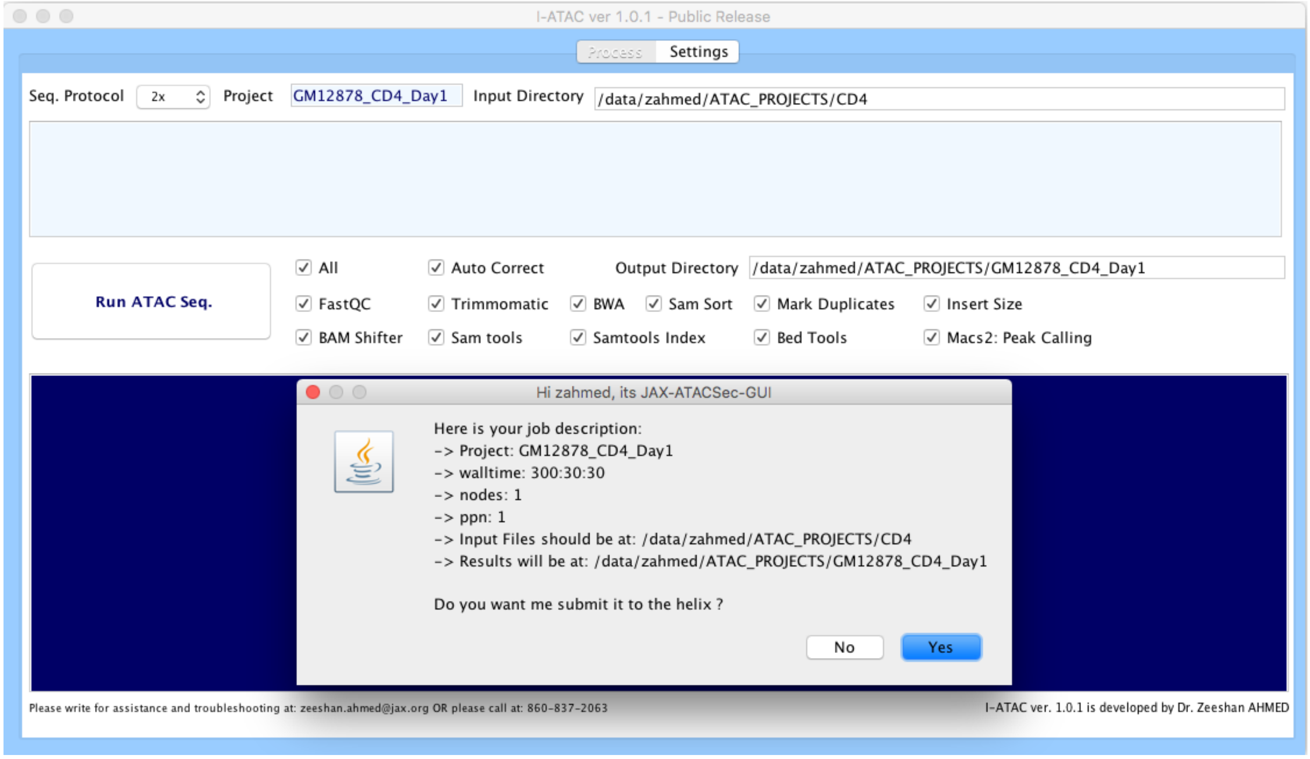

S-Fig. 20: I-ATAC, input sample data, set parameters and verification

/data/zahmed/ATAC_PROJECTS/CD4
GM12878_CD4_Day1

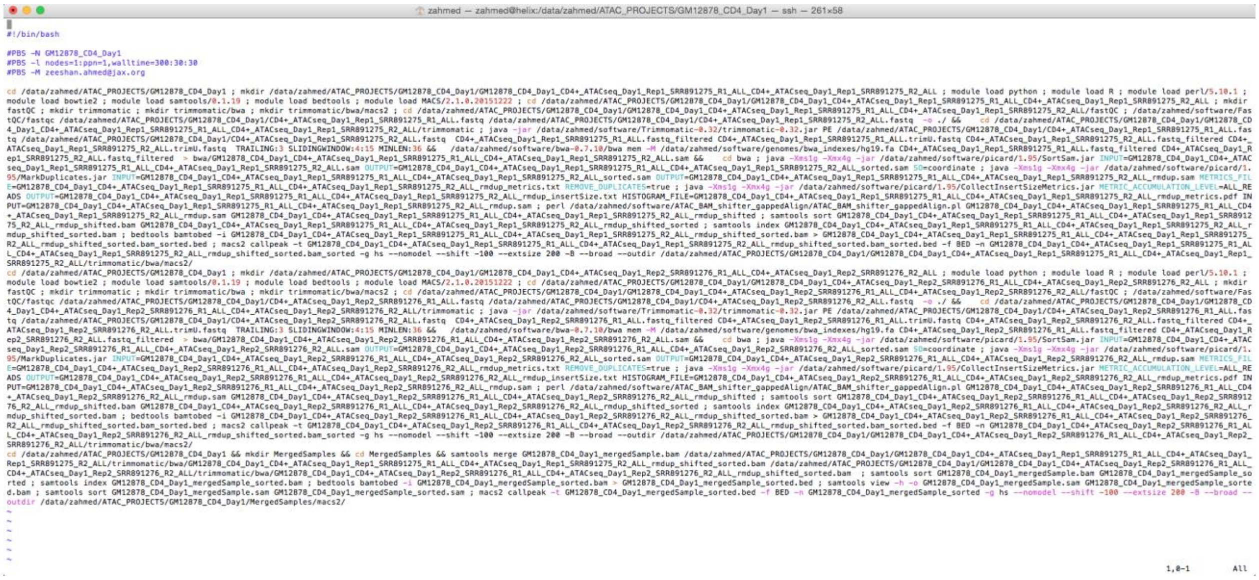

S-Fig. 21: I-ATAC generated script

7.2.3 Output

As in the earlier discussed case study, all the produced results were placed in the proposed and auto generated directory structure (S-Fig. 22, 23and 24).

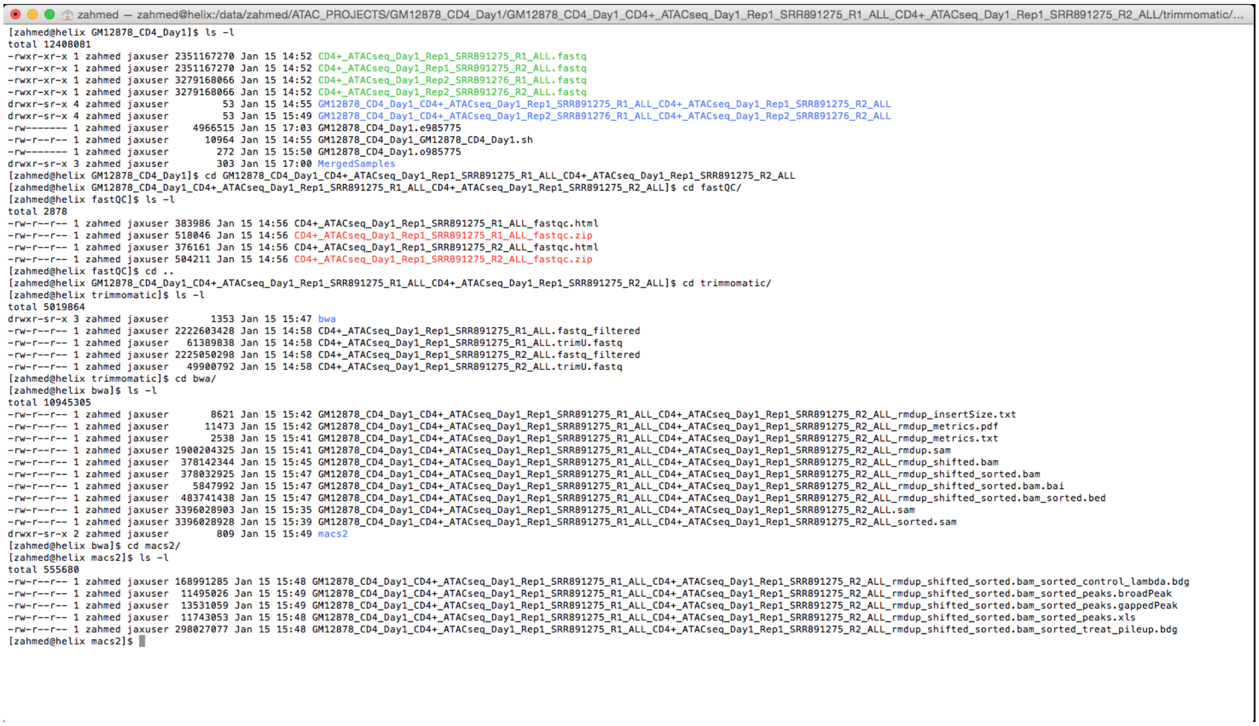

S-Fig. 22: Screen shot (Linux Terminal, using Mac-OS-X) of produced I-ATAC output main project directory and files, and for sample CD4+_ATACseq_Day1_Rep1_SRR891275

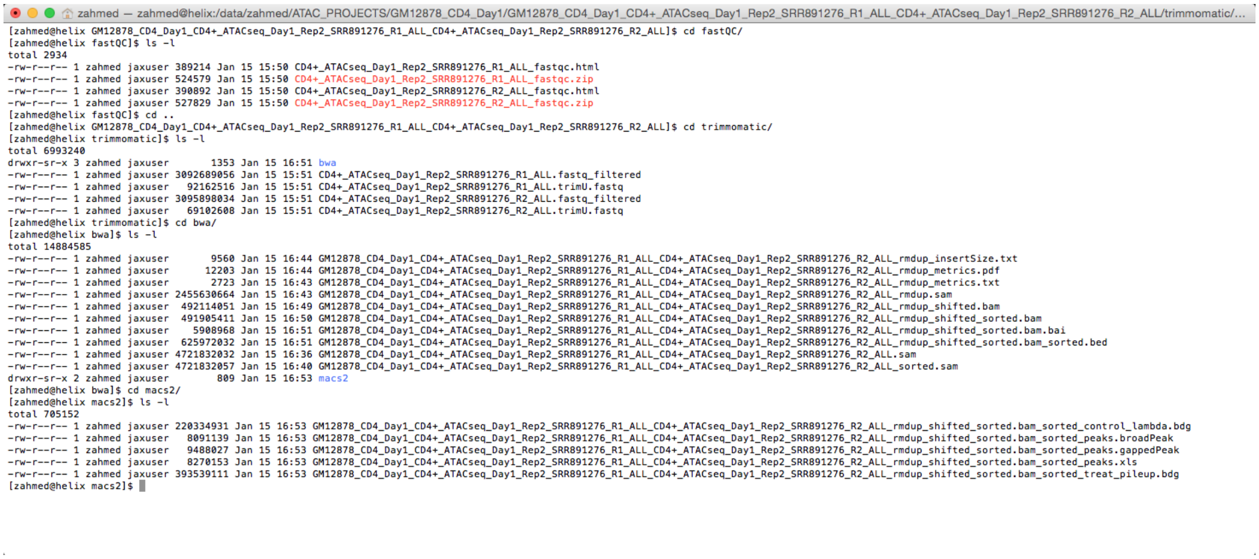

S-Fig. 23: Screen shot (Linux Terminal, using Mac-OS-X) of produced I-ATAC output files for sample CD4+_ATACseq_Day1_Rep2_SRR891276

```
zahmed — zahmed@helix:/data/zahmed/ATAC_PROJECTS/GM12878_CD4_Day1/MergedSamples/macs2 — ssh — 142×20
[zahmed@helix GM12878_CD4_Day1]$ cd MergedSamples/
[zahmed@helix MergedSamples]$ ls -l
total 7175522
-rw-r--r-- 1 zahmed jaxuser  861237223 Jan 15 16:55 GM12878_CD4_Day1_mergedSample.bam
-rw-r--r-- 1 zahmed jaxuser 3676571000 Jan 15 17:00 GM12878_CD4_Day1_mergedSample.sam
-rw-r--r-- 1 zahmed jaxuser  861237223 Jan 15 16:59 GM12878_CD4_Day1_mergedSample_sorted.bam
-rw-r--r-- 1 zahmed jaxuser    6057328 Jan 15 16:59 GM12878_CD4_Day1_mergedSample_sorted.bam.bai
-rw-r--r-- 1 zahmed jaxuser 1109713470 Jan 15 17:00 GM12878_CD4_Day1_mergedSample_sorted.bed
drwxr-sr-x 2 zahmed jaxuser        349 Jan 15 17:03 macs2
[zahmed@helix MergedSamples]$ cd macs2/
[zahmed@helix macs2]$ ls -l
total 693456
-rw-r--r-- 1 zahmed jaxuser 206569472 Jan 15 17:02 GM12878_CD4_Day1_mergedSample_sorted_control_lambda.bdg
-rw-r--r-- 1 zahmed jaxuser   5015956 Jan 15 17:03 GM12878_CD4_Day1_mergedSample_sorted_peaks.broadPeak
-rw-r--r-- 1 zahmed jaxuser   6688771 Jan 15 17:03 GM12878_CD4_Day1_mergedSample_sorted_peaks.gappedPeak
-rw-r--r-- 1 zahmed jaxuser   5231644 Jan 15 17:03 GM12878_CD4_Day1_mergedSample_sorted_peaks.xls
-rw-r--r-- 1 zahmed jaxuser 405274624 Jan 15 17:02 GM12878_CD4_Day1_mergedSample_sorted_treat_pileup.bdg
[zahmed@helix macs2]$
```

S-Fig. 24: Screen shot (Linux Terminal, using Mac-OS-X) of produced I-ATAC output files for Merged Samples

Basic Statistics

Measure	Value
Filename	CD4+_ATACseq_Day1_Rep1_SRR891275_R1_ALL.fastq
File type	Conventional base calls
Encoding	Sanger / Illumina 1.9
Total Sequences	8086940
Sequences flagged as poor quality	0
Sequence length	50
%GC	44

(A)

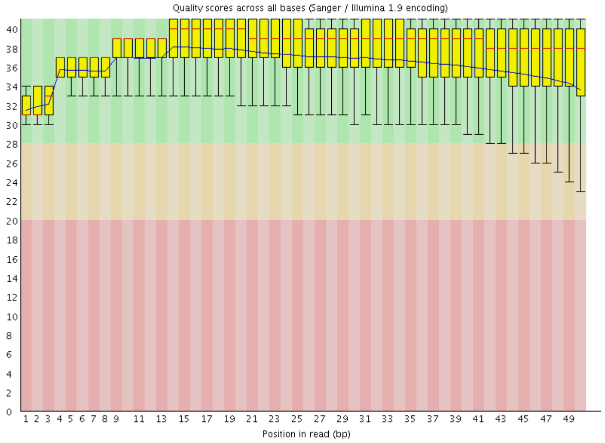

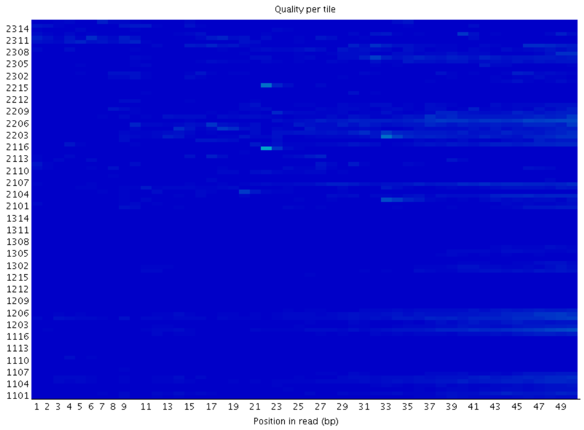

(B) (C)

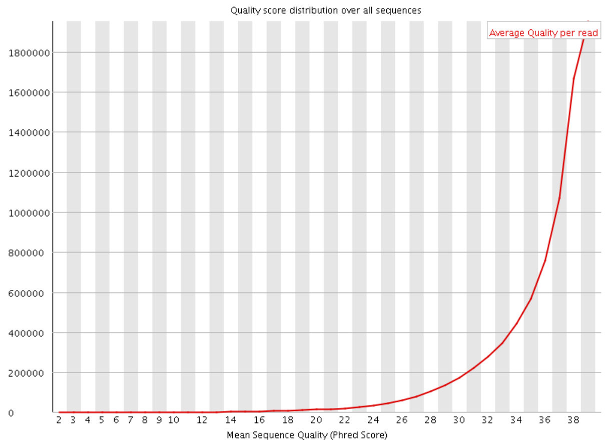
Quality score distribution over all sequences
Average Quality per read
Mean Sequence Quality (Phred Score)

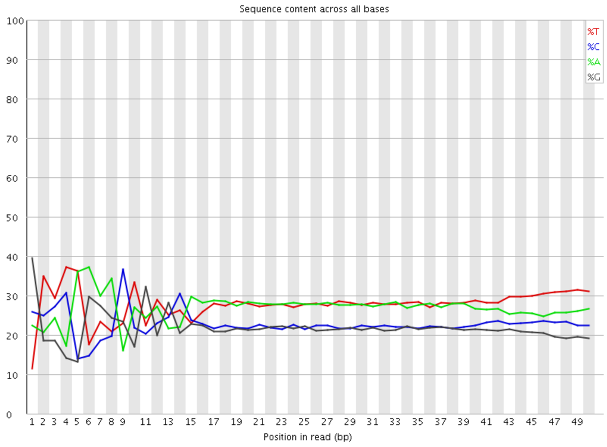
Sequence content across all bases
%T
%C
%A
%G
Position in read (bp)

(D) (E)

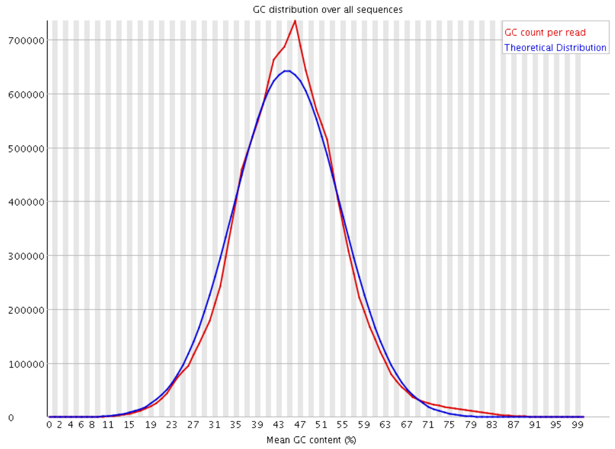
GC distribution over all sequences
GC count per read
Theoretical Distribution
Mean GC content (%)

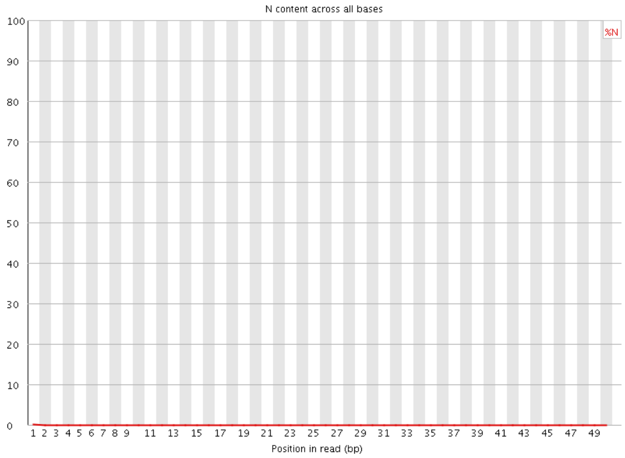
N content across all bases
%N
Position in read (bp)

(F) (G)

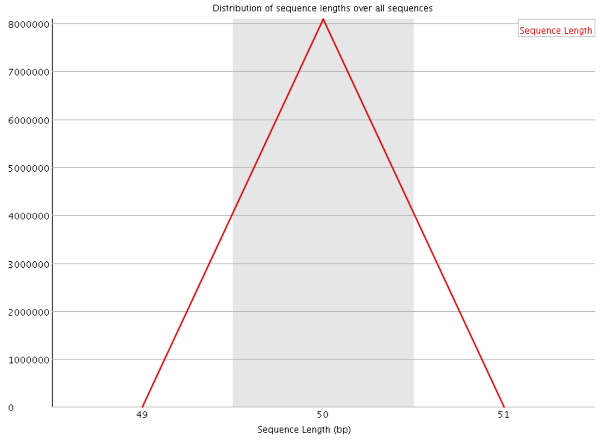
Distribution of sequence lengths over all sequences
Sequence Length
Sequence Length (bp)

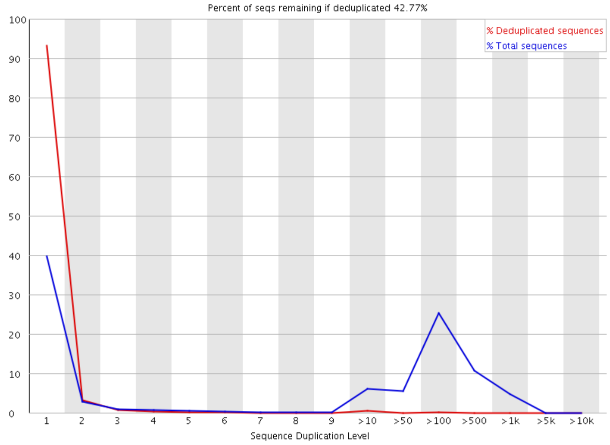
Percent of seqs remaining if deduplicated 42.77%
% Deduplicated sequences
% Total sequences
Sequence Duplication Level

(H) (I)

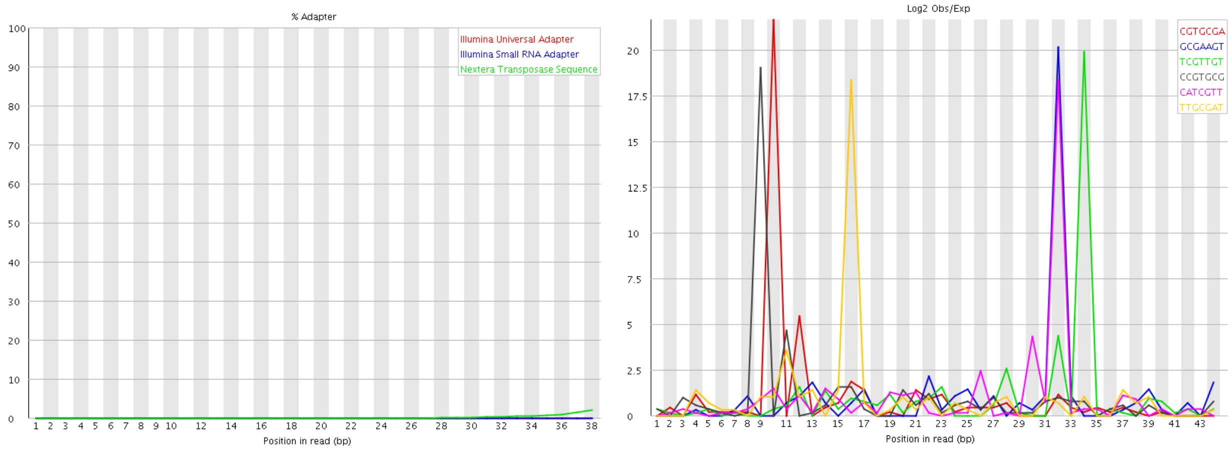

(J) (K)

S-Fig. 25. Statistics and quality results produced by the FASTQC for the CD4+_ATACseq_Day1_Rep1_SRR891275.

Quality stats produced by the FASTQC (at CD4+_ATACseq_Day1_Rep1_SRR891275) are shown in S-Fig. 26, which are: Basic Statistics (S-Fig. 25. A), Per base sequence quality (S-Fig. 25. B), Per tile sequence quality (S-Fig. 25. C), Per sequence quality scores (S-Fig. 25. D), Per base sequence content (S-Fig. 25. E), Per sequence GC content (S-Fig. 25. F), Per base N content (S-Fig. 25. G), Sequence Length Distribution (S-Fig. 25. H), Sequence Duplication Levels (S-Fig. 25. I), Adapter Content (S-Fig. 25. J) and Kmer Content (S-Fig. 25. K).

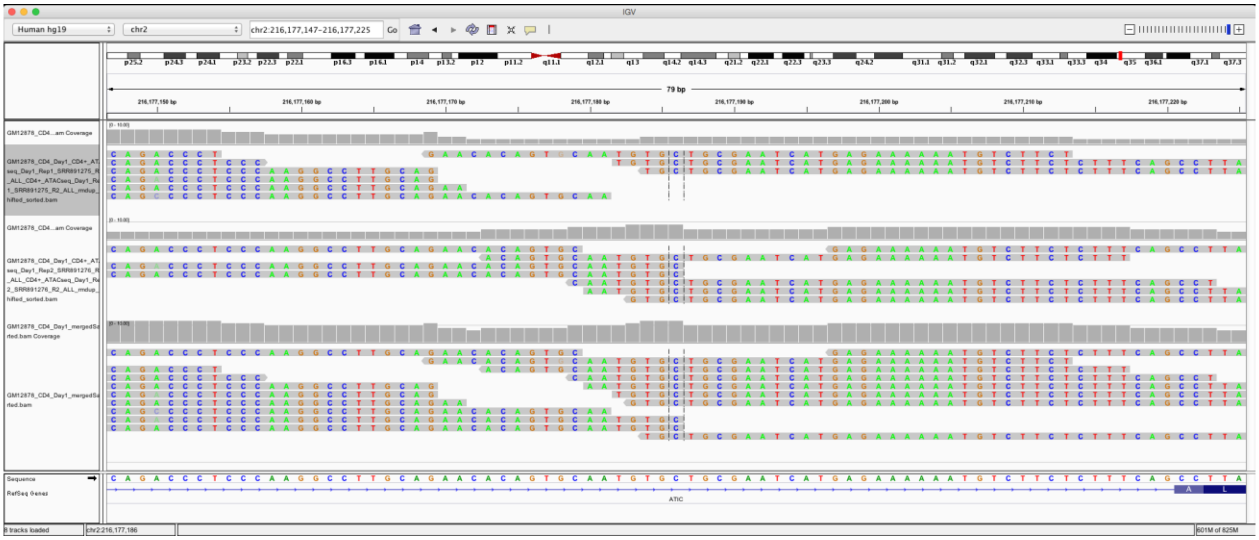

S-Fig. 26. Example Visualization of “bam” files (CD4+_ATACseq_Day1_Rep1_SRR891275, CD4+_ATACseq_Day1_Rep2_SRR891276 and GM12878_CD4_Day1_mergedSample) using IGV.

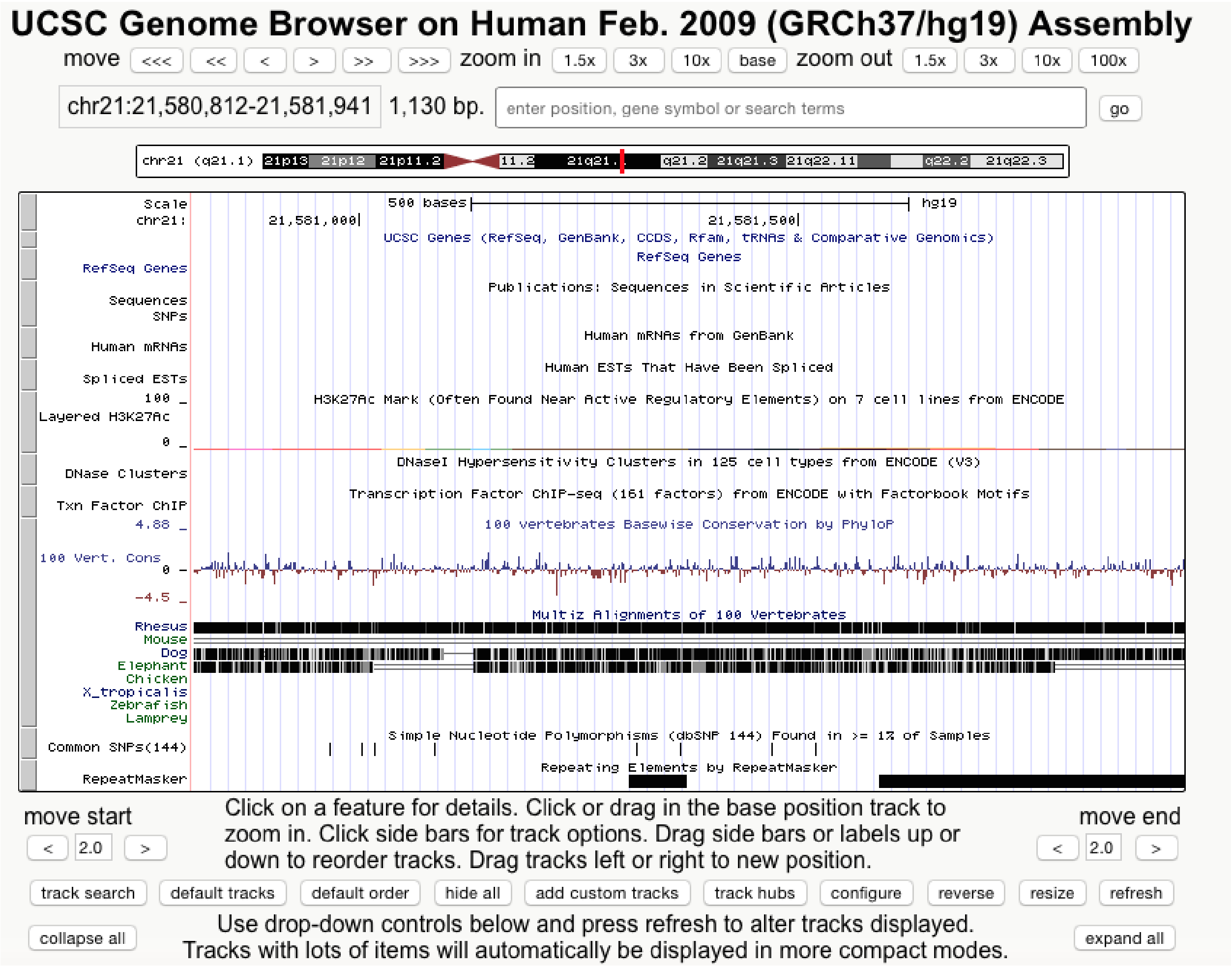

S-Fig. 27 Example Visualization using "bdg" file (GM12878_CD4_Day1_mergedSample_sorted_control_lambda.bdg) using USCS Genome Browser.

The detailed output of samples a CD4+_ATACseq_Day1_Rep1_SRR891275 and CD4+_ATACseq_Day1_Rep2_SRR891276 are attached in the supplementary material. Example visualization of produced results is created by visualizing sorted „bam" files (CD4+_ATACseq_Day1_Rep1_SRR891275, CD4+_ATACseq_Day1_Rep2_SRR891276 and GM12878_CD4_Day1_mergedSample) using IGV (S-Fig. 26) and peak file (CD4+_ATACseq_Day1_Rep1_SRR891275) using USCS Genome browser (S-Fig. 27).

8 Conclusions

To the best of our knowledge, I-ATAC platform is the first desktop tool that is specialized to processing and analysis of ATAC-seq data. I-ATAC provides a flexible algorithm and parameter setting GUI for non-computational scientists and a time-efficient parallel data analysis environment for computational scientists. Future work includes incorporating visualization and differential analysis modules in I-ATAC platform.

9 Acknowledgments

The Jackson Laboratory (JAX) supports and owns this project. Special thanks to Ucar and Banchereau labs as well as the Genome Technologies and Computational Sciences cores at JAX, who provided insight and expertise that greatly, assisted the research and development of this platform.

10 Funding:

This work was supported by The Jackson Laboratory, USA.

11 Conflict of Interests:

The authors declare that they have no competing interests.

12 Additional Requirements

For additional information, please refer to the project webpage: https://www.jax.org/research-and-faculty/tools/i-atac

Source code, JAR files for MAC OS X and Windows, and complete source code package for Eclipse IDE is available at https://github.com/UcarLab/I-ATAC

Example dataset is available at: https://zenodo.org/record/46079#.WAe3l5MrK7Y

Supporting software and dependencies are available at: https://zenodo.org/record/162023#.WAe3dJMrK7Y

13 References

Ahmed Z, Ucar D. (2017) A standalone software platform for the interactive management and pre-processing of ATAC-seq samples. *PeerJ Preprints.*, **5**:e2942v1 https://doi.org/10.7287/peerj.preprints.2942v1

Ahmed Z, Zeeshan S, Dandekar T. (2014) Developing sustainable software solutions for bioinformatics by the "Butterfly" paradigm. *F1000Res*., **3**, 71.

Bauch A. (2011) openBIS: a flexible framework for managing and analyzing complex data in biology research. *BMC Bioinf*., **12**, 468.

Buenrostro JD, Giresi PG, Zaba LC, Chang HY, Greenleaf WJ. *et al*. (2013) Transposition of native chromatin for fast and sensitive epigenomic profiling of open chromatin, DNA-binding proteins and nucleosome position. *Nat Methods*., **10**, 1213-1218.

Bolger AM, Lohse M, Usadel B. (2014) Trimmomatic: a flexible trimmer for Illumina sequence data. *Bioinformatics*., **30**, 2114-20.

Dander A, Pabinger S, Sperk M, Fischer M, Stocker G, Trajanoski Z. (2014) SeqBench: integrated solution for the management and analysis of exome sequencing data. *BMC Res Notes*., **7**, 43.

Giardine B. (2005) Galaxy: a platform for interactive large-scale genome analysis. *Genome Res*. **15**, 1451-1455.

Horak CE, Snyder M. (2002) ChIP-chip: A genomic approach for identifying transcription factor binding sites. *Methods Enzymol*., **350**, 469–483

Li H, Durbin R. (2009) Fast and accurate short read alignment with Burrows-Wheeler Transform. *Bioinformatics*., **25**, 1754-60.

Li H, Handsaker B, Wysoker A, Fennell T, Ruan J, Homer N, Marth G, Abecasis G, Durbin R; 1000 Genome Project Data Processing Subgroup. (2009) The Sequence alignment/map (SAM) format and SAMtools. *Bioinformatics.*, **25**, 2078-2079.

Mariette J, Escudié F, Allias N, Salin G, Noirot C, Thomas S, Klopp C. (2012) NG6: Integrated next generation sequencing storage and processing environment. *BMC Genomics*. **13**, 462.

McLellan A S, Dubin RA, Jing Q, Broin PÓ, Moskowitz D, Suzuki M, Calder RB, Hargitai J, Golden A, Greally JM. (2012) The Wasp System: an open source environment for managing and analyzing genomic data. *Genomics*., **100**, 345-51.

Mount DM. (2004). Bioinformatics: Sequence and Genome Analysis (2nd ed.). Cold Spring Harbor Laboratory Press: Cold Spring Harbor, NY. ISBN 0-87969-608-7.

Orvis J, Crabtree J, Galens K, Gussman A, Inman JM, Lee E, Nampally S, Riley D, Sundaram JP, Felix V, Whitty B, Mahurkar A, Wortman J, White O, Angiuoli SV. (2010) Ergatis: a web interface and scalable software system for bioinformatics workflows. *Bioinformatics*, **26**, 1488-92.

Quinlan,A R, Hall IM. (2010) BEDTools: a flexible suite of utilities for comparing genomic features. *Bioinformatics*., **26**, 841-842.

Scholtalbers J. (2013) Galaxy LIMS for next-generation sequencing. *Bioinformatics*. **29**,1233-1234.

Tsompana M, Buck MJ. (2014) Chromatin accessibility: a window into the genome. *Epigenetics & Chromatin*., **7**, 33.

Venco F, Vaskin Y, Ceol A, Muller H. (2014) SMITH: a LIMS for handling next-generation sequencing workflows. *BMC Bioinf*., **15**, (Suppl 14):S3.

Zhang Y, Liu T, Meyer CA, Eeckhoute J, Johnson DS, Bernstein BE, Nusbaum C, Myers RM, Brown M, Li W, Liu XS. (2008) Model-based analysis of ChIP-Seq (MACS). *Genome Biol.*, **9**, R137.
