# Supplementary material for: I-ATAC: interactive pipeline for the management and pre-processing of ATAC-seq samples
Source: PeerJ. 2017 Nov 22;5:e4040. doi: 10.7717/peerj.4040 (PMC5702251; doi:10.7717/peerj.4040)
Supplement: Supplemental Information 2 [file peerj-05-4040-s002.docx]

Supplementary Material: Figures

I-ATAC: Interactive pipeline for the management and pre-processing of ATAC-seq samples

Zeeshan Ahmed^1^ and Duygu Ucar^2^

^1^University of Connecticut Health Center, 195 Farmington Ave, Farmington, CT, USA

^2^The Jackson Laboratory for Genomic Medicine, 10 Discovery Dr., Farmington, CT, USA

Corresponding authors: Zeeshan Ahmed^1^ and Duygu Ucar^2^

Email address: [zahmed@uchc.edu](mailto:zahmed@uchc.edu) and [duygu.ucar@jax.org](mailto:duygu.ucar@jax.org)


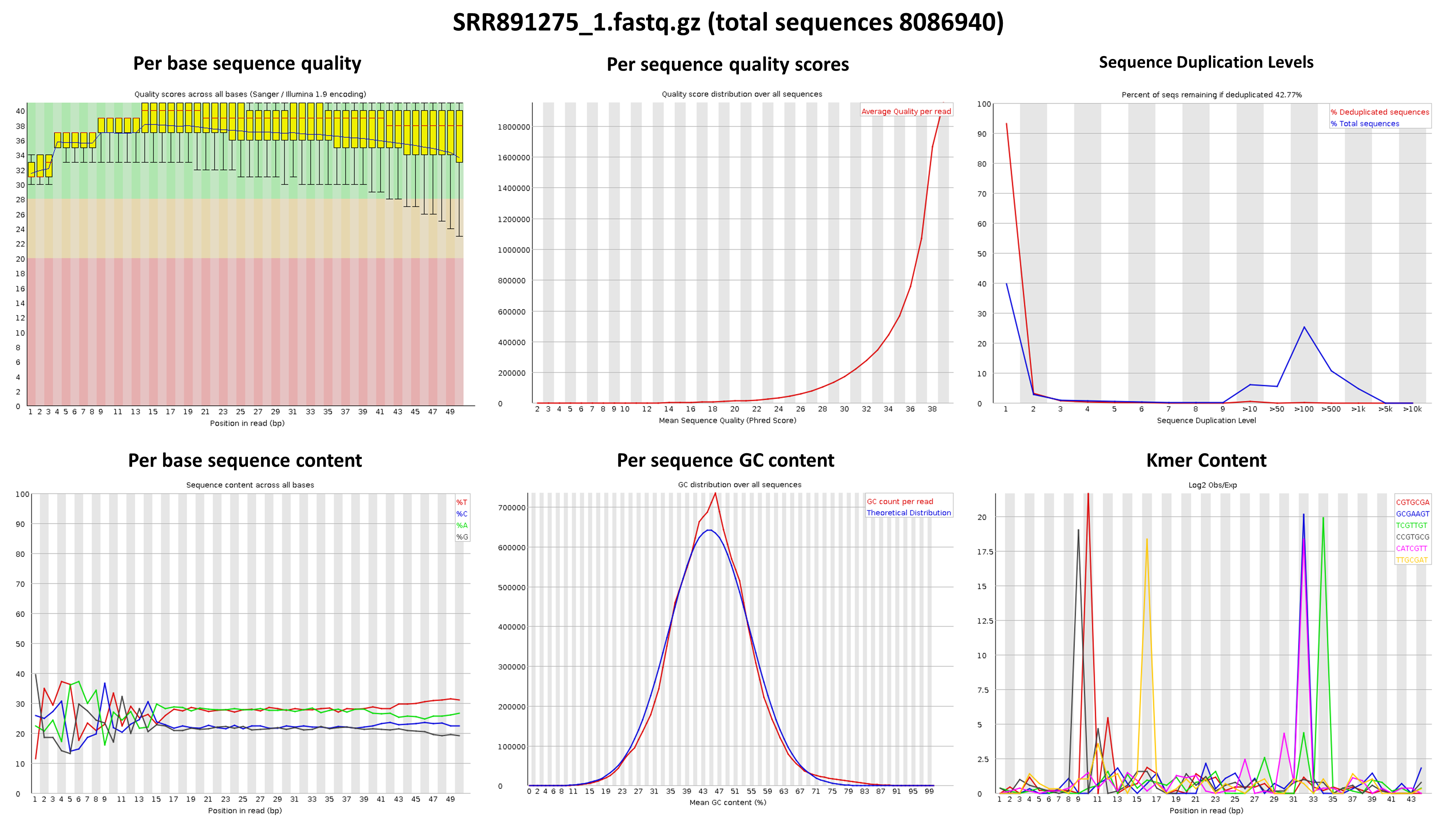


A.


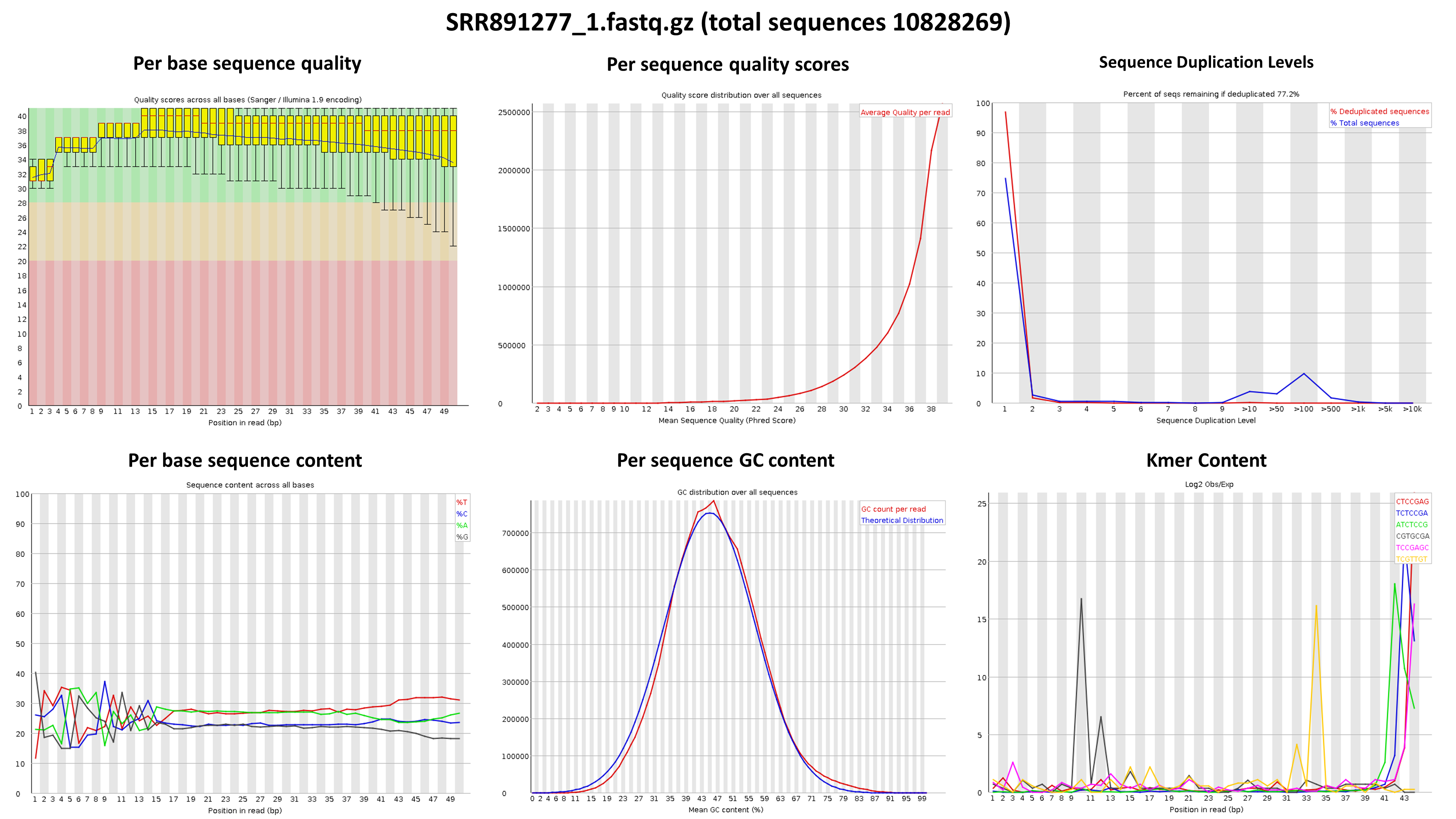


B.


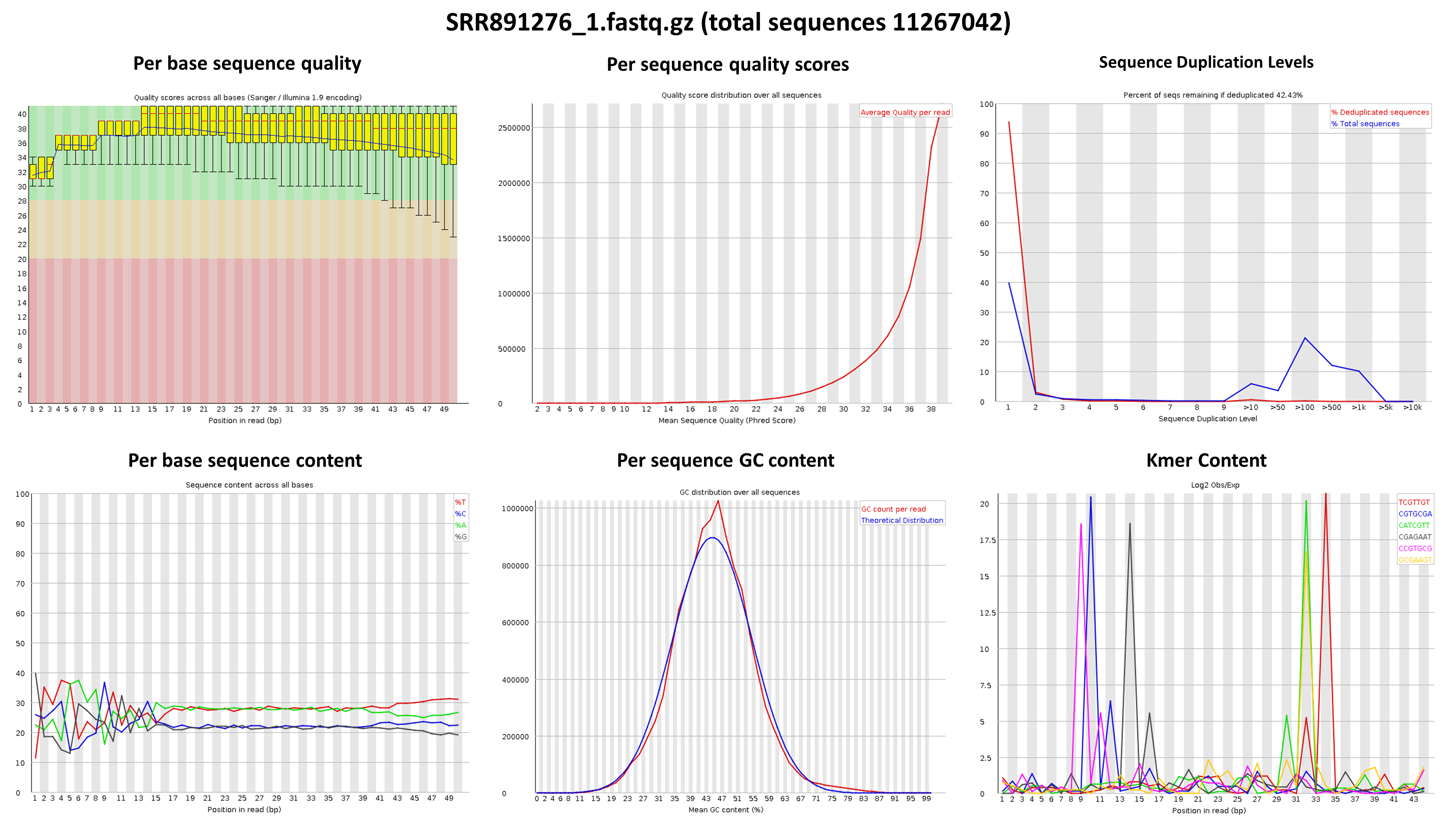


C.


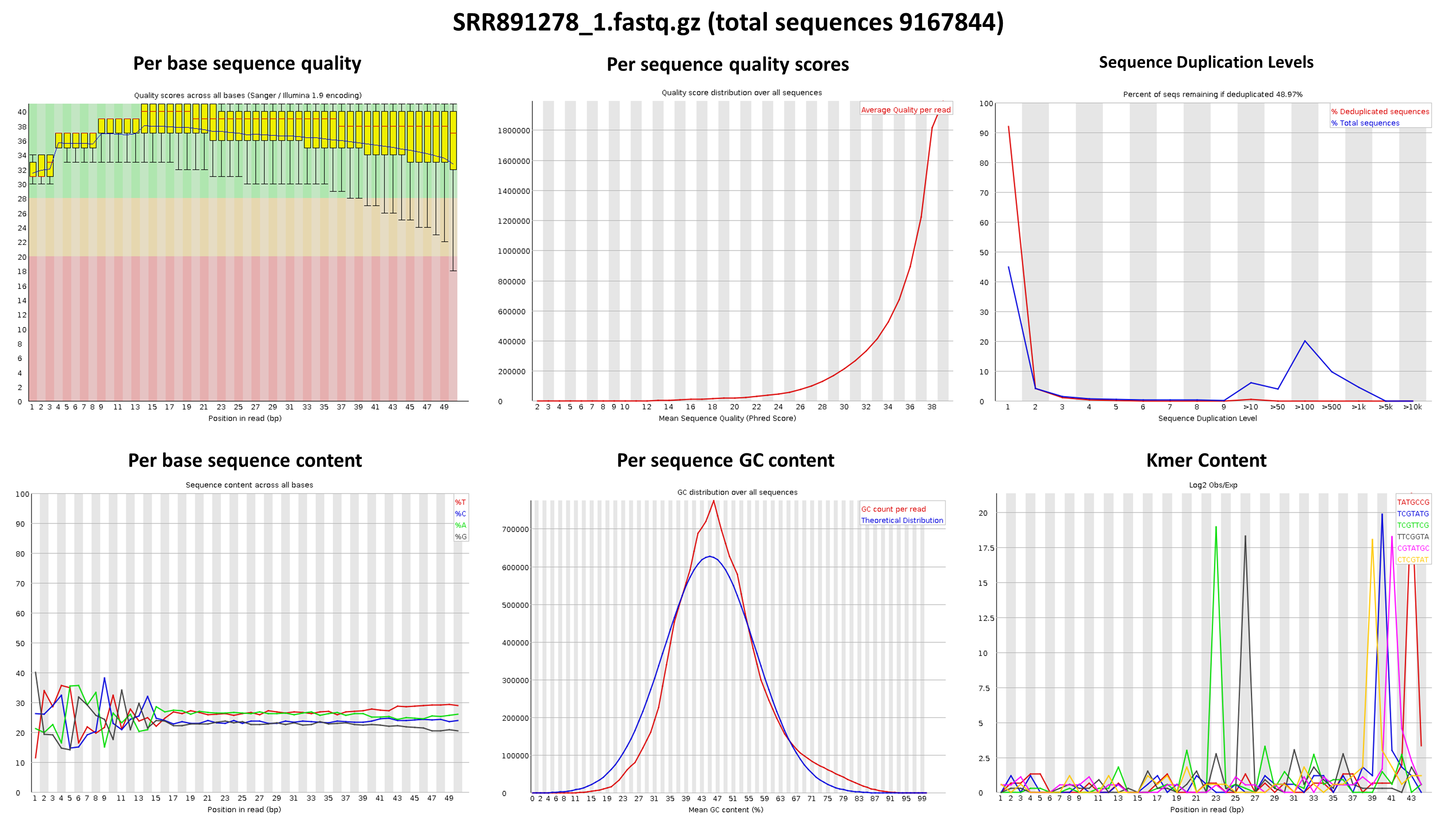


D.

**Supplementary Figure 1. FASTQC produced results presenting the quality of CD4+T samples.** Figure presents the statistics of publically available, downloaded CD4+T samples (SRR891275 (Fig. 6A), SRR891276 (Fig. 6B), SRR891277 (Fig. 6C), SRR891278 (Fig. 6D)) produced by the FASTQC application, which includes *Per base sequence quality, Per sequence quality scores, Sequence Duplication Levels, Per base sequence content, Per sequence GC content, Kmer Content*.


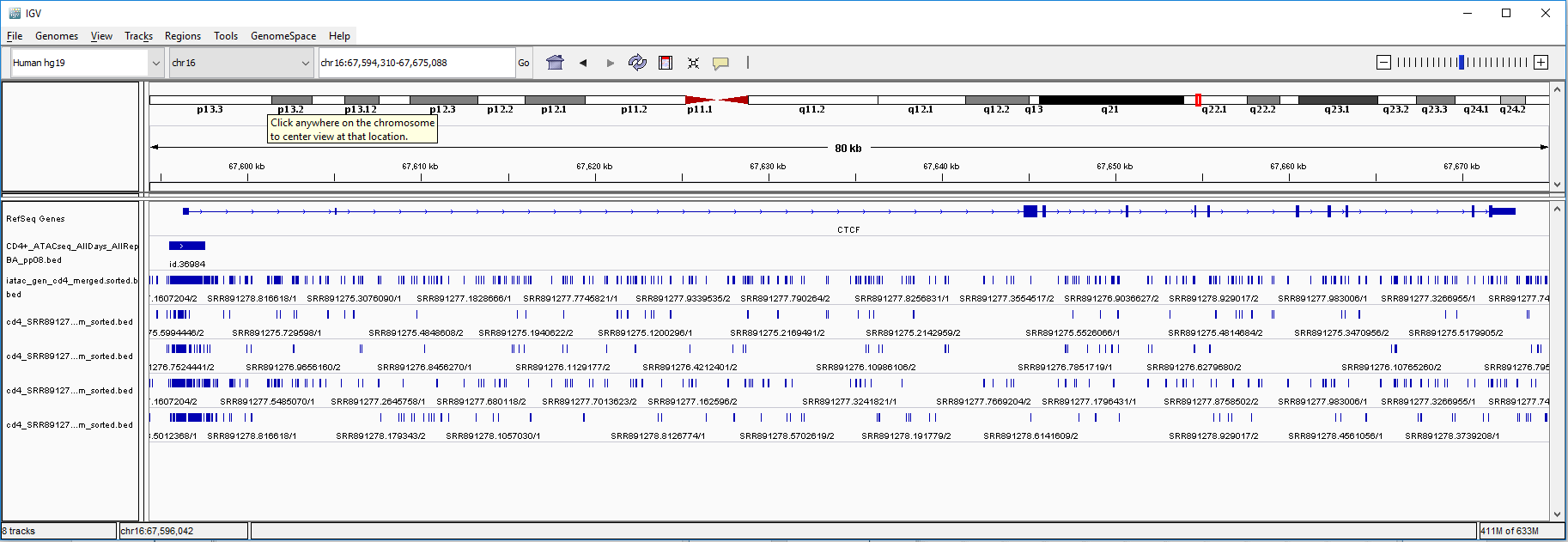


A


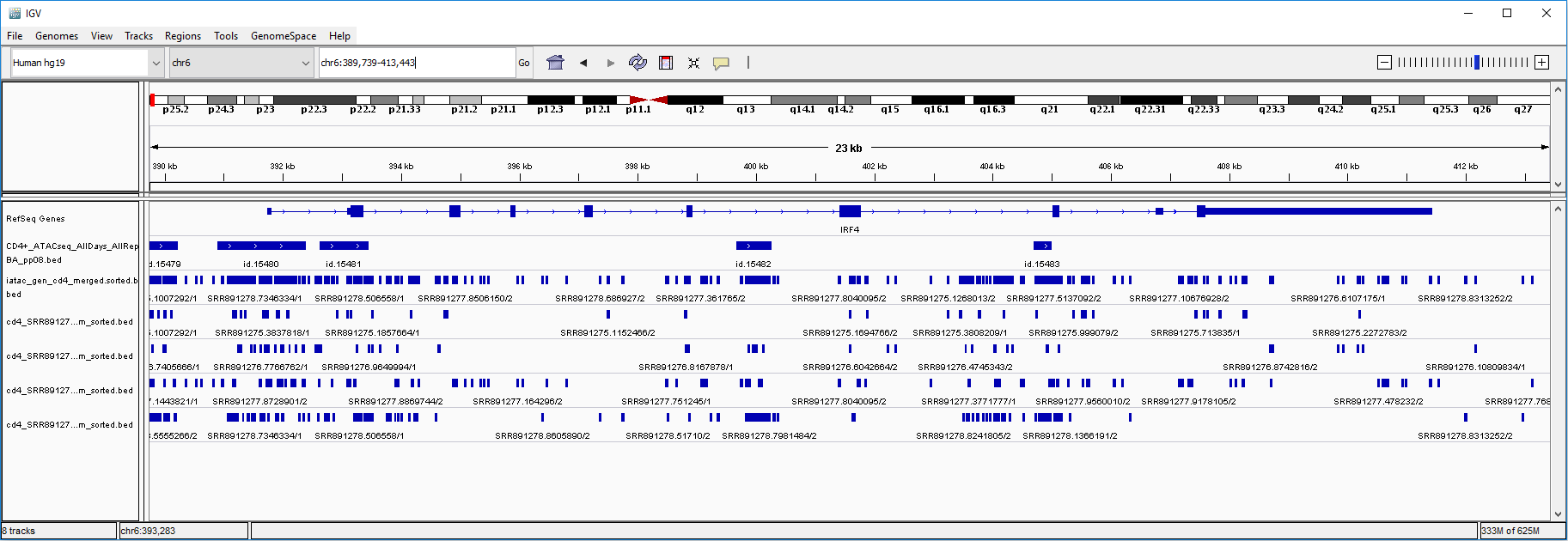


B


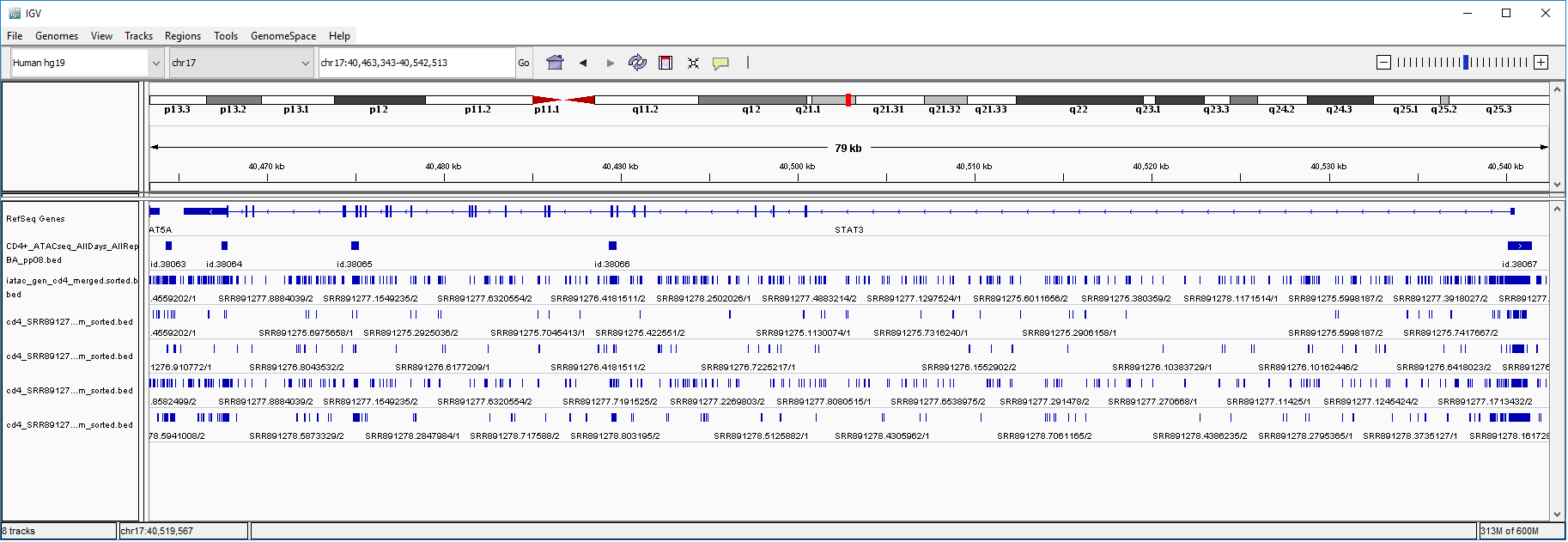


C


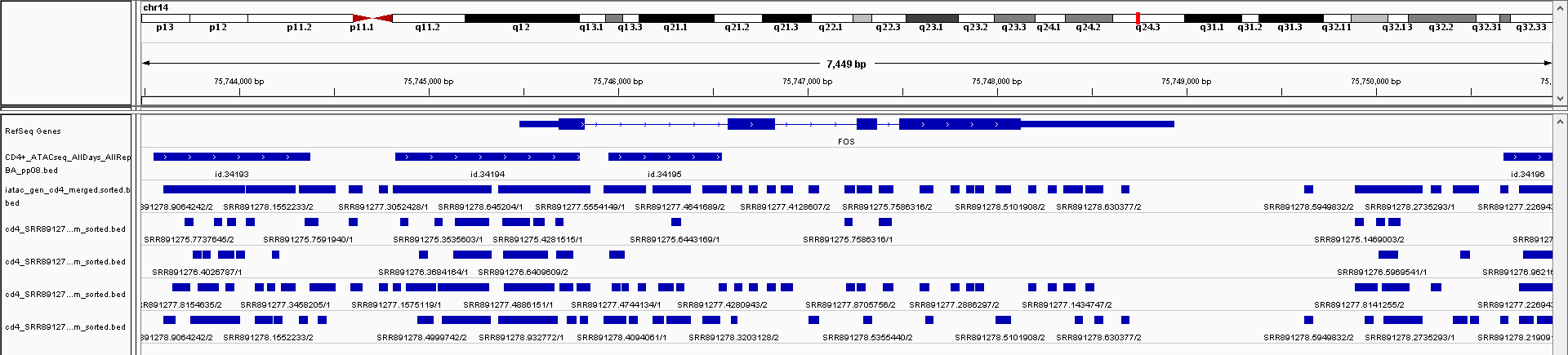


D


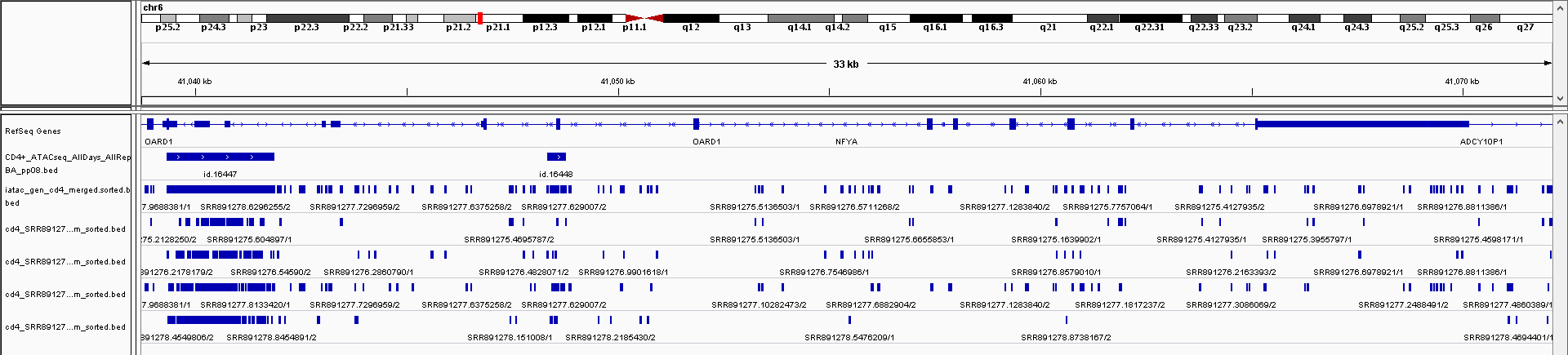


E


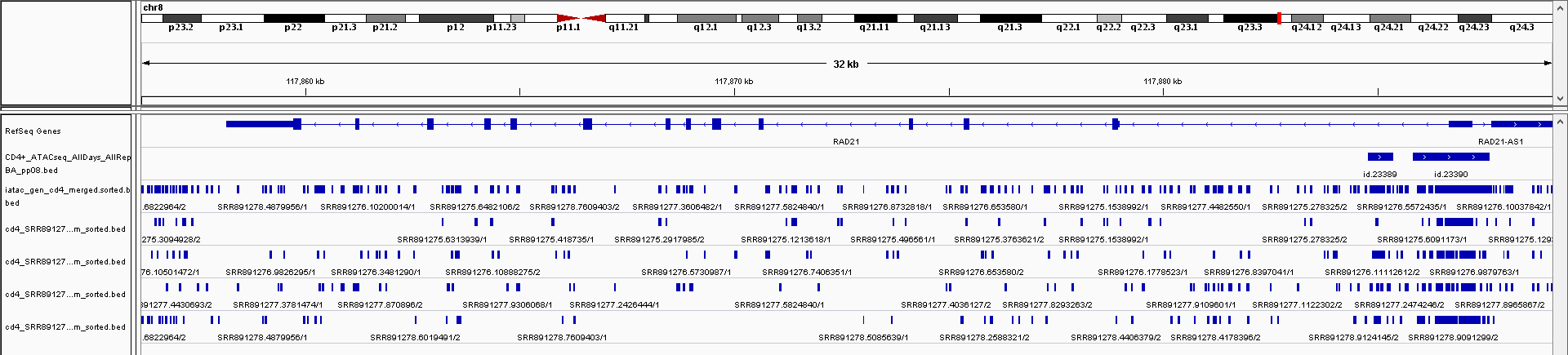


F

**Supplementary Figure 2. Comparison of merged BED of CD4+T samples and I-ATAC generated BED files (Manuscript Figure 7).** Figure presents comparative analysis based on genes CTCF (Fig 7A), IRF4 (Fig 7B), STAT3 (Fig 7C), FOS (Fig 7D), NFYA (Fig 7E) and RAD21 (Fig 7F), among downloaded merged BED file of CD4+T samples and I-ATAC generated individual sample’s generated BED file as well as the one with merged replicated.


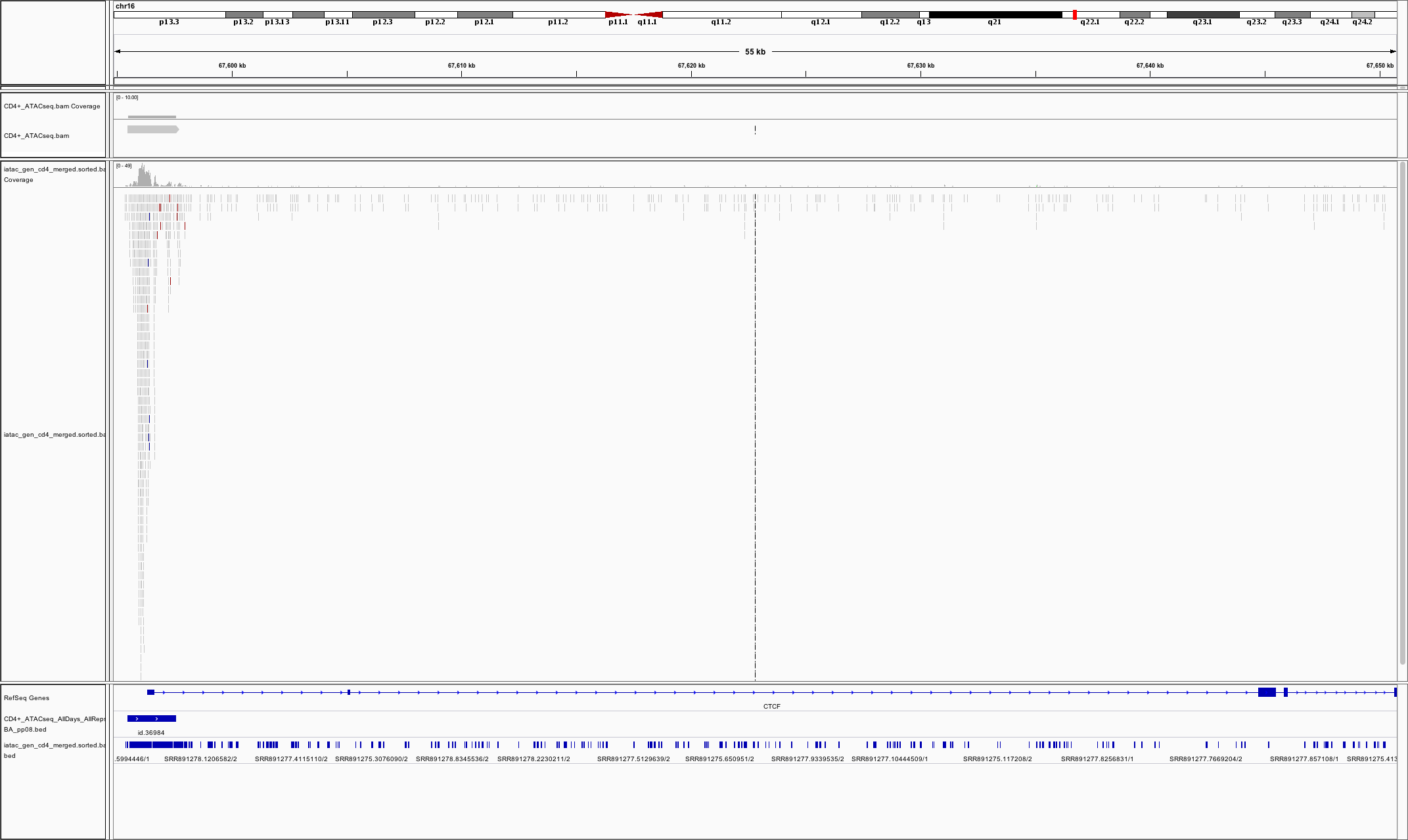


A.


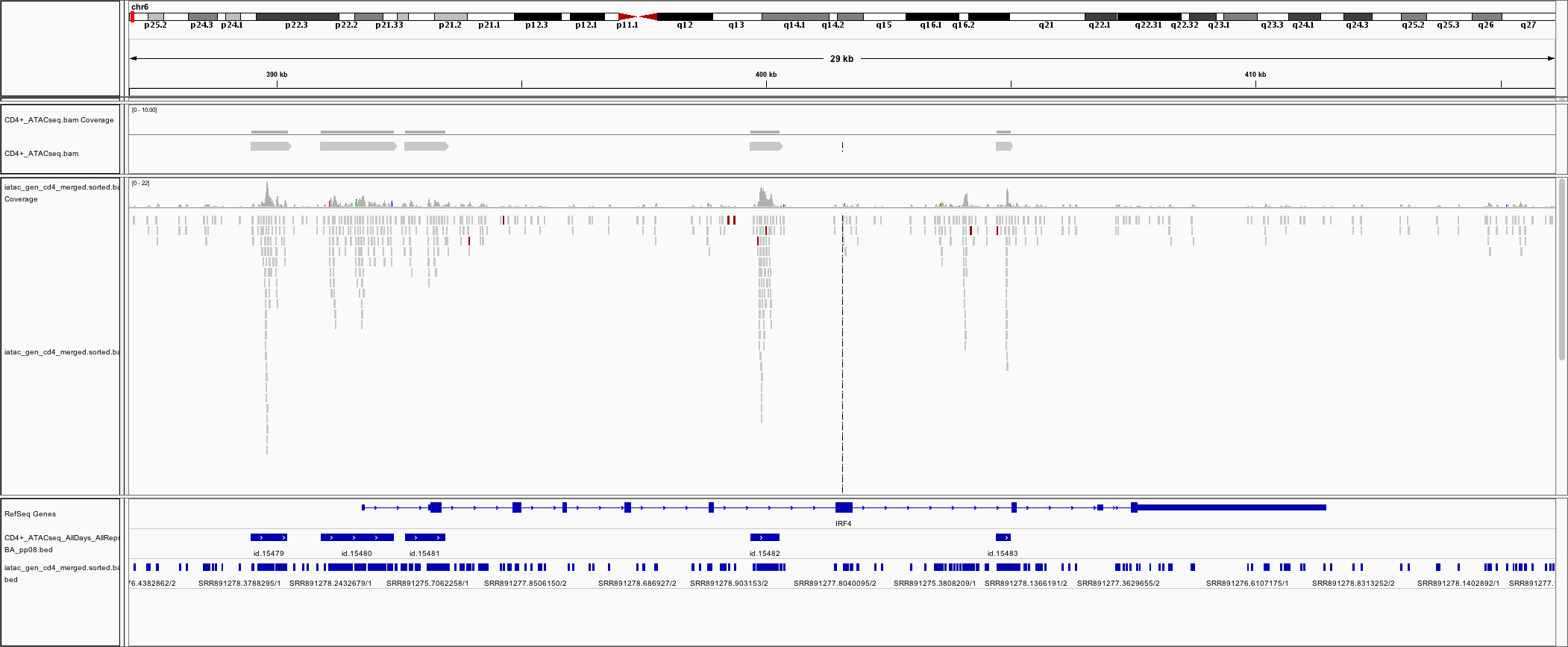


B.


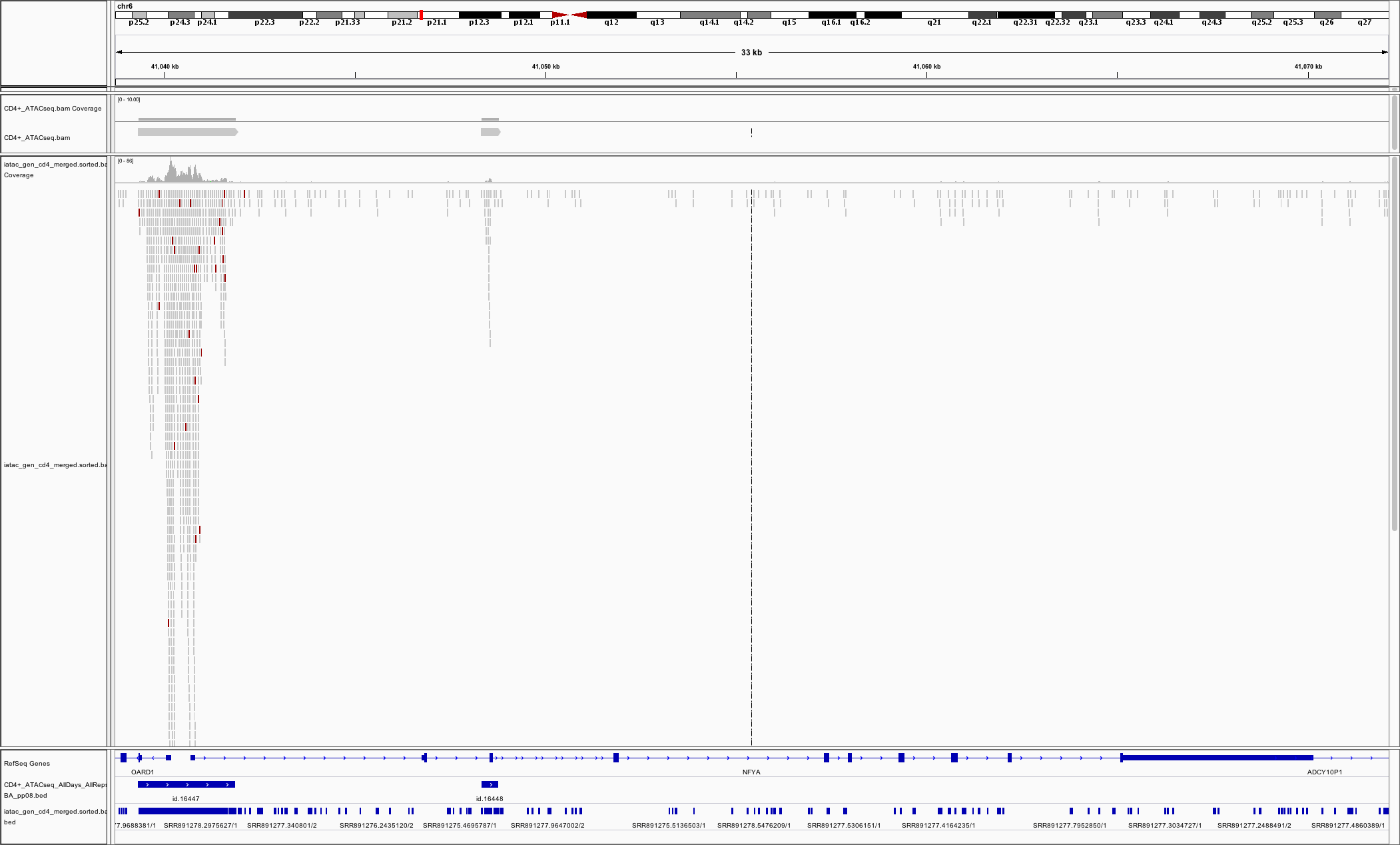


C.


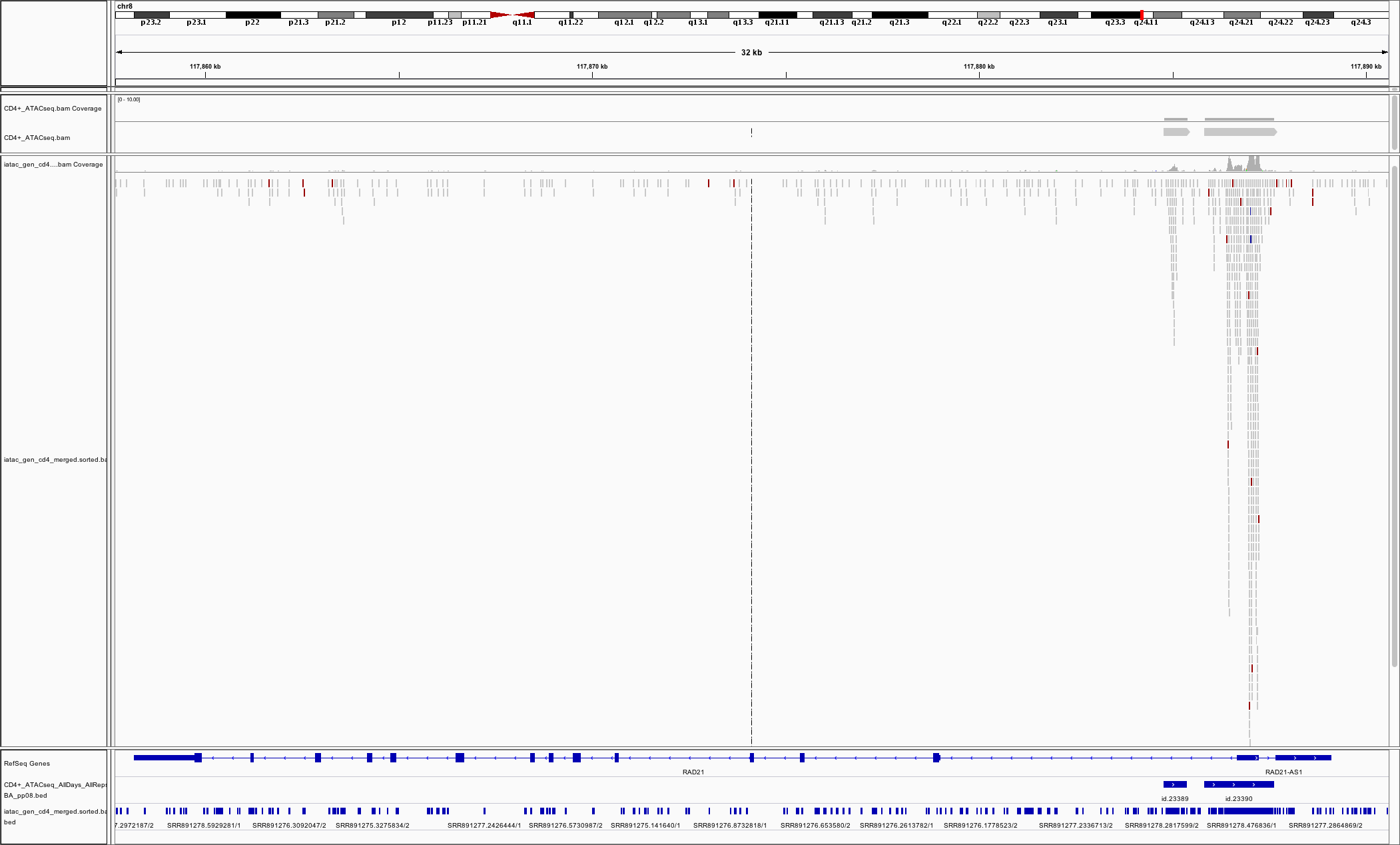


D.

**Supplementary Figure 3. Coverage of merged BED of CD4+T samples and I-ATAC generated BED files (Manuscript Figure 8).** Figure presents, CTCF gene ID 36984 (Fig. 8 A), IRF4 gene IDs 15479-82 (Fig. 8 B), NFYA gene ID 16447 and 16448 (Fig. 8 C), and RAD21 gene ID 23389 and 23390 (Fig. 8 D) have coverage between publically available and our processed datasets.
